# PROTEOFORMER 2.0: Further Developments in the Ribosome Profiling-assisted Proteogenomic Hunt for New Proteoforms*[Fn FN2]

**DOI:** 10.1074/mcp.RA118.001218

**Published:** 2019-04-30

**Authors:** Steven Verbruggen, Elvis Ndah, Wim Van Criekinge, Siegfried Gessulat, Bernhard Kuster, Mathias Wilhelm, Petra Van Damme, Gerben Menschaert

**Affiliations:** ‡BioBix, Lab of Bioinformatics and Computational Genomics, Department of Mathematical Modeling, Statistics and Bioinformatics, Faculty of Bioscience Engineering, Ghent University, Ghent, Belgium; §VIB-UGent Center for Medical Biotechnology, Ghent, Belgium; ¶Chair of Proteomics and Bioanalytics, Technical University of Munich, Munich, Germany; ‖SAP SE, Potsdam, Germany; **Department of Biochemistry and Microbiology, Faculty of Sciences, Ghent University, Ghent, Belgium

**Keywords:** Proteogenomics, Ribosomes*, Tandem Mass Spectrometry, Quality control and metrics, Chromatography, mQC, Prosit, proteoform, PROTEOFORMER, ribosome profiling

## Abstract

PROTEOFORMER is a pipeline that enables the automated processing of data derived from ribosome profiling (RIBO-seq, *i.e.* the sequencing of ribosome-protected mRNA fragments). As such, genome-wide ribosome occupancies lead to the delineation of data-specific translation product candidates and these can improve the mass spectrometry-based identification. Since its first publication, different upgrades, new features and extensions have been added to the PROTEOFORMER pipeline. Some of the most important upgrades include P-site offset calculation during mapping, comprehensive data pre-exploration, the introduction of two alternative proteoform calling strategies and extended pipeline output features. These novelties are illustrated by analyzing ribosome profiling data of human HCT116 and Jurkat data. The different proteoform calling strategies are used alongside one another and in the end combined together with reference sequences from UniProt. Matching mass spectrometry data are searched against this extended search space with MaxQuant. Overall, besides annotated proteoforms, this pipeline leads to the identification and validation of different categories of new proteoforms, including translation products of up- and downstream open reading frames, 5′ and 3′ extended and truncated proteoforms, single amino acid variants, splice variants and translation products of so-called noncoding regions. Further, proof-of-concept is reported for the improvement of spectrum matching by including Prosit, a deep neural network strategy that adds extra fragmentation spectrum intensity features to the analysis. In the light of ribosome profiling-driven proteogenomics, it is shown that this allows validating the spectrum matches of newly identified proteoforms with elevated stringency. These updates and novel conclusions provide new insights and lessons for the ribosome profiling-based proteogenomic research field. More practical information on the pipeline, raw code, the user manual (README) and explanations on the different modes of availability can be found at the GitHub repository of PROTEOFORMER: https://github.com/Biobix/proteoformer.

## Abstract (precis)

The PROTEOFORMER pipeline feeds ribosome profiling-driven information into an MS/MS search space. The pipeline has been greatly expanded and updated since its first publication. These novelties are presented and validated with matching MS/MS data, leading to the endorsement of a set of new proteoforms on MS/MS level and to a collection of general considerations for the ribosome profiling-based proteogenomics community.

## Graphical Abstract

**Figure:**
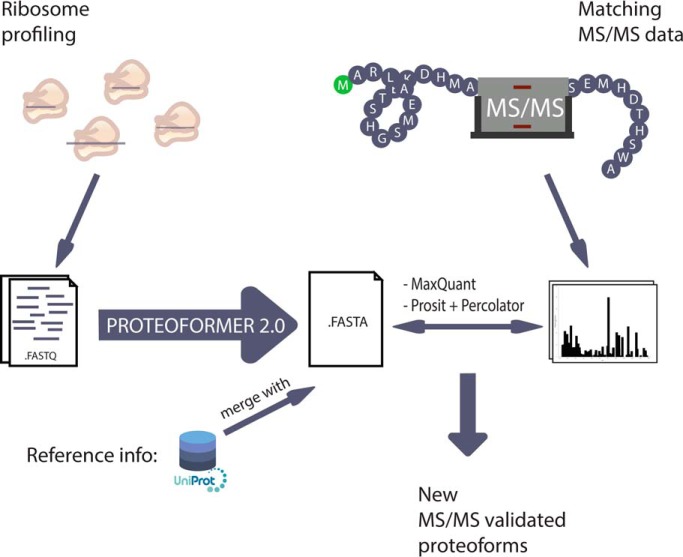

**Highlights**

- PROTEOFORMER adds ribosome profiling information to MS/MS search spaces.
- The PROTEOFORMER pipeline is greatly expanded and updated since its first publication.
- New features are demonstrated with matching ribosome profiling and MS/MS data.
- Experiments lead to MS/MS-proven proteoforms and general proteogenomic notices.

Proteogenomics describes the field where mass spectrometry (MS) based proteomic research is combined with next generation sequencing (NGS)^1^ based genomics, transcriptomics and translatomics (1). It is an evolving field where new tools are continuously proposed and where the discussions about best practices are still ongoing in its community (http://psidev.info/proteomics-informatics-standards-working-group-charter). Main aims of the research are the improvement of gene annotation and the analysis of the proteome complexity (2).

To expand our knowledge about proteome complexity, data from sequencing technologies can be used to construct a custom database for subsequent MS searches. For example, introducing RNA-seq results into the search space aided in identifying splice variants (3) and single amino acid variants (SAVs) at the proteomic level (4). Ribosome profiling (5, 6) takes this approach even a step further. With this recent technique, ribosome-protected mRNA fragments are analyzed with NGS leading to a genome-wide measurement of the translation landscape. Typically, ribosomes are halted on the position where they are translating the mRNA by using an antibiotic. In Eukaryotes, the antibiotic cycloheximide (CHX) stabilizes ribosomes on the mRNA sequence and prevents further ribosomal translocation, allowing the study of elongating ribosome profiles. Other antibiotics like harringtonine (HARR) or lactimidomycin (LTM), each with their characteristic mode of action, have the unique ability to only stabilize initiating ribosomes, opening up the opportunity to visualize translation initiation (7). After antibiotic treatment, samples will be treated with nuclease so that only the ribosome-protected fragments (RPFs) will remain and can be analyzed with NGS (5, 6). After mapping the sequenced fragments to the reference genome, specific offsets allow to pinpoint the alignments onto the P-site (*i.e.* the exact base position where the ribosome was translating the mRNA into a peptide product). The offset in between the 5′ end of the read and the P-site depends on the length of the RPF. With a correct set of P-site offsets, the subcodon resolution can be correctly disclosed and important features of ribosome profiling, like triplet periodicity and translation patterns, can be unveiled.

Ribosome profiling gathers data closer to the stage of the final protein product than RNA-seq and as such, it serves as a better protein expression proxy for expanding the MS search space with sample-specific sequencing results. With the help of a ribosome profiling extended search space, alternative initiation events could be validated with matching MS data (8–10). A few years ago, we devised PROTEOFORMER (11), a complete pipeline for processing ribosome profiling information into a sequence database for MS-based validation. This allowed to identify new protein forms (proteoforms) and helped the re-annotation of genomes.

Several other proteoform-predicting tools (12–17) were devised over the last years, of which two are of interest for this manuscript in particular. The first one is PRICE (12) which infers open reading frames (ORFs) by modeling the experimental noise and the stochastic processes involved in RIBO-seq. From this model, the set of translated codons that generates the observed reads with maximum likelihood is determined. This is in turn the basis for the assembly of ORF candidates. Another new interesting tool is SPECtre (13). It focuses on modeling the triplet periodicity of ribosomal signals using a spectral coherence classifier. It is important to note that these two new techniques can function without the use of a parallel initiation profile sample, a hallmark that was lacking in the former PROTEOFORMER pipeline (11).

Different search engines are available for matching the tandem mass spectrometry (MS/MS) fragmentation spectra to the different peptides in the sequence database. SearchGUI (18) provides a user interface for combining different search engines. The MaxQuant tool (19), with the Andromeda search engine included (20), additionally includes quantitative analyses over multiple samples at once.

For most MS search engines, only the number of fragment ion matches is considered when comparing theoretical and experimental spectra. Nevertheless, it has been proven that adding intensities to the matching algorithm (MS/MS intensity-based proteomics) enhances the identification rates (21–23). Recently, Prosit allowed to raise identification rates based on the application of deep learning on the protein-to-spectrum matches (PSMs) (24). Prosit will start off by predicting fragment intensities for the candidate PSMs. Based on these predictions, additional PSM features can be derived. Percolator (25, 26), on the other hand, is a MS post-processor tool, enabling to combine features and scores of different MS analysis tools. Based on a semi-supervised learning method with support vector machines, it provides a statistical framework to interpret the combined results. Percolator is thus able to join the additional features of Prosit with the results of canonical search engines.

Here we present all new features added to the PROTEOFORMER pipeline since its first publication. Ribosome profiling mapping, data pre-exploration, proteoform calling strategies and outputted features have been extensively improved and expanded. Based on multiple high-depth samples of matching MS/MS data of HCT116 and Jurkat human cell lines, the ribosome profiling-based sequence database was searched with MaxQuant. On top of the classical MS/MS search engines, Prosit was applied in combination with Percolator to enhance the peptide identification rate and provide extra confidence for (novel) proteoform events and genome re-annotation.

We first give a short technical overview of the new features in the experimental procedures before setting out the results of the HCT116 and Jurkat case studies. Afterwards, we discuss the implications of these new features and results on the general proteogenomics research field.

## EXPERIMENTAL PROCEDURES

### 

#### 

##### PROTEOFORMER Pipeline

**Fig. 1.**
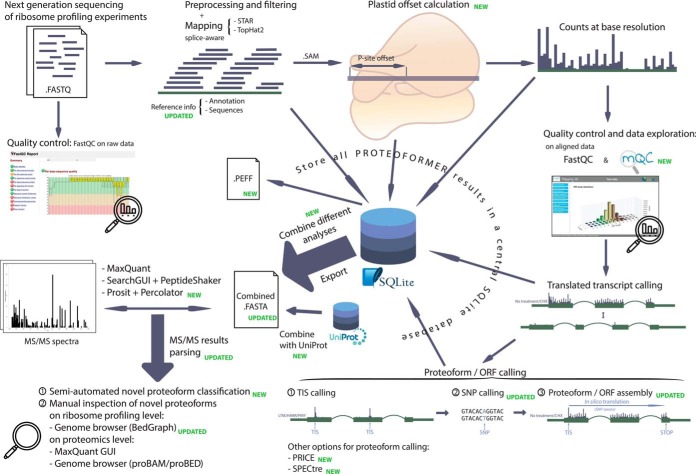
**Most important parts of the PROTEOFORMER pipeline workflow.** The pipeline starts with raw reads from a ribosome profiling experiment, provided in FASTQ format. The quality of these raw reads are checked with FastQC (40). Next, the reads are preprocessed, filtered and mapped to the reference genome. By using P-site offsets (calculated with Plastid (28)), these alignments can be pinpointed at the base level. Quality of the alignments and general data outlook will be checked with help of FastQC (40) and mQC (29). If the user is satisfied with the output, one can continue with the pipeline. PROTEOFORMER will search for the transcript isoforms with translation evidence. Based on these, the translated proteoforms can be deduced. The workflow used in the previous PROTEOFORMER (11) version can be applied (TIS calling, SNP calling and proteoform assembly) or one can use PRICE (12) or SPECtre (13) to determine these proteoforms. All results of these earlier steps are saved in an SQLite results database. For MS-based validation, the results can be exported, combined and even merged with canonical information from UniProt. The end result is a FASTA file that can be used for database searching of MS/MS spectra with tools like MaxQuant (19), SearchGUI-PeptideShaker (63, 64) or Prosit (24) in combination with Percolator (25). Several novel scripts were added to the pipeline to use these search results for counting database hits and classifying new proteoforms and novel translation events in a semi-automated fashion. Identifications can also be manually inspected on both ribosome (*e.g.* by browsing the PROTEOFORMER BedGraph files in a genome browser environment) and MS level (MS software interface or converted MS identification files in proBAM/proBED format (57, 58) in the same genome browser session as the BedGraph ribofiles).

Since its first publication (11), the PROTEOFORMER pipeline has been largely redesigned and multiple novel features have been added (Fig. 1).

First, the installation of the pipeline is now possible using Conda (27) environments with all dependences included, facilitating a much easier installation process for the user. Also, new modules are included making the download of the reference information more streamlined.

Another important set of updates is related to quality and preliminary data visualization, which was underrepresented in earlier versions. This includes the dataset-specific calculation of exact P-site offsets with Plastid (28), a feature that is important for correct base allocation of the alignment and thus also for uncovering ribosome profiling hallmarks like triplet periodicity (28, 29). Earlier versions of PROTEOFORMER could only use a fixed set of default offsets. Also, users now can verify the quality and general outlook of their mapped RIBO-seq data more thoroughly as mQC (29) is directly embedded into the PROTEOFORMER pipeline. A more prominent position for FastQC (40) in the general analysis workflow is also foreseen.

A big shortcoming of the previous version was that the pipeline always needed the combination of an elongating ribosome profile (an untreated sample or treated with CHX) and an initiating ribosome profile (a sample treated with HARR or LTM). Several studies only contain the elongating profile and therefore, two new proteoform calling techniques are extra implemented into the pipeline wherefore this initiating profile is not necessary: PRICE (12) and SPECtre (13). Meanwhile, the already present proteoform calling combination of translation initiation site (TIS) and single nucleotide polymorphism (SNP) determination (also denoted as “TIS and SNP calling”) and proteoform assembly (for this manuscript simply termed as “classic proteoform calling”) is also updated. If initiation profiling is available, an extra advantage is that the three methods can be compared and used to complement one another. If the initiation profile is absent, PRICE and SPECtre can still be combined.

Great effort is put in combining the results of different analyses. PROTEOFORMER is therefore now able to for instance merge the translation products identified by the three different proteoform calling techniques, eventually with removing sequence redundancy. Further more, tools are included to further merge results with sequences from UniProt (30). A code system (explained in more detail in the supplemental methods) is added to the accessions in the produced total FASTA file, allowing to track what source(s) (PROTEOFORMER or UniProt) and which method(s) or TIS id(s) a sequence is originating from.

The PROTEOFORMER pipeline now also exports results in the new PSI extended FASTA format (PEFF) (http://www.psidev.info/peff). This format provides tags to store SNP and proteoform variations in a more structural way.

New features were illustrated by running the PROTEOFORMER pipeline on human HCT116 colon cancer cells (11) and human Jurkat T lymphocyte cells (31). The three different proteoform calling techniques (classic, PRICE and SPECtre) were applied and results were combined into one FASTA file. The latter was then merged with reference information from UniProt to obtain one comprehensive database in which both canonical products as new proteoform candidates are represented.

A more detailed explanation on the pipeline and its usage in this study can be found in the supplementary experimental protocols. A general manual of the PROTEOFORMER pipeline is available on GitHub (https://github.com/Biobix/proteoformer/blob/master/README.md). We also included an overview of all commands we used to generate our data (examples_commands.md).

##### Matching Mass Spectrometry Analysis

Matching LC-MS/MS data were generated in HCT116 and Jurkat cells using a Q Exactive HF instrument. Obtained proteomics data were matched against the comprehensive database from PROTEOFORMER using the MaxQuant graphical user interface (GUI) (19). Additional scripts are added to PROTEOFORMER to parse these MaxQuant results together with the code system that is added to the FASTA file accessions. As such, the share of each proteoform calling method in the final proteomics identification results was rated. Further, this tracking system also allowed to determine the origin of identifications. Identifications of sequences provided by PROTEOFORMER but not yet included in UniProt offered evidence for new proteoforms. Another new script classifies the new proteoforms based on the nature of their variation. This novel feature thus semi-automatically allows to determine the nature of novel translation events, which is mostly the goal of proteogenomics strategies.

Fragment intensity prediction with Prosit (24) was applied on the PSMs. This allowed calculating additional spectral features. Percolator (25, 26) was then used to combine the different scores and features from both Prosit and MaxQuant. Q values of all features (Prosit and MaxQuant) were compared with q values of only MaxQuant features (Andromeda score and delta score).

##### Supplemental Experimental Procedures

Extensive experimental procedures can be found in the supplemental materials.

## RESULTS

### Ribosome profiling analysis with PROTEOFORMER

#### 

##### Data Quality Assessment, Read Preprocessing, Alignment and Translated Transcript Calling

The updated PROTEOFORMER pipeline (Fig. 1) was applied on matching RIBO-seq and shotgun proteomics data originating from two cell lines, human HCT116 (11) and Jurkat T cells (31). For both cell lines, the CHX-treated (elongating ribosome profile) and LTM-treated (initiating ribosome profile) samples were quality checked with FastQC. The quality reports of the raw HCT116 CHX- and LTM-treated, and Jurkat CHX- and LTM-treated read samples are respectively given in supplementary Files S1 and S2, and S3 and S4. Based on several plots (*i.e.* GC content, adaptor content, duplication levels) preprocessing and filtering of the reads is desirable. Therefore, the raw reads were quality-trimmed and adaptors were clipped. Consecutively, the reads were prefiltered against databases containing rRNA, tRNA, sn(-o-)RNA sequences and the PhiX bacteriophage genome. Finally, preprocessed reads were aligned to the human genome. For comparison purposes, Jurkat alignments were only allowed if they map to one unique position in the genome, whereas HCT116 alignments can map to up to 16 positions. Mapping statistics of both prefiltering and genomic alignment can be found in supplemental Table S1. The obtained alignment SAM file was then again checked for quality with FastQC. Results for the aligned HCT116 CHX- and LTM-treated and the Jurkat CHX- and LTM-treated samples are respectively given in supplementary Files S5, S6, S7 and S8. General quality improves drastically and overall, contaminants and wet-lab related artifacts (*e.g.* translation inhibitor usage (32), ribonuclease treatment (33), composition of the lysis buffer (34)) seem to be removed. This quality enhancement is caused by the application of adapter clipping, quality trimming and filtering before mapping to the genome as has been described previously (6). We recommend to visually check the effects of these steps with FastQC, as embedded into PROTEOFORMER. Further, visualizing quality and metagenomic features is in our opinion good practice to have a general understanding of the data content before continuing with the rest of the analysis.

Besides, FastQC reports a slightly better quality for Jurkat than for HCT116, but one must bear in mind that the read coverage of Jurkat is around 2,5 times higher.

Ribosome profiling specific data exploration was done with mQC. The results for the CHX- and the LTM-treated HCT116 samples are respectively given in supplementary Files S9 and S10. For the CHX- and LTM-treated samples of Jurkat, plots are respectively available in supplementary Files S11 and S12. In these files, the results of the Plastid P-site offset calculation are shown as well. Overall, the P-site determination of LTM is crispier than for CHX. Also, the higher coverage in Jurkat allows a more precise offset calculation. In the metagenic annotation plots of HCT116, a quite notable percentage of the alignments lies in processed pseudogenes, because of the non-unique mapping (this percentage reduced drastically when mQC was applied on unique mapped HCT116 data (results not shown)). In general, results of mQC comply with what can be expected (29) and a good triplet periodicity is observable for both cell lines.

Visualization on a more focused level is now possible by loading the generated BedGraph files into a genome browser, with the new option to generate RPF-specific BedGraph files.

The rule-based transcript calling was used for all analyses. During this calling, transcripts are recognized as truly translated if at least 85% of its exons have an elongated ribosome profile coverage higher than a predetermined threshold (more details on this can be found in (11)). In HCT116, it yielded 65 553 translated transcript isoforms. For Jurkat, 82 065 translated transcript isoforms were called.

##### Proteoform Calling

**Fig. 2.**
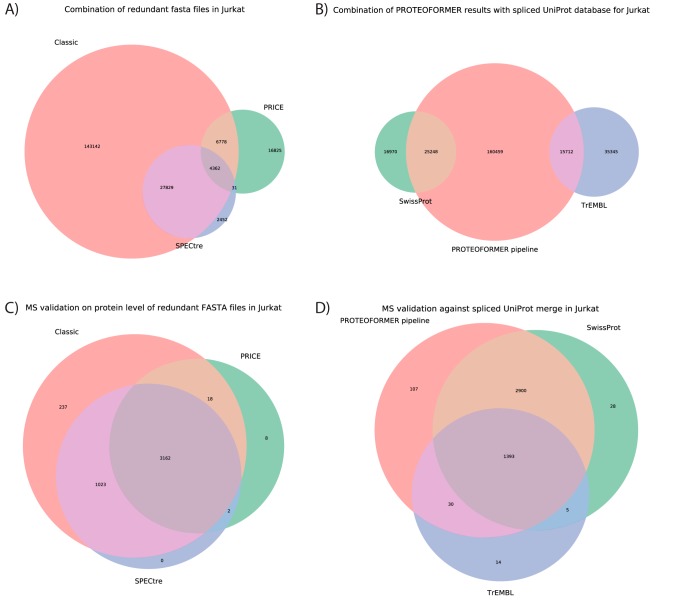
**Overview of the PROTEOFORMER analysis and MaxQuant identification results for Jurkat data.** Different proteoform calling methods were tested and combined in one FASTA file without removing redundancy during PROTEOFORMER run time. Sequence counts and the overlap between methods is shown in panel A. This combined FASTA file was afterwards merged with UniProt (consisting of SwissProt and TrEMBL) and the overlap is given in panel B. The search spaces of respectively A and B were afterwards searched with MaxQuant, leading to the results displayed in respectively panels C and D. The amounts of MS/MS identifications on protein level for each proteoform calling technique are given in C. The distribution of the MS/MS identifications between PROTEOFORMER and UniProt are given in D. The areas of the Venn diagrams in subfigures C and D are log_10_ (x + 1) transformed for better visual representation. The Venn diagrams for all other analyses are given in supplemental Figs. S1, S2 and S4–S6.

In this study, three methods of proteoform calling were compared: (a) the subsequent combination of TIS calling, eventual SNP calling and proteoform assembly (this combination is termed 'classic proteoform calling' from hereon), (b) by using the PRICE algorithm (12), and (c) by using the SPECtre algorithm (13). The two latter methods are novel introductions in the pipeline and have the big advantage that they do not require initiating ribosome profiles. This comes in handy as many recent ribosome profiling studies lack this translation initiation focused experiment (35). The SPECtre method uses a reference annotation in a GTF file as a basis for its analysis and is therefore not useful to find completely novel proteoforms. However, it is useful to check which canonical sequences show translation. A big drawback of this method though, is its running time (149 h), which is significantly longer than for the other two methods (2–4 h). These run times were measured on 20 2.3GHz AMD Opteron™ processors on a Linux server running Fedora Core 23 with 350Gb of RAM. PRICE, the other new method, is constructed to find new translated sequences solely based on ribosome profiling. Therefore, a score model needs to be used. The developers chose a default FDR of 10%. This means that it needs to allow quite some false positives to find its candidates (more details on the PRICE FDR calculation can be found in the online methods of (12)). A less stringent FDR is for this pipeline less a concern though as ribosome profiling is used here to obtain ORF candidates. Stronger validation follows afterwards in the pipeline using MS/MS data. A looser default FDR threshold means though that a lot of canonical sequences are missed, which is why the overlap with the other two techniques (and especially with SPECtre) is relatively small (Fig. 2*A* and supplemental Fig. S1). This observation is extendable to the MS level (Fig. 2*C* and supplemental Fig. S4), as PRICE also lacks at that level quite a big part of the sequences that the other two techniques do capture. The major part of the MS identified sequences missed by PRICE but picked up by classic proteoform calling and SPECtre, start from annotated TISs. Nevertheless, in cases where no MS validation and no ribosome initiation profiling are at hand, the combination of SPECtre and PRICE should result in a complete set of all translated proteoforms based on solely the elongating RIBO-seq profile.

The classic proteoform calling (combination of TIS calling and proteoform assembly) has been upgraded over the years. This method does not work with a score model but is rule-based. Therefore, it is less stringent and thus aims at a subsequent MS validation to exclude the false positives from that phase. In Fig. 2*A* and supplemental Fig. S1, it is shown that the classic proteoform calling gives the most complete search space of the three techniques with a combination of both canonical sequences and new variants. This strategy also adds the most MS identifications attributable to one distinct proteoform calling technique (Fig. 2*A* and supplemental Fig. S4).

Selenocysteines were introduced in the different proteoform calling algorithms. In the classic proteoform calling, respectively 134 (0,057%) and 258 (0,053%) of the candidate translation products contain one or multiple selenocysteines in respectively HCT116 and Jurkat data. With the SPECtre algorithm, respectively 47 (0,127%) and 43 (0,101%) of the candidate products contain selenocysteines. The internal PRICE algorithm does not take selenocysteines into account and just classifies the “UGA” codon as a stop signal. Validation of selenocysteine-containing peptides in MS/MS was however not possible. As these sequences constitute only a very small portion of the search space and as selenoproteins have been reported to only occur in specific conditions (specialized MS strategies were even developed for picking them up (36)) and tissues (37), this was somehow to be expected.

For Jurkat data, SNPs were also included during the classic proteoform calling. 7,15% of the candidate products contain one or more SNPs as compared with the reference sequences. A trial version of PROTEOFORMER was implemented that took indels into account as well. Subsequent MS validation could not confirm any new protein variants by indel addition (unpublished), so for the moment, indel-aware proteoform calling is not included in the pipeline.

##### FASTA File Export and Database Combinations

FASTA files were generated for the three different applied proteoform calling methods for both HCT116 and Jurkat data. Redundancy can be removed when generating these files, so both files with and without remaining redundancy were exported for all methods. Afterwards, the FASTA files of the three different methods were merged into one comprehensive FASTA data set, for either their redundant or nonredundant forms. The overlap between methods found during this merging is shown in Fig. 2*A* and supplemental Fig. S1.

In general, the overlap between the different methods is quite low, but this overlap enlarges tremendously once MS/MS validation is applied (Fig 2*C* and supplemental Fig. S4). It is thus essential to keep in mind that ribosome profiling can lead to a candidate proteoform database, but not to a database of surely present proteins. High overlap is therefore not necessarily expected on ribosome profiling level, in contrast to the MS/MS level.

If redundancy was not removed in the initial database exports, especially the classic proteoform calling method database size is somewhat larger. The redundant database (119,716 sequences) is 55,58% larger than the nonredundant (76,945 sequences) for the classic proteoform calling in HCT116. In contrast, the PRICE and SPECtre databases rise only with respectively 4.33% (14 077 nonredundant sequences) and 13.59% (26,383 nonredundant sequences) when keeping redundant sequences.

The classic proteoform calling of PROTEOFORMER is designed to include different protein variants, also from different TISs (*i.e.* N-terminal proteoforms) in the light of possible MS validation afterwards. Therefore, it initially contains a lot of overlapping sequences (*e.g.* extensions, truncations, splice variants…). SPECtre on the other hand starts from a canonical reference annotation, resulting in almost all identified candidate products starting from a canonical TIS location with almost no new variants. When redundancy is not removed, the sequences resulting from the classic proteoform calling method remain in the database in both their canonical and variant form. Therefore, a higher overlap between SPECtre and the classic method is seen. Also, detailed analysis revealed that none of the PRICE-unique translation product candidates start from a canonical TIS. All canonical TIS-starting candidates identified by PRICE are in the overlap regions with the other two methods (Fig. 2*A* and supplemental Fig. S1).

Another remarkable feature, seen in these plots, is the fact that there is much less overlap between PRICE and the other two approaches than between the classic proteoform calling method and SPECtre. Varying the false discovery rate (FDR) of PRICE (from 0.01 over 0.1 to 0.2) did not result in an overlap increase with the other two methods (results not shown). Specifically, the number of PRICE-unique variants is subject to a changing FDR, increasing with a less strict FDR. Contrarily, the canonical sequences in the overlap sections (with classic and SPECtre) do not change remarkably.

Next, the combined results of the three methods were merged with all protein info from UniProt (consisting of SwissProt and TrEMBL). As the MS analysis program can sort out the different redundant forms later by applying protein inference algorithms, the combined redundant FASTA files were chosen for fusion with UniProt. Besides, the database size does only increase by a factor of 0.43 when keeping redundant sequences. As this is far from an exponential increase, the negative impact on peptide and protein scoring is limited. The overlap between sequences resulting from the PROTEOFORMER pipeline and those available in UniProt is presented in Fig. 2*B* and supplemental Fig. S2. For HCT116, both a version of UniProt with and without splice variants was examined. Splice variant inclusion enlarges the overlap between UniProt and PROTEOFORMER. Also, overlap with PROTEOFORMER is larger for the SwissProt part of UniProt compared with TrEMBL and by including splice variants, this effect is even more pronounced. For the Jurkat data, the overlap has not notably increased compared with HCT116, but the new variants delivered by PROTEOFORMER have expanded because of the higher coverage in this data set. Detailed investigation (based on the underlying SQLite database) reveals that all PRICE-unique candidates are new variants that do not overlap with UniProt. The classic proteoform calling method on the other hand gives a combination of both UniProt known sequences and new variants. SPECtre-unique sequences are mainly found overlapping with TrEMBL whereas sequences shared between SPECtre and classic proteoform calling are generally found overlapping with SwissProt.

Another new useful addition to the pipeline is the new PEFF format, following the definition of this new format by the HUPO PSI. This FASTA-derived format allows grouping of the different SNPs and proteoform variants of a common base sequence more logically together as one entry. An example of the different proteoforms of human transcript ENST00000000412 is given in supplemental Fig. S3. These proteoforms can be exported in PEFF format as can be seen in supplementary File S13, which is a snippet of a full PEFF file generated with PROTEOFORMER.

### MS/MS-based Validation with MaxQuant

Matching high-depth LC-MS/MS data over 4 replicates were searched with the MaxQuant GUI. The full results of these searches, together with peak and raw data, have been deposited to the ProteomeXchange Consortium via the PRIDE (38) partner repository with the dataset identifier PXD011353. Search results at the protein and peptide level are also available in supplementary Files S14–S19.

First, data were searched against the FASTA files of the three combined proteoform calling techniques to see how much each methodology adds to the general identification rate at the MS level. For HCT116, both the combination process with and without the redundancy removal was tested; for Jurkat, only the redundant option was analyzed. A table of identification rates at protein group, peptide and PSM level for the different search spaces and samples can be found in supplemental Table S2. In this table, the high coverage of the MS/MS data is visible in the high amounts of protein groups identified. The results coming out of these MaxQuant analyses can be used to determine the share of each proteoform calling strategy in the pool of MS/MS validated proteins, as shown in Fig. 2*C* and supplemental Fig. S4. It can be observed that preserving the FASTA-level redundancy, tends to reduce the number of SPECtre-only validated sequences. Like the ribosome profiling stage, keeping redundancy leads to more overlap with the classic proteoform calling method, as stated earlier. The higher coverage in the Jurkat ribosome profiling data boosts the overlap between PRICE and the other two proteoform calling methods (classic and SPECtre-based), resulting in the largest number of validations residing in the overall union of the three methods (supplemental Fig. S4*G*). The higher coverage also leads to more classic proteoform called validations.

Peptides with selenocysteines on the contrary, could not be identified with MaxQuant. There are some arguments though that explain why selenocysteine could not be picked up in MS/MS data. First, selenocysteines are present in very low abundance in the ribosome profiling data: 43 ORFs with selenocysteines of the 42,452 ORFs in total (∼0,1%). Second, selenoproteins are known to be tissue specific and susceptible to expression changes as a result of processes like aging (36). Last, very specific methods are developed to enrich and pick up selenoproteins (36, 39). Under these specific enrichment conditions, Guo *et al.* (36) could only pick up 22 known selenoproteins and 5 new candidates in MS/MS data. So, for the MS/MS run analyzed here, not specifically designed to pick up selenocysteine-containing peptides, it is not surprising to not detect these.

In a second round, the previously mentioned PROTEOFORMER pipeline generated sequences were now merged with UniProt sequences and this combined database was used for MS/MS validation with MaxQuant. Identification rates are shown in supplemental Table S2. The shares of the PROTEOFORMER pipeline and UniProt in these MS searches are shown in supplemental Fig. S5. Most of the validations are shared, but most interesting validations are of course to be found in the PROTEOFORMER part not overlapping with UniProt, as these contain MS/MS spectra validating new proteoforms and novel translation events. If the UniProt splice variants are included, roughly 50% of the earlier PROTEOFORMER-only validations can be explained by the added splice variants in the extended UniProt database. It illustrates the benefit of including alternative splice isoforms in the search space. The higher ribosome profiling coverage of the Jurkat data allows to identify two times more validated proteoforms, while the number of shared identified sequences remains roughly the same. The distribution of the UniProt sequences was further split up between SwissProt and TrEMBL in Fig. 2*D* and supplemental Fig. S6. Here, the overlap of PROTEOFORMER with TrEMBL is much smaller than the overlap with SwissProt and also smaller than the overlap between all three collections.

**Fig. 3.**
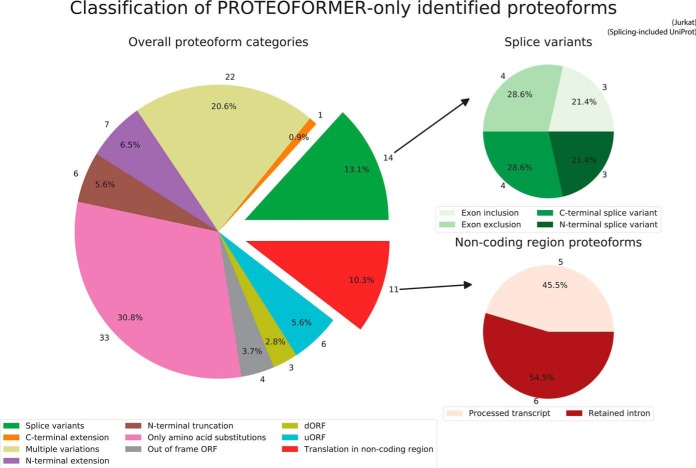
**Classification of the MS-validated proteoforms found in PROTEOFORMER but not in the splicing-included UniProt database for the Jurkat data.** The proteoforms are classified based on the nature of their variation. For new splice variants and proteoforms originating from previously considered non-coding regions, more detailed classifications are added. More information about the different classifications is presented in supplemental Table S3.

**Fig. 4.**
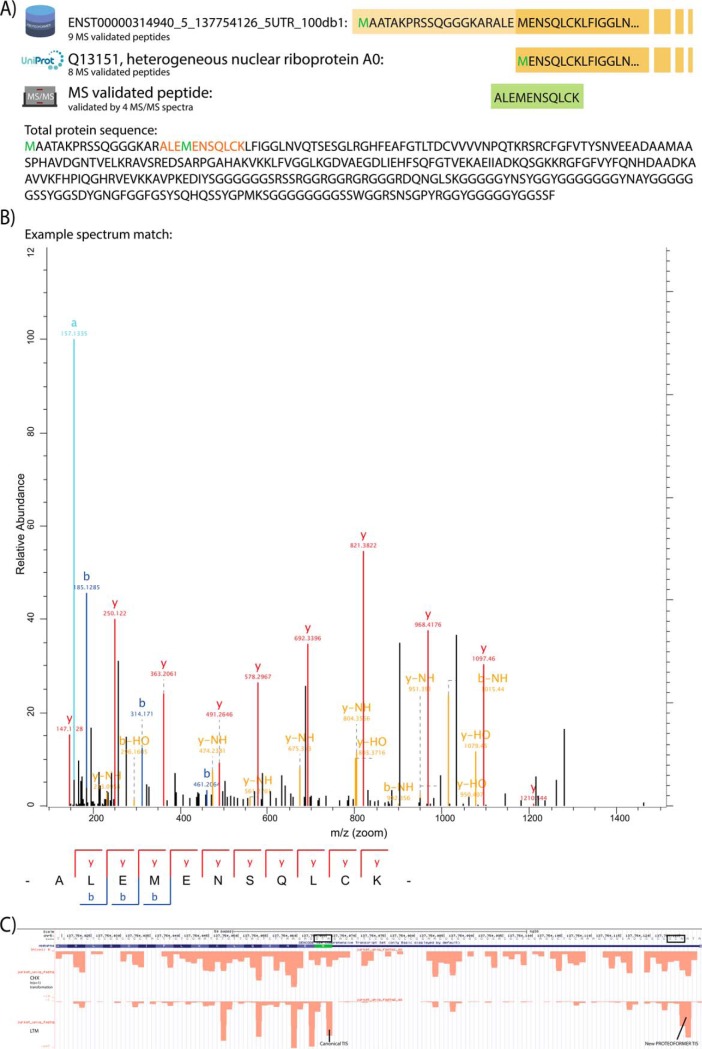
**An N-terminal extension found with PROTEOFORMER and validated with MS/MS data.** *A*, The extended proteoform has one extra peptide mapped, compared with the canonical protein. This extra peptide overlays the canonical initiation site and validates thus the appearance of this N-terminal extended proteoform with MS/MS. The peptide is confirmed with 4 fragmentation spectra of comparable quality. *B*, One of these 4 spectra is given as an example. *C*, View of the N-terminal extension on ribosome profiling level in the UCSC genome browser. The alternative near-cognate TIS (“ACG”) of the N-terminal extension is clearly marked in the LTM data. The CHX data signal is transformed using the following *y* = ln(*x*+1). The triplet periodicity clearly pops up, both in the canonical part as well as in the N-terminal extension of the CHX-treated data. The reading frame is on the antisense strand.

With a new PROTEOFORMER module, the group of MS/MS validated sequences, found by the PROTEOFORMER pipeline but not yet present in UniProt (*i.e.* newly identified proteoforms), was subdivided in more detail based on the nature of their variations. In HCT116, respectively 109 and 52 of such proteoforms are found outside respectively the canonical and splice isoform-included UniProt information. In Jurkat, 107 new proteoforms outside the splicing included reference are found. The classification of these newly validated proteoforms is shown in Fig. 3, supplemental Fig. S7–S8 and in supplementary File S20. Different sources of proteoform variation could be validated: N- and C-terminal extensions and truncations, new splice variants, SAVs, down- and upstream ORFs, out-of-frame ORFs and translation events in previously considered non-coding regions. Splice variants and non-coding region proteoforms could be further classified in subcategories. For HCT116, MS/MS searches against both the merge with the canonical (supplemental Fig. S7) and splicing-included version (supplemental Fig. S8) of UniProt were performed. Comparing the classifications of both experiments points to a reduction of splice variants, C-terminal extensions and N-terminal extensions and truncations in the splicing-included case. The added splicing information in UniProt is thus able to explain parts of certain proteoform categories found compared with the canonical UniProt analysis. Next, the classification for HCT116 (supplemental Fig. S8) and Jurkat data (Fig. 3) can be compared. The different categories present in the analysis of HCT116 data are also present for Jurkat data, but some overall differences are noticeable. An increase of proteoforms with only SAVs is observed for Jurkat data because the SNP calling was additionally executed in Jurkat and not in HCT116. Some of these called SNPs lead to single amino acid substitutions (SAVs), which could be validated in peptides during the MS/MS analysis. Further, whereas for HCT116 there are validated proteoforms found in pseudogenes, for Jurkat, this subcategory is absent. In the metagenic plots of the earlier mentioned mQC reports (supplementary File S9), it was found that the non-unique mapping applied in HCT116 allows enrichment of reads in pseudogenic regions. In Jurkat on the other hand, unique mapping was performed. It is clear that the ribosomal signal in pseudogenic regions for non-unique mapped experiments is also observable in the form of validated pseudogenic peptides at the MS level. An example of a new MS/MS validated proteoform can be seen in Figs. 4*A* and 4*B*, whereas its PROTEOFORMER proof on ribosome profiling level can be visualized on a genome browser as shown in Fig. 4*C*.

### Proof-of-Concept Proteogenomic Experiment Using Prosit and Percolator

For this experiment, the purpose was to try whether there was an added value of extending the scores of Andromeda with other features coming out of the Prosit tool, a neural network approach for fragment intensity prediction. Percolator was used to combine the Andromeda scores with the new features from Prosit.

**Fig. 5.**
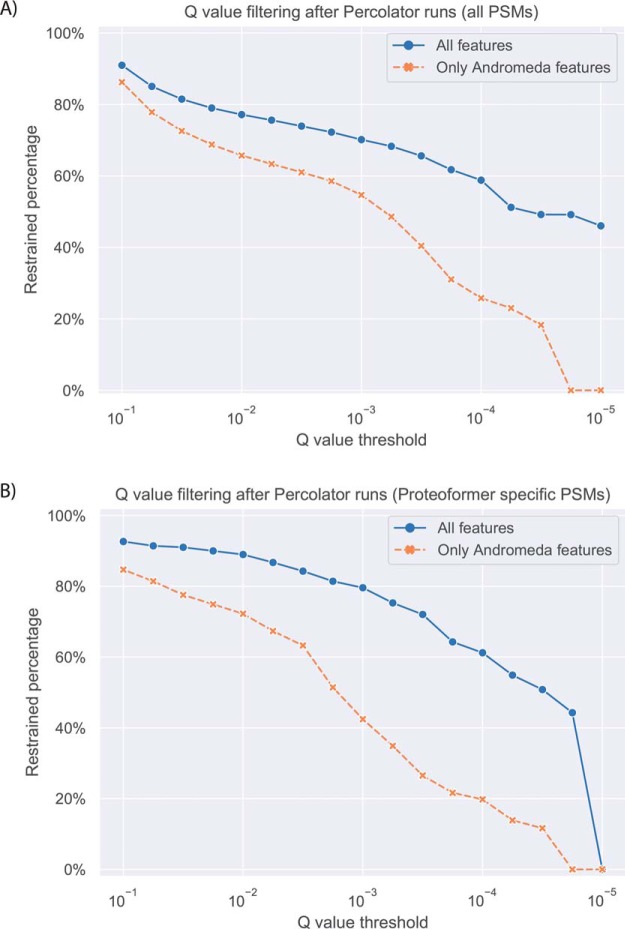
**Comparison of the q values between a Percolator run with only Andromeda scores and a run with combined features from both Andromeda and Prosit.** This analysis was executed for the HCT116 MS data searched against a combined redundant PROTEOFORMER database merged with the canonical version of UniProt. *A*, Analysis for all PSMs earlier identified with MaxQuant. *B*, Analysis of the PSMs earlier identified with MaxQuant because of sequences in the search space resulting solely from the PROTEOFORMER pipeline (*i.e.* novel proteoform events).

A first experiment consisted of comparing the q values of the earlier MaxQuant identified PSMs between a Percolator run with only the Andromeda scores and a second run with a combination of both the scores of Andromeda and the new features from Prosit. This was performed for the HCT116 data with a search space of combined redundant PROTEOFORMER data merged with the canonical version of UniProt (Fig. 5). The number of identified PSMs decreases with more stringent q value filtering. By including not only the scores of Andromeda but also the features calculated by Prosit, it is possible to filter at lower q values. As such, the analysis where Prosit features are included, can be executed at higher levels of stringency while still maintaining a comparable number of validated PSMs which is desired in a proteogenomic setup where the search space tends to increase.

## DISCUSSION

Since its first release (11), a lot of novel implementations were added in the PROTEOFORMER pipeline (Fig. 1). Together with the usage of high coverage MS/MS data, our pipeline leads to the validation of a collection of novel proteoforms. We here want to discuss the overall implications of these novelties on the proteogenomics research field. Further, we want to point out what can be learnt from our approach for the future proteogenomic study of proteoforms and ribosome profiling-assisted re-annotation in general.

### PROTEOFORMER

#### 

##### Data Quality Assessment, Read Preprocessing, and Alignment

Data quality checks and preliminary data exploration hold a very important position in the new PROTEOFORMER pipeline. FastQC (40) offers a very good way to visualize the effects of pre-mapping data clean-up and gives at the same time a metagenomic overview of the data. These aspects are indispensable for the downstream workflow.

The introduction of Plastid (28) improves the base resolution of the alignments to their correct P-site by calculating the RPF length-specific offset based on the sample data. A sample-specific offset is in proteogenomics of course preferable over fixed offsets, as also outlined in (29).

Moreover, the introduction of mQC (29) enables to visualize even more ribosome profiling-specific features of the aligned data. This adds a collection of quality control and general outlook visualization to the PROTEOFORMER pipeline and opens up new ways of visualizing ribosome-specific features like triplet periodicity and codon usage. New quality tools for ribosome profiling start to find ground (29, 35, 41–43) and proteogenomic studies should not hesitate to use them.

Taken together, proteogenomic approaches should wear high priority to quality checks, data exploration and sample-specific P-offsets.

##### Proteoform Calling

As seen in the results section, each of the three proteoform calling methods has its own pros and cons. Depending on the goal of the analysis, one specific method or the combination could be more suitable. In the case of an MS validation afterwards, the combination of all three implemented methods enables a good effort to optimally enrich the search space. If you would have to rely on one technique, the classic proteoform calling takes still the lead with the eye on subsequent MS validation afterwards, on the premise that initiation ribosome profiling data is available. This is not totally unexpected as the classic method is developed to function with subsequent MS (11), in contrast to PRICE (12) and SPECtre (13). Although, if no initiation profiling is available (15, 44–46), PRICE and SPECtre can come in to help. In the future, even other methods (14–17, 47) can be introduced in the PROTEOFORMER pipeline.

There is a lot of discussion on the false positive rate of RPF signals and on how this influences the reliability and significance of ribosome profiling-predicted TISs and ORFs (48, 49), even under stringent settings. Therefore, PROTEOFORMER uses MS/MS as an important independent gold standard technique to validate the new candidates, proposed by ribosome profiling. The discussion on the false positive rate of ribosome profiling demonstrates the necessity of this subsequent MS/MS validation step.

Another point worth mentioning is the fact that information from other transcriptome and translatome sequencing sources could be considered in the PROTEOFORMER pipeline. As RNA-seq sequences can already be mapped with PROTEOFORMER, the foundation stone is already laid for transcriptome mapping. Other translatome sequencing techniques like RNC-seq (50) and TRAP-seq (51) could also be included in PROTEOFORMER in the future once these techniques get as commonly used as ribosome profiling. This would allow constructing candidate proteoform search spaces from different translatomic technical angles. Further, as some of these additional sequencing techniques acquire longer read lengths than ribosome profiling (50), this would open up interesting opportunities for developing pipeline modules to discover new splice variants.

##### FASTA File Export and Database Combinations

Different options were developed for combining the FASTA exports of different PROTEOFORMER analysis strategies as well as for merging with reference sequences from UniProt. The first release of PROTEOFORMER did not allow these merges and users needed to consecutively search spectral data against UniProt first and afterward against the custom PROTEOFORMER database (11). Now, by merging sequences from UniProt and PROTEOFORMER into one database, the proteomics matching is done for all sequences at once in one search run with a set search space size. This eliminates identification biases because of differing search space sizes and thus facilitates the overall evaluation and interpretation. We are convinced that this strategy is useful in lots of other proteogenomic research cases. Further, the devised combination options can also be used to compare other sets of strategies other than the comparison between proteoform calling methods performed in this manuscript (*e.g.* different transcript calling methods, different PROTEOFORMER parameter sets…). On the downside, combined databases lead to bigger search space sizes and this has a mild but negative effect on the proteomics FDR. Therefore, novel MS/MS identification strategies can be applied to overcome this problem (see *infra*).

As some search engines (*e.g.* Comet (52), ProteoMapper (53)), phpMS (54)) start to accept also the PEFF format (http://www.psidev.info/peff), another new option included in PROTEOFORMER allows exporting the results in this new format. The rich and strictly defined PEFF header information, including details about sequence variants, promises to be a helpful tool to communicate precise results easier between different proteogenomic tools (55). The more tools that are programmed to handle this format, the broader the applicability of this novel format will be.

### MS/MS-based Validations with MaxQuant

First, we described MS/MS-based validation experiments with a focus on combining different proteoform calling strategies. Not much difference in the number of MS/MS identifications is observed between the combination with or without search space redundancy removal. This is mostly because the protein inference algorithm of MaxQuant (19, 20) (or other MS/MS identifications tools) bundles the redundant sequences in protein groups. It is however useful to keep the redundancy if there is a merge with UniProt planned afterwards, as the canonical sequences will then not be removed by their eventual longer extension variants. Keeping this redundancy does not mean that the database size explodes exponentially as seen in RNA-seq assisted proteogenomic studies (3, 4). Ribosome profiling still considers only information of one reading frame in contrast to the 3- or 6-frame analysis necessary for RNA-seq (11). As such, the effect on MS/MS search FDR is limited. Further, in supplemental Table S2 it is clear that the number of identifications do not differ significantly between searches against a redundant and a nonredundant search space (4 330 identified protein groups for redundant *versus* 4 322 for nonredundant). So, in contrast to RNA-seq supported proteogenomics, for ribosome profiling-assisted experiments a reasonable redundancy can overall be presented to the protein inference mechanism without major influence. As such, the protein inference can be used as an asset to group redundant identifications in protein groups at the later stage of MS/MS searching.

For the merge between PROTEOFORMER and UniProt, most identifications are, at first glance, found in the overlap. Nevertheless, the MaxQuant protein inference algorithm allows picking up new proteoforms from the combined search space that could not be explained by searching the UniProt database alone. As expected from the nature of the algorithms, these newly identified proteoforms were added to the database by the classic proteoform calling and PRICE methods and not by SPECtre, because of its dependence on reference annotation, making it not suited for detecting new proteoforms. Further, a higher coverage at the ribosome profiling level for Jurkat data compared with HCT116, did result in more translation product candidates in the total search space, but it did not remarkably increase the number of MS/MS identifications (supplemental Table S2). However, the amount of novel proteoforms did roughly double (supplemental Fig. S5: panel S5*G versus* S5*D*), so it can be generally concluded that a higher ribosome profiling coverage (and thus a more comprehensive search space) leads to more novel protein variants without increasing the amount of canonical protein identifications. Besides that, more sequencing depth enhances the quality and the power of the ribosome profiling analysis (56).

Next, in Fig. 2*D*, only 47 proteins out of 4477 (1,05%) are identified because of UniProt solely (SwissProt+TrEMBL). This raises the point whether reference information is still useful and could eventually be substituted completely for custom search spaces in general proteomics experiments. It is worth discussing but one should bear in mind that it takes an amount of time to generate these custom databases from sequencing information, whereas a reference database can be downloaded directly from its repository. Clearly, this is study-dependent and here, the combination of both a ribosome profiling-based search space and a reference is necessary to separate new proteoforms from known cases. For samples and species with no or insufficient protein reference information however, this discussion comes in a totally different light. In that case, a custom database generated based on ribosome profiling is of very high value as this custom database can fulfill the role of the deficient reference.

Further analysis classifies the proteoforms based on the nature of their variation in a semi-automated fashion. As such, different categories of proteoforms are validated following MS/MS. Comparing these classification results between datasets shows that analysis strategies tend to have an influence on the abundance of specific proteoform categories. It is thus important to keep in mind the origin and specificities of all data sources when evaluating the outcome of a proteogenomic experiment.

The different new proteoforms can also be manually and individually checked. At the MS/MS level, examples can be examined in the MaxQuant interface and on ribosome profiling level, evidence for these same examples can be loaded into a genome browser of choice using the PROTEOFORMER BedGraph files. As such, proteoforms can be studied and viewed in detail at different layers of evidence. Recently, an even more intuitive way of combining different visual information layers has become available by the definition of two proteogenomic-minded formats: proBAM and proBed (57). With these formats, results of proteomics analyses can be shown on the genomic and transcriptomic level as the proteomic identifications can now be stored in adapted SAM/BAM or BED files, widely used and able to be visualized by genome browsers. However, MaxQuant currently outputs a format which is not convertible to proBAM and proBed yet (58).

The applied approach, in which a custom search space for proteomics is obtained from analyzing matching ribosome profiling with PROTEOFORMER, enables the identification and validation of new proteoforms. At the same time, this opens opportunities for genome re-annotation and the results of this manuscript can be returned to initiatives like Ensembl (59) and UniProt (30) after manual curation (2). In that way, the feedback loop will be closed as the initially used reference information of this study can be complemented and adjusted. Further, our approach can be extended to data from other studies. All proteomics data in PRIDE (38) can be rescanned with a custom search space based on matching ribosome profiling data. Very recently, the newest update of RPFdb (60) reported that its database now contains data of 2884 ribosome profiling data sets covering 29 species. This allows to find matching ribosome profiling data for different proteomics samples in PRIDE. As such, an automated proteogenomics pipeline of PROTEOFORMER with subsequent MS/MS searching can even be set up to rescan proteomics data based on custom ribosome profiling-driven information, enabling a mass scale hunt for new proteoforms and genomic re-annotation. An example of this semi-automated setup was run on online RPFdb data of HEK293 cells (SRA project SRP014629). Analysis of matching N-terminomics MS/MS data (PRIDE dataset PXD005583) led to the identification of different classes of proteoforms, but especially uORFs and N-terminal variations, which is expected given the type of proteomic data (supplemental Fig. S10). This example demonstrates the usability of the PROTEOFORMER pipeline and its results for the broader proteomics and genomics communities.

### Proof-of-Concept Proteogenomic Experiment Using Prosit and Percolator

A first proof of concept based on Prosit (24) for including MS/MS intensities in the fragmentation spectra identification process, was issued. MaxQuant uses only the Andromeda score and a delta score (20) to rate the PSMs. As such, a lot of information gets overlooked. By using deep learning, Prosit constructs a collection of additional features for the PSMs and with Percolator (25, 26) everything is taken together in one statistical framework. It is shown that these extra features allow to filter more stringently without lowering the amount of validated PSMs. On the other hand, different strategies are applied in both Prosit (24) and Percolator (25, 26) to avoid overfitting. Further, no additional overfitting is added by combining these two tools as they function as successive steps. Overall, this first trial case shows that there is a promising advantage of adding these additional features to the PSM validation framework and that this can help the proteoform validation strategy.

The next step for this promising technique is to include the Prosit features in the protein inference algorithm (26) of Percolator to verify whether this results in extra protein identifications compared to a run with only the MaxQuant scores. These extra protein identifications can then lead to additional proteoform validations. Along with Prosit, other tools that include fragment intensities in their search algorithm like MS^2^PIP (61) and Prism (https://github.com/verilylifesciences/deepmass), can be tested in a proteogenomic context and eventually added to general proteogenomics workflows (62). Also, once these next-generation MS/MS search engines are tested, they can be encapsulated in wrappers, which allow the total PROTEOFORMER pipeline to be run. For the moment the pipeline can be run continuous up until the FASTA search space, but the MaxQuant search engine is still depending on a GUI. Clearly demonstrated by these first results (Fig. 5), we believe these MS/MS intensity-based identification strategies, all based on machine learning, are part of the way forward in proteogenomics as FDR calculation encounters challenges in this field because of the extended search space size. Because of ribosome profiling, this search space size explosion is somehow tempered compared with a 3- or 6-frame ORF translation database from RNA-seq (3, 4), but nevertheless, new approaches to lower the FDR will allow to work more stringently and validate proteogenomic outcomes with even more confidence.

## CONCLUSION

We report on a complete makeover of the PROTEOFORMER pipeline, where all newly implemented features of the pipeline drastically expand its possibilities. The combination of different proteoform calling methods optimally allows to expand the search space for MS/MS validation based on ribosome profiling. These efforts show the ability to identify a collection of MS/MS validated new proteoforms, distributed over different possible protein variant types. Moreover, a first step is taken to include MS/MS intensity-based approaches in a proteogenomics setup. Together, all these results provide novel insights for the ribosome profiling-assisted proteogenomics research.

## DATA AVAILABILITY

Raw ribosome profiling reads used in this manuscript can be found in the Gene Expression Omnibus (datasets GSE58207 and GSE74279). More details on these data can be found in the supplemental experimental protocols.

The MS/MS proteomics data have been deposited to the ProteomeXchange Consortium via the PRIDE (38) partner repository with the dataset identifier PXD011353 (http://central.proteomexchange.org/cgi/GetDataset?ID=PXD011353).

## Supplementary Material

supplemental Figs. S1, S2 and S4–S6

Supplemental materials

Table S1

Table S2

Table S3

Supplemental file S1

Supplemental file S2

Supplemental file S3

Supplemental file S4

Supplemental file S5

Supplemental file S6

Supplemental file S7

Supplemental file S8

Supplemental file S9

Supplemental file S10

Supplemental file S11

Supplemental file S12

Supplemental file S13

Supplemental file S14

Supplemental file S15

Supplemental file S16

Supplemental file S17

Supplemental file S18

Supplemental file S19

Supplemental file S20

Figures

Supplemental materials

Table S1

Table S2

Table S3

Supplemental file S1

Supplemental file S2

Supplemental file S3

Supplemental file S4

Supplemental file S5

Supplemental file S6

Supplemental file S7

Supplemental file S8

Supplemental file S9

Supplemental file S10

Supplemental file S11

Supplemental file S12

Supplemental file S13

Supplemental file S14

Supplemental file S15

Supplemental file S16

Supplemental file S17

Supplemental file S18

Supplemental file S19

Supplemental file S20

Figures

Supplemental materials

Table S1

Table S2

Table S3

Supplemental file S1

Supplemental file S2

Supplemental file S3

Supplemental file S4

Supplemental file S5

Supplemental file S6

Supplemental file S7

Supplemental file S8

Supplemental file S9

Supplemental file S10

Supplemental file S11

Supplemental file S12

Supplemental file S13

Supplemental file S14

Supplemental file S15

Supplemental file S16

Supplemental file S17

Supplemental file S18

Supplemental file S19

Supplemental file S20

## Footnotes

^1^ The abbreviations used are:

NGS: next generation sequencing
CE: collision energy
CHX: cycloheximide
FDR: false discovery rate
FPKM: fragments per kilobase million
GUI: graphical user interface
HARR: harringtonine
LTM: lactimidomycin
MS: mass spectrometry
MS/MS: tandem mass spectrometry
ORF: open reading frame
PEFF: PSI extended FASTA format
PSI: Proteomics Standards Initiative
PSM: protein-to-spectrum match
RPF: ribosome protected fragment
SAV: single amino acid variant
SNP: single nucleotide polymorphism
TIS: translation initiation site.
