# Supplementary material for: PROTEOFORMER 2.0: Further Developments in the Ribosome Profiling-assisted Proteogenomic Hunt for New Proteoforms
Source: Mol Cell Proteomics. 2019 Apr 30;18(8 Suppl 1):S126–40. doi: 10.1074/mcp.RA118.001218 (PMC6692777; doi:10.1074/mcp.RA118.001218)
Supplement: supplemental Figs. S1, S2 and S4–S6 [file RA118.001218_index.html]

Supplement to PROTEOFORMER 2.0: further developments in the ribosome profiling-assisted proteogenomic hunt for new proteoforms | Molecular & Cellular Proteomics

## Supplemental Data

- Figures - Figures of the main manuscript
- Supplemental materials - Supplemental methods and overview of the supplemental tables, figures and data
- Table S1 - Table S1
- Table S2 - Table S2
- Table S3 - Table S3
- Supplemental file S1 - Supplemental file S1
- Supplemental file S2 - Supplemental file S2
- Supplemental file S3 - Supplemental file S3
- Supplemental file S4 - Supplemental file S4
- Supplemental file S5 - Supplemental file S5
- Supplemental file S6 - Supplemental file S6
- Supplemental file S7 - Supplemental file S7
- Supplemental file S8 - Supplemental file S8
- Supplemental file S9 - Supplemental file S9
- Supplemental file S10 - Supplemental file S10
- Supplemental file S11 - Supplemental file S11
- Supplemental file S12 - Supplemental file S12
- Supplemental file S13 - Supplemental file S13
- Supplemental file S14 - Supplemental file S14
- Supplemental file S15 - Supplemental file S15
- Supplemental file S16 - Supplemental file S16
- Supplemental file S17 - Supplemental file S17
- Supplemental file S18 - Supplemental file S18
- Supplemental file S19 - Supplemental file S19
- Supplemental file S20 - Supplemental file S20
