# Supplementary material for: PROTEOFORMER 2.0: Further Developments in the Ribosome Profiling-assisted Proteogenomic Hunt for New Proteoforms
Source: Mol Cell Proteomics. 2019 Apr 30;18(8 Suppl 1):S126–40. doi: 10.1074/mcp.RA118.001218 (PMC6692777; doi:10.1074/mcp.RA118.001218)
Supplement: Supplemental materials [file 142014_2_supp_322631_psbf2t.pdf]

PROTEOFORMER 2.0: further developments in the ribosome-profiling assisted proteogenomic hunt for new proteoforms: supplemental materials

Steven Verbruggen[1,*], Elvis Ndah[1,2], Wim Van Criekinge[1], Siegfried Gessulat[3,4], Bernhard Kuster[3], Mathias Wilhelm[3], Petra Van Damme[2,5], Gerben Menschaert[1,*]

[1]BioBix, Lab of Bioinformatics and Computational Genomics, Department of Mathematical Modeling, Statistics and Bioinformatics, Faculty of Bioscience Engineering, Ghent University, Ghent, Belgium

[2]VIB-UGent Center for Medical Biotechnology, Ghent, Belgium

[3]Chair of Proteomics and Bioanalytics, Technical University of Munich, Munich, Germany

[4]SAP SE, Potsdam, Germany

[5]Department of Biochemistry and Microbiology, Faculty of Sciences, Ghent University, Ghent, Belgium

Overview of supplementary figures, tables and supplemental materials

Supplementary experimental procedures

Supplementary tables

- Supplementary Table S1: Mapping statistics of the different performed ribosome profiling data alignments.
  Format: XLSX, PDF
- Supplementary Table S2: Identification numbers obtained with MaxQuant searches.
  Format: XLSX, PDF
- Supplementary Table S3: Description of the different categories applied during proteoform classification.
  Format: XLSX, PDF

Supplementary figures

- Supplementary Figure S1: Overview of the FASTA file combinations over different proteoform calling methods (classic PROTEOFORMER proteoform calling, PRICE and SPECtre).
  Format: EPS

- Supplementary Figure S2: Overview of the merge between the combined redundant FASTA file of the three different proteoform calling methods and UniProt.

  Format: EPS
- Supplementary Figure S3: Example of the structures of the different proteoforms in human transcript ENST00000000412.

  Format: EPS
- Supplementary Figure S4: Overview of the results of mass spectrometry searches against the combined FASTA files of different proteoform calling methods.

  Format: EPS
- Supplementary Figure S5: Overview of the results of mass spectrometry searches against a merged database of the PROTEOFORMER pipeline and UniProt.

  Format: EPS
- Supplementary Figure S6: Overview of the results of mass spectrometry searches against a merged database of the PROTEOFORMER pipeline and UniProt (split up between SwissProt and TrEMBL).

  Format: EPS
- Supplementary Figure S7: Classification of the MS/MS-validated proteoforms found in PROTEOFORMER but not in the canonical UniProt database for HCT116 data.

  Format: EPS

- Supplementary Figure S8: Classification of the MS/MS-validated proteoforms found in PROTEOFORMER but not in the splicing-included UniProt database for HCT116 data.
  Format: EPS
- Supplementary Figure S9: Overview of the merging results for HEK293 data (data from online repositories).
- Supplementary Figure S10: Classification of the MS/MS-validated proteoforms found in PROTEOFORMER but not in the splicing-included UniProt database for HEK293 data (data from online repositories).

Supplementary files

- Supplementary File S1: FastQC report of HCT116 raw CHX-treated data.
  Format: PDF
- Supplementary File S2: FastQC report of HCT116 raw LTM-treated data.
  Format: PDF
- Supplementary File S3: FastQC report of Jurkat raw CHX-treated data.
  Format: PDF
- Supplementary File S4: FastQC report of Jurkat raw LTM-treated data.
  Format: PDF
- Supplementary File S5: FastQC report of HCT116 aligned CHX-treated data.
  Format: PDF
- Supplementary File S6: FastQC report of HCT116 aligned LTM-treated data.
  Format: PDF

- Supplementary File S7: FastQC report of Jurkat aligned CHX-treated data.

  Format: PDF
- Supplementary File S8: FastQC report of Jurkat aligned LTM-treated data.

  Format: PDF
- Supplementary File S9: mQC report of HCT116 aligned CHX-treated data.

  Format: PDF
- Supplementary File S10: mQC report of HCT116 aligned LTM-treated data.

  Format: PDF
- Supplementary File S11: mQC report of Jurkat aligned CHX-treated data.

  Format: PDF
- Supplementary File S12: mQC report of Jurkat aligned LTM-treated data.

  Format: PDF
- Supplementary File S13: PEFF example file for the proteoforms in the human transcript ENST00000000412.

  Format: PEFF
- Supplementary File S14: Identified protein groups and peptides of the non-redundant HCT116 methods search

  Format: XLSX
- Supplementary File S15: Identified protein groups and peptides of the redundant HCT116 methods search

  Format: XLSX

- Supplementary File S16: Identified protein groups and peptides of the redundant Jurkat methods search

  Format: XLSX
- Supplementary File S17: Identified protein groups and peptides of the search against the merge between PROTEOFORMER and canonical UniProt for HCT116

  Format: XLSX
- Supplementary File S18: Identified protein groups and peptides of the search against the merge between PROTEOFORMER and splice-included UniProt for HCT116

  Format: XLSX
- Supplementary File S19: Identified protein groups and peptides of the search against the merge between PROTEOFORMER and splice-included UniProt for Jurkat

  Format: XLSX
- Supplementary File S20: Overview of the new MS/MS validated classified proteoforms

  Format: XLSX

Supplemental experimental procedures

Ribosome profiling data

Raw sequencing reads of HCT116 ribosome profiling data (1) were downloaded from Gene Expression Omnibus (dataset GSE58207). All reads from the control (cycloheximide treated, also referred to as CHX treated, sample GSM1403307) and lactimidomycin treated sample (also referred to as LTM treated, sample GSM1403308) were used.

Raw ribosome profiling sequencing data of Jurkat cells (2) were also downloaded from Gene Expression Omnibus (dataset GSE74279). All reads from the control (CHX treated, sample GSM1916182) and LTM treated sample (sample GSM1916181) were used.

PROTEOFORMER pipeline

The PROTEOFORMER pipeline has been extensively upgraded and largely redesigned since its first publication (1). The pipeline now executes the following six important tasks: (i) quality control of the raw reads, (ii) preprocessing, prefiltering and alignment of the reads to a reference genome and pinpointing them on base level using P-site offsets, (iii) quality control of the alignments, (iv) search for transcripts with translation evidence, (v) search for translated proteoforms and (vi) export of the database to FASTA format. This FASTA file can be used as a search space for MS-based validation, possibly in combination with canonical sequences from UniProt (3). A comprehensive

visual overview of the general workflow of the updated PROTEOFORMER pipeline is given in Figure 1.

All input parameters are to be chosen by the user, so in order to provide a clear overview of all possible settings, a detailed description of the pipeline and its usage can be found in the readme file on the GitHub repository (https://github.com/Biobix/proteoformer/blob/master/README.md).

In the following subchapters, a focus on the new features of PROTEOFORMER is given. For more elaborated explanations of older features of the pipeline, we refer to the first publication of PROTEOFORMER (1). Furthermore, we give an overview of the parameters we applied during our PROTEOFORMER analysis of the Jurkat and HCT116 datasets below. When not indicated, parameters are set to the default value. The pipeline was executed on a Linux server running Fedora release 23 operation system with 64 AMD Opteron CPUs (64 cores) of 2300MHz each.

Quality control of raw reads

Before aligning the data, raw ribosome profiling sequences in FASTQ format were checked for general quality with FastQC (4), executed under the default settings suggested by the FastQC program.

Reference information

PROTEOFORMER now downloads the requested database automatically from ftp.ensembl.org. For genome annotation information, Ensembl (5) releases 86 and 92 were used respectively for human HCT116 and human Jurkat data (assembly GRCh38). Human gene annotation information in GTF format and reference sequences in FASTA

format are also downloaded automatically by PROTEOFORMER from ftp.ensemblgenomes.org. Additional reference information includes: (i) the PhiX bacteriophage genome from ftp.ncbi.nih.gov (6), (ii) human rRNA nucleotide sequences from ncbi.nlm.nih.gov/nuccore (6) (obtained using the search term 'biomol_rrna'), (iii) human sn-RNA and sn-o-RNA sequences from Biomart (7) (obtained using the gene type filters for 'sn-RNA' and 'sn-o-RNA') and (iv) human tRNA sequences from gtrnadb.ucsc.edu (8).

Sequence processing and alignment

Adaptor sequence 'AGATCGGAAGAGCACAC' was clipped and sequences were trimmed based on sequencing quality in all datasets with the FASTX toolset (0.0.14) (9). Sequences shorter than 20 nucleotides were hereby discarded and the quality threshold was set at 28. Afterwards, PROTEOFORMER filters out all contaminant PhiX, rRNA, snRNA, sn-o-RNA and tRNA sequences before mapping the remaining reads to the human genome. For all these filtering and mapping steps, the STAR (version 2.5.2a) (10) splice-aware mapping option was set. For the Jurkat data, only uniquely mapped reads were considered. In HCT116 however, this constrain was not applied and reads could map to maximum 16 different genomic positions (default parameter value) to be considered as a valid alignable sequence. The latter was chosen in order to compare unique with non-unique mapping throughout the whole process. For both Jurkat and HCT116, we ran PROTEOFORMER mapping with 'ribo' as readtype argument so that the reads from both the CHX treated and the LTM treated sample per cell line were mapped. Both a SAM and a BAM file per sample were outputted. All intermediary results of the PROTEOFORMER pipeline were stored in a central SQLite (version 3.20.1) database.

Quality control of aligned reads

For Jurkat data, it was observed that a small fraction of the aligned data was unclipped. Because this fraction gave biases in the 'Per base sequence content' plot of FastQC, sequences longer than 36 nucleotides (0,4394% of the aligned sequences) were filtered out with AWK (4.1.3).

FastQC (4) was executed a second time under default settings, but now on the aligned sequences in the SAM files.

P-site offset calculation and alignment processing

The P-site or peptidyl-site is the exact codon location where the peptidyl tRNA is formed in the ribosome, thereby unraveling the location of the translating ribosomes. The offset between the 5' site of the ribosome footprint and the P-site can be calculated for each RPF length with Plastid (11). The calculation of a correct P-site offset is important in the light of allocating an alignment to the exact base position. This also has an influence on features like triplet periodicity. Earlier versions of PROTEOFORMER could only use a fixed list of offsets based on the offset values reported in Ingolia et al. (12), but now the option is given to either calculate sample-specific P-site offsets with Plastid, to supply a user-defined list of offsets or to use a constant 3' or 5' offsets.

For this study, P-site offsets were calculated with Plastid for all samples. Then, the offsets were used to pinpoint the reads onto an exact base location, resulting in read counts per genomic base position. BED and BedGraph files were generated, allowing for visual inspection of these counts in a genome browser. Another new option of PROTEOFORMER is to produce BedGraph files for each RPF length separately. These RPF-specific files were created for all samples of this study.

mQC

Recently, mQC was developed as a tool for visualizing and exploring ribosome profiling specific features (13). FastQC can give a general overview of the data, but ribosome profiling requires extra features like triplet periodicity to be checked and visualized on a metagenomic level. In the latest version of the PROTEOFORMER pipeline, the P-site inferred reads can directly be sent to mQC for data visualization and overview report generation. The PROTEOFORMER version of mQC was run for the CHX- and LTM-setups of the HCT116 and Jurkat datasets.

Translated transcript calling

A new method for calling translated transcript isoforms, based on a zero-inflated binomial model and called RiboZinB (https://github.com/Biobix/RiboZINB), is currently being validated, but the method is already operational in the new PROTEOFORMER pipeline. As the RiboZinB model is still in alpha phase, the rule-based transcript calling was performed for all samples of this study with default parameter values as described in (1).

Proteoform calling

If mapped profiles of initiating ribosomes are available from samples treated with harringtonine (HARR) or LTM, the general proteoform calling strategy of the first PROTEOFORMER version (1) can be used (in light of this manuscript termed 'classic proteoform calling'). This calling starts with delineating the translation initiation sites (TISs) from the initiation profiles. Afterwards, single nucleotide polymorphisms (SNPs) can optionally be called. All information obtained thus far (i.e. information about

reference exon sequences, translated transcript isoforms, called TISs and SNPs) is then combined into the output amino acid sequences of so-called translated proteoforms. Details about this part of the pipeline can be consulted in the initial manuscript (1). Several upgrades were added to this method. For each proteoform, coverage and FPKM (fragments per kilobase million, i.e. the number of fragments normalized over both the sequence length in kilobase and the sequence depth in million reads) are now automatically calculated and included in the intermediary results database. Furthermore, database annotated selenocysteines (encoded by an 'UGA' codon (14), which typically serves as a STOP signal) are introduced in the translated proteoform assembly. More specifically, PROTEOFORMER extracts the list of known selenocysteines from the genes GTF file. For each selenocysteine position, both the proteoform with the selenocysteine and the one with a STOP codon instead are included in the intermediary results database.

For this study, TIS calling was executed for both datasets with default parameters. These default parameters arose from the optimization experiments done in (1). For Jurkat data, SNP calling was performed. SNP variants were extracted from the mapped CHX data with SAMtools (15) (version 1.3) and combined with variants from NCBI's dbSNP (16) (build 151). Duplicates were removed with the Picard toolkit (version 1.119). In contrast, for HCT116, SNPs were not included. For both datasets, all info was then combined into a table of all possibly translated proteoforms and their amino acid sequences constructed *in silico*.

However, if initiating ribosome profiling is not available, the approach mentioned above is not achievable. Therefore, two other options are added to PROTEOFORMER in order to allow proteoform delineation exclusively based on elongating RIBO-seq.

The first proteoform calling alternative uses 'PRICE' (17). In this technique, an RPF is seen as a result of two stochastic RNAse cleavage events. PRICE models the stochastic processes involved in ribosome profiling to capture the experimental noise and resolve the actively coding sequences. PRICE requires only a profile of the elongating ribosomes, but it can also handle the combination of both elongating and initiating ribosome profiles. A wrapper is designed to automatically run PRICE from within PROTEOFORMER. The results are then parsed into a PROTEOFORMER-formatted translated proteoform table. PRICE does not recognize selenocysteines, so this feature cannot be added for this method.

The second alternative uses 'SPECtre' (18). It is based on a classifier that rates the triplet periodicity based on spectral coherence. Again, a wrapper is devised to automatically run SPECtre from within PROTEOFORMER and use these results to construct a table of all translated proteoforms in the format and with all features required by PROTEOFORMER. Selenocysteine annotations are introduced here as well. As such, all translated proteoforms can be estimated without the need of initiating RIBO-seq data.

Both alternative proteoform calling methods (PRICE and SPECtre based) were executed in parallel with the classic method. For HCT116 data, the PRICE wrapper was run under default values. SPECtre was applied with offsets of 12 for RPF lengths 28, 29 and 30 as these were the P-site offsets calculated earlier by Plastid for the 3 most abundant RPF

lengths. The rest of the SPECtre wrapper was run with default values. For Jurkat data, the PRICE wrapper was also run under default values and in SPECtre, an offset of 12 was used for the most abundant RPF lengths 29, 30 and 31.

FASTA file export

The three methods of proteoform calling (see previous section) were stored under three different analysis IDs. For each analysis ID, sequence redundancy can be removed in the resulting amino acid sequence space, before exporting as a FASTA-formatted translation product database. Noteworthy is that both exact and substring matching is issued to remove redundant sequences. Some important changes have been applied to this functionality since its initial publication (1). When the redundancy is removed, the product IDs of the deleted redundant sequences are added to the end of the accession description of the main translation product. In that way, the IDs of these redundant open reading frames (ORFs) are still available in further MS validation steps.

For comparison, both non-redundant (mflag option 4) and redundant (mflag option 5) FASTA translation product databases of the three analysis IDs of both datasets were generated.

For Jurkat data, SNP calling was included in the analysis. This leads to SNP-specific variants of certain translation products. To handle these, an extra ID per SNP-specific variant product is added to the total product ID. A new option allows generating an additional overview text file that gives full SNP info of the translation products present as multiple SNP variants.

Database combinations

A new module was created for combining (i.e. searching what overlaps between) the FASTA files of different analyses (stored under different analysis IDs). In this combination process, only completely identical sequences found over multiple analysis strategies are taken together as one entry. Subsequences contained in a larger sequence of another analysis method, are kept as separate entities as the protein inference of an MS-based validation can favor one of the two proteoforms later on. A bin code is created and added to the accession of the combined FASTA, describing in which analysis IDs the sequence is present. So for example, bin code '101' means that the sequence is present in analysis IDs 1 and 3 and absent in analysis ID 2. Furthermore, an SQLite database is created of all entries together with their bin codes. This allows to study the results of the FASTA file combination in more detail using SQL query language.

In both datasets, the redundant as well as the non-redundant FASTA files were combined over the three different analysis IDs. Results of this combination process were also visualized in Venn diagrams.

Another new module was created in order to combine FASTA files resulting from the PROTEOFORMER pipeline with FASTA files containing annotated protein entries from UniProt (3). Similarly, only completely identical sequences are merged as one entity. For grouped IDs, the UniProt IDs are taken as the main ID in the FASTA header and the PROTEOFORMER product IDs are indicated in the descriptive part of the header. IDs of UniProt and PROTEOFORMER differ in outlook, so based on the ID, one can verify the source of the proteoform. Also, an SQLite database of this merge is created for more

detailed study with the SQL query language. Overview counts of this merging process were also visualized with Venn diagrams.

For both cell lines, the combined redundant FASTA file over the three proteoform calling methods was additionally combined with either the canonical or the splice isoform extended version of UniProt (SwissProt + TrEMBL) (release 2018_05).

PEFF format export

Recently, the HUPO Proteomics Standards Initiative (PSI) is making an effort to define an extended form of the FASTA format. It adds an additional layer of information to the FASTA file containing information about post-translational modifications, mutations and other processing events in a predefined manner so that search engines and other tools can easily process and use this information. The format was termed PEFF (PSI extended FASTA format) (http://www.psidev.info/peff). Tags are foreseen in this format to store information about SNPs and proteoform variations. As these features are available in the PROTEOFORMER pipeline, it was opportune to devise a module to export this brand-new format. The export algorithm works as follows. First, translation products are grouped in proteoform groups. Products belong to the same group if they originate from the same transcript and if they share the start and/or stop position with another member already in that group. Secondly, the representative sequence (i.e. base sequence) for the group is determined. Features of a representative base sequence are listed in descending importance: (i) translation initiation at a canonical TIS, (ii) being the longest sequence of the group, (iii) having the least amount of SNP variants in comparison to the reference genome. Thirdly, every proteoform is aligned to the base representative with Clustal Omega (19) and this alignment is parsed into the different

needed SNP and proteoform tags per group member. Finally, for each group, an entry is constructed and written to the output PEFF file.

PROTEOFORMER implementation

All information on the available scripts, the usage and the different implementations of PROTEOFORMER is available on the GitHub repository (https://github.com/Biobix/proteoformer). The README.md file in this directory gives a comprehensive overview of the pipeline usage. The examples_commands.md file gives an overview of the commands we used to generate our results for HCT116 and Jurkat.

PROTEOFORMER is available as a script-based tool (Perl 5 and Python 2.7). To make it available for non-experts, a Galaxy (20) implementation is available for download. Necessary files can be easily downloaded from GitHub to a Unix system and added to a Galaxy instance. For easy dependency management in both the script and Galaxy version, all dependencies were added to a downloadable Conda (21) environment. We host a public Galaxy version of PROTEOFORMER at galaxy.ugent.be.

Matching mass spectrometry data

Cell culture

The human HCT116 colon cancer cell line was kindly provided to us by the Johns Hopkins Sidney Kimmel Comprehensive Cancer Center (Baltimore, USA) and cultivated in McCoy's medium (Gibco™, cat nº 22330-021). Human Jurkat T-lymphocytes (clone E6-1; ATCC, ATCC® TIB-152™) were cultured in Roswell Park Memorial Institute (RPMI)-1640 medium (Gibco™, cat nº 16870-010). All media contained 2 mM alanyl-L-

glutamine dipeptide (GlutaMAX) and were supplemented with 10 % fetal bovine serum (E.U.-approved, South American origin (Gibco™, cat nº 10270106), 50 units/ml penicillin (Gibco™, cat nº 15070-063) and 50 µg/ml streptomycin (Gibco™, cat nº 15070-063)). Cells were cultured at 37°C in a humidified atmosphere with 5% CO_2 and passaged every 3-4 days.

Proteome sample preparation for shotgun analyses

Cell pellets of HCT116 and Jurkat cells -obtained from 4 independent cell cultures and containing approx. 10 x 10^6 cells per pellet- were collected and stored at -80°C until further processing. Cell pellets were resuspended in 1 ml Gu.HCl lysis buffer (4 M Gu.HCl, 50 mM NH_4HCO_3 (pH 7.9)) and subjected to three rounds of freeze-thaw lysis in liquid N_2. The lysates were sonicated (Branson probe sonifier output 4, 50% duty cycle, 3×30 s, 1 sec pulses) followed by centrifugation for 10 min at 16,100 g (4 °C), the supernatant removed and protein concentration determined by Bradford measurement according to the manufacturer's instructions. An aliquot equivalent of 400 µg (~2x10^6 cells) was transferred to a clean tube, diluted to 2 mg/ml with lysis buffer, 2x diluted with HPLC grade water, and precipitated with 4× volumes of −20 °C acetone overnight. The precipitated protein material was recovered by centrifugation for 15 min at 3,500g (4 °C), pellets washed twice with −20 °C 80% acetone, and air dried upside down for ~10 min at RT or until no residual acetone odor remained. Pellets were resuspended in 200 µl TFE (2,2,2-trifluoroethanol) digestion buffer (10% TFE, 100 mM NH_4HCO_3 (pH 7.9)) with sonication until a homogenous suspension was reached. All samples were digested overnight at 37°C using mass spec grade trypsin (Promega, Madison, WI) (enzyme/substrate of 1:50 w/w) while mixing (550 rpm). Samples were acidified with

TFA to a final concentration of 0.5%. Samples were cleared from insoluble particulates by centrifugation for 10 min at 16,100 g (4°C) and the supernatant transferred to clean tubes. Methionine oxidation was performed by the addition of H_2O_2 to reach a f.c. of 0.5% for 30' at 30°C. Solid phase extraction of peptides was performed using C18 reversed phase sorbent containing 100 µl pipette tips (Bond Elut OMIX 100 µl C18 tips (Agilent, Santa Clara, CA, USA)) according to the manufacturer's instructions. The pipette tip was conditioned by aspirating the maximum pipette tip volume of water:acetonitrile, 50:50 (v/v) and the solvent discarded. After equilibration of the tip by washing 3 times with the maximum pipette tip volume in 0.1% TFA in water, 100 µl of the acidified samples (~200 µg) were dispensed and aspirated for 10 cycles for maximum binding efficiency. The tip was washed 3 times with the maximum pipette tip volume of 0.1% TFA in water:acetonitrile, 98:2 (v/v) and the bound peptides eluted in LC-MS/MS vials with the maximum pipette tip volume of 0.1% TFA in water:acetonitrile, 30:70 (v/v). The samples were vacuum-dried in a SpeedVac concentrator and re-dissolved in 20 µL of 2 mM tris(2-carboxyethyl)phosphine in 2% acetonitrile.

LC-MS/MS analysis (Q-Exactive HF instrument)

Samples were analyzed by LC-MS/MS using an UltiMate 3000 RSLC nano HPLC (Dionex) in-line connected to a Q-Exactive HF mass spectrometer (Thermo Fisher Scientific Inc.). Samples were separated on a 40 cm column packed in the needle (produced in-house, 75 µm I.D. × 400 mm, 1.9 µm beads C18 Reprosil-HD, Dr. Maisch) using a non-linear 150 min gradient of 2-56% solvent B' (0.1% formic acid (FA) in water/ACN, 20/80 (v/v)) at a flow rate of 250 nL/min. This was followed by a 10 min wash reaching 99% solvent B'

and re-equilibration with solvent A (0.1% FA in water). Column temperature was kept constant at 50°C (CoControl 3.3.05, Sonation). The mass spectrometer was operated in data-dependent, positive ionization mode, automatically switching between MS and MS/MS acquisition for the 16 most abundant peaks in a given MS spectrum. The source voltage was set to 3.5 kV and the capillary temperature was 250°C. One MS1 scan (m/z 375-1500, AGC target 3E6 ions, maximum ion injection time of 45 ms) acquired at a resolution of 60,000 (at 200 m/z) was followed by up to 16 tandem MS scans (resolution 15,000 at 200 m/z) of the most intense ions fulfilling predefined selection criteria (AGC target 1E5 ions, maximum ion injection time of 60 ms, isolation window of 1.5 m/z, fixed first mass of 145 m/z, spectrum data type: centroid, under fill ratio 2%, intensity threshold 1.3E4, exclusion of unassigned, singly charged precursors, peptide match preferred, exclude isotopes on, dynamic exclusion time of 12 s). The HCD collision energy was set to 32% Normalized Collision Energy and the polydimethylcyclosiloxane background ion at 445.12002 Da was used for internal calibration (lock mass).

Mass spectrometry data analysis

Experimental design and statistical rationale

The general rationale was to expand the known canonical sequence database with extra sequences from the PROTEOFORMER pipeline in advance. Matching proteomics data was then searched against these extended search space using MaxQuant (22) (http://www.coxdocs.org/doku.php?id=maxquant:start). The protein inference strategy of the search program converts peptide counts into proteins. In that way, after

running MaxQuant, protein groups in which a new variation scored more peptide counts compared to a canonical product can easily be examined. If this higher count can be explained by a peptide spanning the distinctive region of the sequence (e.g. a C-terminal extension, an N-terminus of an N-truncated protein, a peptide covering a single amino acid variation (SAV)…), this validates the existence of the newly identified variant.

Mass spectrometry assisted validation was performed analyzing the proteomes of matching HCT116 and Jurkat samples. For both samples, the 4 biological replicates were analyzed. In HCT116, 315 470 MS/MS scans were sampled in total, for Jurkat, 311 100.

Mass spectrometry analysis with MaxQuant

The raw MS/MS files were searched using the different combined PROTEOFORMER and UniProt databases, described earlier. For database searching, the MaxQuant GUI (version 1.6.1.0) was used. It uses the Andromeda search engine to directly parse and query the raw data files. The 4 replicates of each sample were analyzed and combined within a single MaxQuant run. Trypsin was selected as cleavage enzyme and was allowed even if a proline is following the basic residues arginine or lysine. Two missed cleavages were allowed. Methionine oxidation was selected as a fixed and N-terminal acetylation as a variable modification. Precursor and fragment ion mass tolerance were set to 20 ppm. Contaminants from the built-in MaxQuant contaminant database were omitted in the results. Minimum Andromeda score for an MS/MS identification to pass, was 0 for unmodified and 40 for modified peptides. The minimum Andromeda delta score for unmodified and modified peptides was respectively set to 0 and 6. Matching

between runs was allowed. The LFQ algorithm was applied for label-free quantification and the iBAQ method was used for calculating protein abundances. Only unique and no razor peptides were selected for protein quantification. Both the PSM (peptide-to-spectrum match), XPSM and protein FDR threshold were set at 0,01.

MaxQuant results parsing

MaxQuant results of identified protein groups, peptides and PSMs were parsed for counting the share of each proteoform calling method in the total MS output. For PSMs and peptides, the bin codes of all proteins in which a particular PSM or peptide is present, were taken together to obtain the range of methods in which that PSM or peptide was found. Applying this approach for all PSMs or peptides gives a count +1 to that obtained range of methods. For protein groups, a slightly different strategy was applied. For each protein group, the proteins with the maximum peptide count of the group are considered as identified. For example, if two proteins differ by a variation (e.g. an N-terminal extension), then the two proteins are seen as identified if they hold the same number of identified peptides because there is no distinguishing peptide to give one of the two an advantage over the other. On the other hand, if one of the two has a higher number of identifications because of peptides specific to the variation (e.g. a peptide in the N-terminal extension or a peptide overlapping the canonical start as depicted in Figure 4), then these extra peptides confirm the presence of the variation. As such, this proteoform can be classified as identified. In this way, the proteins with the maximum peptide count are extracted and the bin codes of their accessions are taken together to obtain the range of methods in which the proteins were found per protein group. This range of methods gets then a count +1. Counts per proteoform

calling method were plotted in Venn diagrams to clearly show the overlap regions. Area sizes of the Venn diagram circles are sized in relation to the log(x+1) transformation of the counts as this gave the best visual representation for MS/MS identification counts. For studying more features, identification results at the protein level can be queried using an SQLite database and SQL query language.

A similar approach was used for counting the identifications explained by the combined PROTEOFORMER pipeline versus UniProt. As such, results of MaxQuant were converted to counts attributable to PROTEOFORMER, UniProt or both on the level of PSMs, peptides and protein groups. Counts were presented as Venn diagrams, allowing to visualize the overlap between the PROTEOFORMER pipeline and UniProt. Area sizes of the Venn diagram circles are also log(x+1) transformed. Protein counting results were also saved in an SQLite database.

MS/MS validated proteins, specifically added to the search space by the PROTEOFORMER pipeline, were further investigated as this collection of proteins contains validated proteoforms that are not explained by reference information from UniProt. Each proteoform in this collection was compared to its closest UniProt relative belonging to the same protein group during the MS/MS search. If no closest UniProt relative was present in the protein group, the canonical translation product of the transcript was fetched from the reference annotation. This comparison reveals for each proteoform where it differs from the canonical reference and thus pinpoints the actual variation point (e.g. a truncation, an extension, a splice isoform change, a SAV). The proteoform can also be translated from a supposed untranslated 5' header or 3' trailer region of the transcript or can originate from so-called non-coding transcripts. For

SAV's, it is important to understand that the identified SAV's will be relative to what is taken as reference in UniProt. Next, it was checked if one or more peptides could be found that specifically confirm the presence of the variation. The proteoform was classified as a true new MS/MS-validated proteoform and based on the nature of the variation, proteoforms were classified and counted in different proteoform groups. These counts are represented in pie charts.

Expanded MS/MS features calculation with Prosit

We extracted up to 15 top-ranking candidate PSMs for all scans in each RAW file from the Andromeda generated res-files as input for fragment intensity prediction with Prosit (available at www.proteomicsdb.org/prosit/). CE (collsion energy) 35 was estimated as optimal CE for prediction by Prosit's CE alignment on a high-scoring subset of the data. After prediction, Prosit generated two input files for Percolator both containing all standard Percolator features. The first file, serving as benchmark, adds features from MaxQuant: Andromeda score and Andromeda delta score. The second file adds 52 features mostly based on Prosit's fragment intensity prediction. An example for such a feature is the number of observed fragment ions, that also were predicted for a given PSM. This approach is similar to the analysis of metaproteomics data described by Gessulat et al. (23).

Analysis of expanded MS features with Percolator

Percolator (version 3.02.1) (24, 25) was used to combine the scores and features of MaxQuant with the new features obtained from the Prosit algorithm. As a proof of concept, this strategy was examined for the MS/MS search results against the combined

redundant PROTEOFORMER search space of HCT116 merged with the canonical UniProt database. As a reference, Percolator was first run on the MaxQuant features only (Andromeda score and delta score). Later, q values of PSMs from this reference analysis were compared with q values obtained after inclusion of all features calculated by Prosit. For these Percolator runs, the mix-max method was used for obtaining q values. Furthermore, the option to keep all PSMs was selected. All other parameters were set to the default values.

Illustrative example study of a semi-automated proteogenomics pipeline based on online repository data of HEK293 cells

In order to demonstrate the use of the proposed semi-automated pipeline to identify new proteoforms based on online repository data from RPFdb and PRIDE, we sat up a test case. Results of this test case can be found in Supplementary Figures S9-10 and in Supplementary File S20D.

Data origin

Ribosome profiling data in human HEK293 cells from study 'SRP014629' (26) on NCBI SRA were used. In particular, datasets 'SRR618770' and 'SRR618771' were combined as CHX-treated raw data sample. 'SRR618772' and 'SRR618773' were combined as LTM-treated raw data sample. The MS/MS data (sample: Amicon, Tryp, replicate 1) for this search was obtained from PRIDE dataset PXD005583, an N-terminomics MS/MS study in HEK293T cells.

Data analysis

Ribosome profiling data was mainly analyzed analogue to the HCT116 and Jurkat experiments, except of following adaptations: mapping was done non-uniquely, a read length of 48nt was selected and a poly-A adapter of 48nt long was clipped off. Proteoform candidates of the classic proteoform calling, PRICE and SPECtre were combined with the splice-aware version of UniProt to build a custom search space, which was then searched with MaxQuant against the downloaded MS/MS data. MaxQuant searches were done with the parameters suggested in the paper of the MS/MS data source (27). Results of these MaxQuant searches were processed with the additional PROTEOFORMER scripts, already used for HCT116 and Jurkat.

References

1. Crappé, J., Ndah, E., Koch, A., Steyaert, S., Gawron, D., De Keulenaer, S., De Meester, E., De Meyer, T., Van Criekinge, W., Van Damme, P., and Menschaert, G. (2014) PROTEOFORMER: deep proteome coverage through ribosome profiling and MS integration. *Nucleic Acids Res.* 43, e29
2. Gawron, D., Ndah, E., Gevaert, K., and Van Damme, P. (2016) Positional proteomics reveals differences in N-terminal proteoform stability. *Mol. Syst. Biol.* 12, 858–858
3. The UniProt Consortium (2017) UniProt: The universal protein knowledgebase. *Nucleic Acids Res.* 45, D158–D169
4. Andrews, S. (2010) FastQC: A quality control tool for high throughput sequence data. *unpublished*,

5. Zerbino, D. R., Achuthan, P., Akanni, W., Amode, M. R., Barrell, D., Bhai, J., Billis, K., Cummins, C., Gall, A., Girón, C. G., Gil, L., Gordon, L., Haggerty, L., Haskell, E., Hourlier, T., Izuogu, O. G., Janacek, S. H., Juettemann, T., To, J. K., Laird, M. R., Lavidas, I., Liu, Z., Loveland, J. E., Maurel, T., McLaren, W., Moore, B., Mudge, J., Murphy, D. N., Newman, V., Nuhn, M., Ogeh, D., Ong, C. K., Parker, A., Patricio, M., Riat, H. S., Schuilenburg, H., Sheppard, D., Sparrow, H., Taylor, K., Thormann, A., Vullo, A., Walts, B., Zadissa, A., Frankish, A., Hunt, S. E., Kostadima, M., Langridge, N., Martin, F. J., Muffato, M., Perry, E., Ruffier, M., Staines, D. M., Trevanion, S. J., Aken, B. L., Cunningham, F., Yates, A., and Flicek, P. (2018) Ensembl 2018. *Nucleic Acids Res.* 46, D754–D761

6. Geer, L. Y., Marchler-Bauer, A., Geer, R. C., Han, L., He, J., He, S., Liu, C., Shi, W., and Bryant, S. H. (2010) The NCBI BioSystems database. *Nucleic Acids Res.* 38, D492–D496

7. Smedley, D., Haider, S., Durinck, S., Pandini, L., Provero, P., Allen, J., Arnaiz, O., Awedh, M. H., Baldock, R., Barbiera, G., Bardou, P., Beck, T., Blake, A., Bonierbale, M., Brookes, A. J., Bucci, G., Buetti, I., Burge, S., Cabau, C., Carlson, J. W., Chelala, C., Chrysostomou, C., Cittaro, D., Collin, O., Cordova, R., Cutts, R. J., Dassi, E., Di Genova, A., Djari, A., Esposito, A., Estrella, H., Eyras, E., Fernandez-Banet, J., Forbes, S., Free, R. C., Fujisawa, T., Gadaleta, E., Garcia-Manteiga, J. M., Goodstein, D., Gray, K., Guerra-Assunção, J. A., Haggarty, B., Han, D. J., Han, B. W., Harris, T., Harshbarger, J., Hastings, R. K., Hayes, R. D., Hoede, C., Hu, S., Hu, Z. L., Hutchins, L., Kan, Z., Kawaji, H., Keliet, A., Kerhornou, A., Kim, S., Kinsella, R., Klopp, C., Kong, L., Lawson, D., Lazarevic, D., Lee, J. H., Letellier, T., Li, C. Y., Lio, P., Liu, C. J., Luo, J.,

Maass, A., Mariette, J., Maurel, T., Merella, S., Mohamed, A. M., Moreews, F., Nabihoudine, I., Ndegwa, N., Noirot, C., Perez-Llamas, C., Primig, M., Quattrone, A., Quesneville, H., Rambaldi, D., Reecy, J., Riba, M., Rosanoff, S., Saddiq, A. A., Salas, E., Sallou, O., Shepherd, R., Simon, R., Sperling, L., Spooner, W., Staines, D. M., Steinbach, D., Stone, K., Stupka, E., Teague, J. W., Dayem Ullah, A. Z., Wang, J., Ware, D., Wong-Erasmus, M., Youens-Clark, K., Zadissa, A., Zhang, S. J., and Kasprzyk, A. (2015) The BioMart community portal: An innovative alternative to large, centralized data repositories. *Nucleic Acids Res.* 43, W589–W598

8. Chan, P. P., and Lowe, T. M. (2016) GtRNAdb 2.0: An expanded database of transfer RNA genes identified in complete and draft genomes. *Nucleic Acids Res.* 44, D184–D189

9. Gordon, A., and Hannon, G. J. (2010) Fastx-toolkit. *unpublished*,

10. Dobin, A., Davis, C. A., Schlesinger, F., Drenkow, J., Zaleski, C., Jha, S., Batut, P., Chaisson, M., and Gingeras, T. R. (2013) STAR: Ultrafast universal RNA-seq aligner. *Bioinformatics* 29, 15–21

11. Dunn, J. G., and Weissman, J. S. (2016) Plastid: nucleotide-resolution analysis of next-generation sequencing and genomics data. *BMC Genomics* 17, 958

12. Ingolia, N. T., Lareau, L. F., and Weissman, J. S. (2011) Ribosome profiling of mouse embryonic stem cells reveals the complexity and dynamics of mammalian proteomes. *Cell* 147, 789–802

13. Verbruggen, S., and Menschaert, G. (2018) mQC: a post-mapping data exploration tool for ribosome profiling. *Comput. Methods Programs Biomed.*, in press

14. Böck, A., Forchhammer, K., Heider, J., and Baron, C. (1991) Selenoprotein

synthesis: an expansion of the genetic code. *Trends Biochem. Sci.* 16, 463–467

15. Li, H., Handsaker, B., Wysoker, A., Fennell, T., Ruan, J., Homer, N., Marth, G., Abecasis, G., and Durbin, R. (2009) The Sequence Alignment/Map format and SAMtools. *Bioinformatics* 25, 2078–2079

16. Sherry, S. T., Ward, M.-H., Kholodov, M., Baker, J., Phan, L., Smigielski, E. M., and Sirotkin, K. (2001) dbSNP: the NCBI database of genetic variation. *Nucleic Acids Res.* 29, 308–311

17. Erhard, F., Halenius, A., Zimmermann, C., Lhernault, A., Kowalewski, D., Weekes, M. P., Stevanovic, S., Zimmer, R., and Lars, D. (2018) Improved Ribo-seq enables accurate and validated identification of cryptic translation events. *Nat. Methods* 15, 363–366

18. Chun, S. Y., Rodriguez, C. M., Todd, P. K., and Mills, R. E. (2016) SPECtre: a spectral coherence--based classifier of actively translated transcripts from ribosome profiling sequence data. *BMC Bioinformatics* 17, 482

19. Sievers, F., Wilm, A., Dineen, D., Gibson, T. J., Karplus, K., Li, W., Lopez, R., McWilliam, H., Remmert, M., Söding, J., Thompson, J. D., and Higgins, D. G. (2011) Fast, scalable generation of high-quality protein multiple sequence alignments using Clustal Omega. *Mol. Syst. Biol.* 7, 539

20. Goecks, J., Nekrutenko, A., and Taylor, J. (2010) Galaxy: a comprehensive approach for supporting accessible, reproducible, and transparent computational research in the life sciences. *Genome Biol.* 11, R86

21. Grüning, B., Dale, R., Sjödin, A., Chapman, B. A., Rowe, J., Tomkins-Tinch, C. H., Valieris, R., and Köster, J. (2018) Bioconda: Sustainable and comprehensive

software distribution for the life sciences. *Nat. Methods* 15, 475–476

22. Cox, J., and Mann, M. (2008) MaxQuant enables high peptide identification rates, individualized p.p.b.-range mass accuracies and proteome-wide protein quantification. *Nat. Biotechnol.* 26, 1367–1373

23. Gessulat, S., Schmidt, T., Zolg, D. P., Samaras, P., Schnatbaum, K., Zerweck, J., Knaute, T., Rechenberger, J., Delanghe, B., Huhmer, A., Reimer, U., Ehrlich, H., Aiche, S., Kuster, B., and Wilhelm, M. (2019) Prosit: proteome-wide prediction of peptide tandem mass spectra by deep learning. *Nat. Methods* 16, 509–518

24. Käll, L., Canterbury, J. D., Weston, J., Noble, W. S., and MacCoss, M. J. (2007) Semi-supervised learning for peptide identification from shotgun proteomics datasets. *Nat. Methods* 4, 923–925

25. The, M., MacCoss, M. J., Noble, W. S., and Käll, L. (2016) Fast and Accurate Protein False Discovery Rates on Large-Scale Proteomics Data Sets with Percolator 3.0. *J. Am. Soc. Mass Spectrom.* 27, 1719–1727

26. Lee, S., Liu, B., Lee, S., Huang, S.-X., Shen, B., and Qian, S.-B. (2012) Global mapping of translation initiation sites in mammalian cells at single-nucleotide resolution. *Proc. Natl. Acad. Sci.* 109, E2424–E2432

27. Yeom, J., Ju, S., Choi, Y., Paek, E., and Lee, C. (2017) Comprehensive analysis of human protein N-termini enables assessment of various protein forms. *Sci. Rep.* 7, 6599

Supplementary tables

Supplementary Table S1: Mapping statistics of the different performed ribosome profiling data alignments. For each treatment of both samples, data were first filtered against bacteriophage PhiX, rRNA, snRNA (and sn-o-RNA) and tRNA. The filtered data was then genomically aligned to the human reference genome, with an extra division for unique and multi-mapped reads. For Jurkat data, only unique alignment was applied. A clear difference in rRNA content between both samples is observed. Jurkat data is around 2-3 times more covered than HCT116 on the raw read level, but this reaches to around 3-4 times more coverage for Jurkat on the genomic alignment level.

Sample	Treatment	Type	Total reads	Unique mapped	Multimapped	Total mapped	Unmapped	Unique mapped freq.	Multimapped freq.	Total mapped freq.
HCT116	CHX	PhiX	142 802 165			358 516	142 443 649			0,25%
		rRNA	142 443 649			105 649 115	36 794 534			74,17%
		snRNA	36 794 534			1 227 391	35 567 143			3,34%
		tRNA	35 567 143			4 434 117	31 133 026			12,47%
		Genomic	31 133 026	17 606 923	8 728 708	26 335 631	4 797 395	56,55%	28,04%	84,59%
	LTM	PhiX	151 696 882			395 021	151 301 861			0,26%
		rRNA	151 301 861			99 922 436	51 379 425			66,04%
		snRNA	51 379 425			2 119 809	49 259 616			4,13%
		tRNA	49 259 616			4 063 557	45 196 059			8,25%
		Genomic	45 196 059	25 692 092	11 403 645	37 095 737	8 100 322	56,85%	25,23%	82,08%
Jurkat	CHX	PhiX	361 801 191			2 826 450	358 974 741			0,78%
		rRNA	358 974 741			141 995 459	216 979 282			39,56%
		snRNA	216 979 282			25 914 185	191 065 097			11,94%
		tRNA	191 065 097			4 691 636	186 373 461			2,46%
		Genomic	186 373 461	124 995 073	0	124 995 073	61 378 388	67,07%	0,00%	67,07%
	LTM	PhiX	303 819 671			2 794 788	301 024 883			0,92%
		rRNA	301 024 883			119 058 484	181 966 399			39,55%
		snRNA	181 966 399			27 196 696	154 769 703			14,95%
		tRNA	154 769 703			13 357 840	141 411 863			8,63%
		Genomic	141 411 863	97 466 121	0	97 466 121	43 945 742	68,92%	0,00%	68,92%

Supplementary Table S2: Identification numbers obtained with MaxQuant searches. MS data analysis was done in HCT116 and Jurkat samples. In order to compare ribosome profiling proteoform calling methods, search spaces were constructed in both samples. The size of this FASTA search spaces on ribosome profiling level is given in the table. For HCT116, options with and without redundancy removal were tried. Afterwards, redundant versions were merged with UniProt, which was added in both canonical as splice variant included version for HCT116. All these options of ribosome profiling-assisted search space construction were used in validation on matching MS data with MaxQuant. The amount of identifications on the level of protein groups, peptides and peptide-to-spectrum matches (PSMs) is given in the table. As a reference, the last row gives the identification numbers of a MaxQuant search in HCT116 against only the canonical UniProt database.

Sample	Redundancy	UniProt	Sequences in search space	Identified protein groups	Identified peptides	Identified PSMs
HCT116	No	/	92 931	4 322	28 729	179 175
	Yes	/	133 051	4 330	28 684	179 056
Jurkat	Yes	/	201 419	4 450	28 689	172 273
HCT116	Yes	Canonical	176 202	4 333	28 402	177 473
		Spliced	186 627	4 347	28 372	176 978
Jurkat	Yes	Spliced	253 734	4 477	28 548	171 116
HCT116, only UniProt		Canonical	71 356	4 294	28 443	180 526

Supplementary Table S3: Description of the different categories applied during proteoform classification.

Proteoform class	Description
Splice variants	Proteoforms with exon variations compared to the canonical form.
Exon inclusion	The proteoform contains additional internal exons.
Exon exclusion	The proteoform lacks one or more internal exons.
Exon substitution	An exon of the canonical protein has been replaced for another exon.
C-terminal splice variant	A C-terminal exon or exon part has been replaced.
N-terminal splice variant	An N-terminal exon or exon part has been replaced.
Translation in non-coding region	Translated proteoforms in formerly considered untranslated transcripts. Subcategories based on Ensembl biotypes.
Processed transcript	Transcripts that do not contain a known ORF, like lncRNAs and ncRNAs.
Processed pseudogene	Pseudogenes are similar to known proteins but they contain a frameshift and/or stop codon that disrupts the ORF. Processed pseudogenes lack introns and are thought to arise from reverse mRNA transcription and DNA reinsertion.
Transcribed processed pseudogene	Protein homology or genomic structure indicates that it is a pseudogene but the presence of locus-specific transcripts indicates expression.
Retained intron	Proteoform translated from a transcript that has intronic sequences compared to other coding transcripts.
C-terminal extension	Proteoform with extra sequence information added to the C-terminus.
Multiple variations	Proteoform with a combination of variants from the other categories, making it difficult to place it in one categorie. Manual inspection can lead to classification.
N-terminal extension	Proteoform with extra sequence information added to the N-terminus. Translation starts in the earlier considered 5' untranslated region and continues over the canonical initiation site.
N-terminal truncation	Proteoform lacking a sequence part at the N-terminus.
Only amino acid substitutions	Proteoform differing from the canonical form only by single amino acid variations.
Out of frame ORF	Translation product contained in frame +1 or +2 of a known ORF.
dORF	Translation product originating from the 3' untranslated region of a known protein-coding transcript.
uORF	Translation product originating from the 5' untranslated region of a known protein-coding transcript.

Supplementary figures

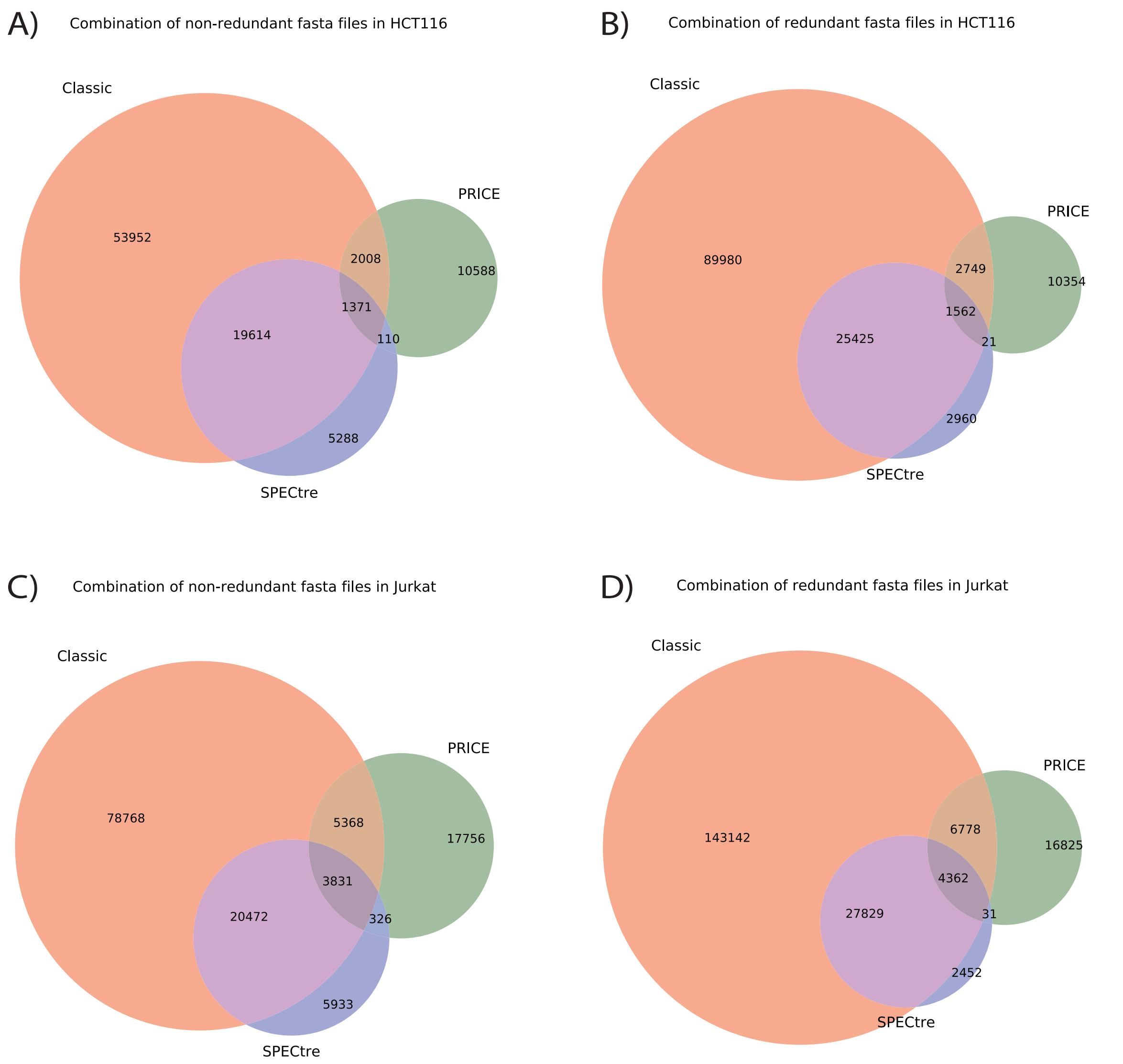

Supplementary Figure S1: Overview of the FASTA file combinations over different proteoform calling methods (classic PROTEOFORMER proteoform calling, PRICE and SPECtre). The Venn diagrams show how much overlap there is between the different methods. (A) Combination of non-redundant (redundancy removed during method-

specific database export) FASTA files for HCT116 data. (B) Combination of redundant FASTA files for HCT116 data. (C) Combination of non-redundant FASTA files for Jurkat data. (D) Combination of redundant FASTA files for Jurkat data.

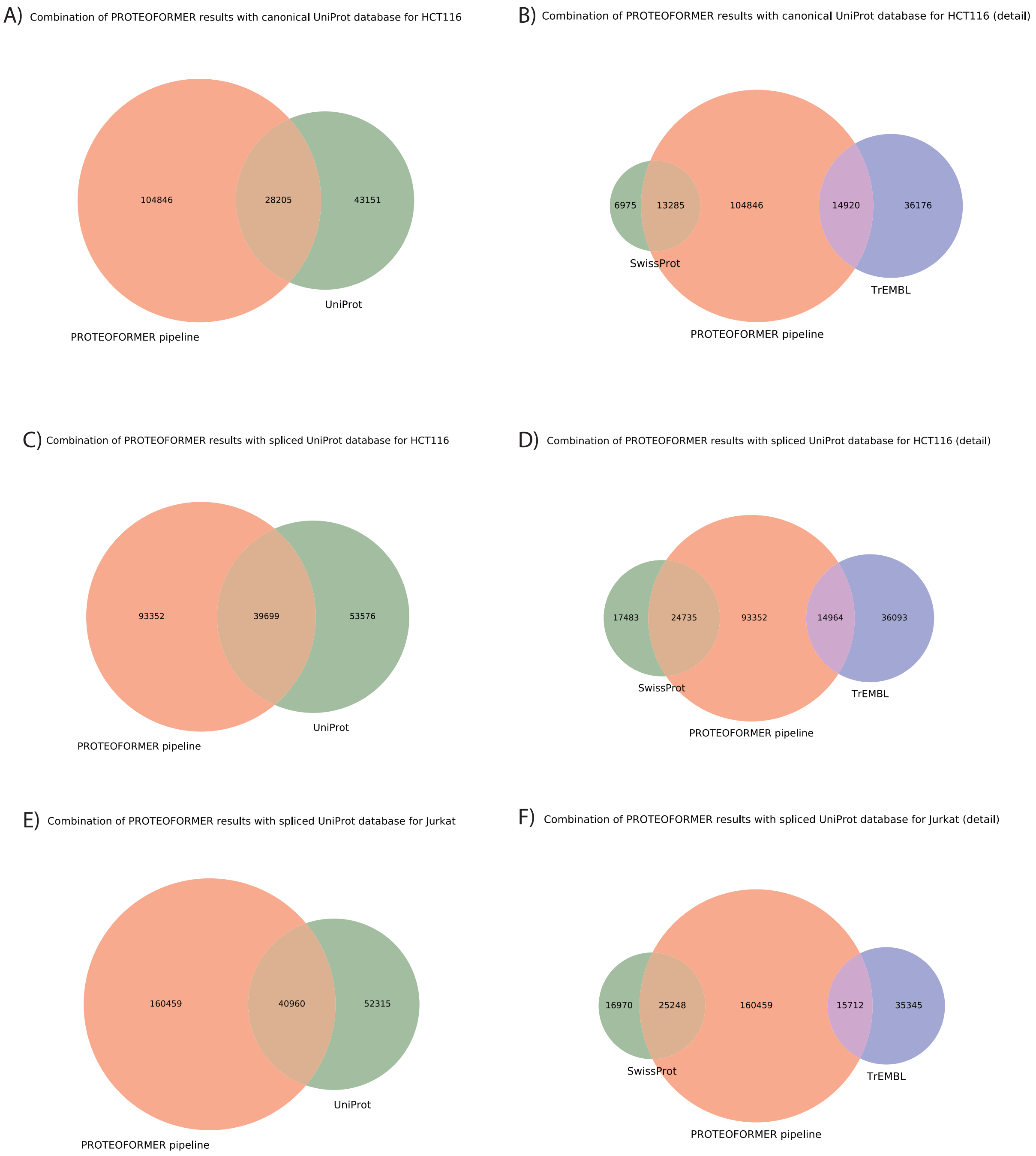

Supplementary Figure S2: Overview of the merge between the combined redundant FASTA file of the three different proteoform calling methods and UniProt. The Venn diagrams show the overlap between the results of the combined PROTEOFORMER pipeline and the

sequences in UniProt (consisting of SwissProt manual annotated info and TrEMBL). (A) Overlap between PROTEOFORMER and the canonical info from UniProt for HCT116 data. (B) Overlap of PROTEOFORMER with the canonical versions of SwissProt and TrEMBL for HCT116 data. (C) Overlap between PROTEOFORMER and UniProt with splice isoforms included for HCT116 data. (D) Overlap of PROTEOFORMER with the splice isoform included versions of SwissProt and TrEMBL for HCT116 data. (E) Overlap between PROTEOFORMER and UniProt with splice isoforms included for Jurkat data. (G) Overlap of PROTEOFORMER with the splice isoform included versions of SwissProt and TrEMBL for Jurkat data.

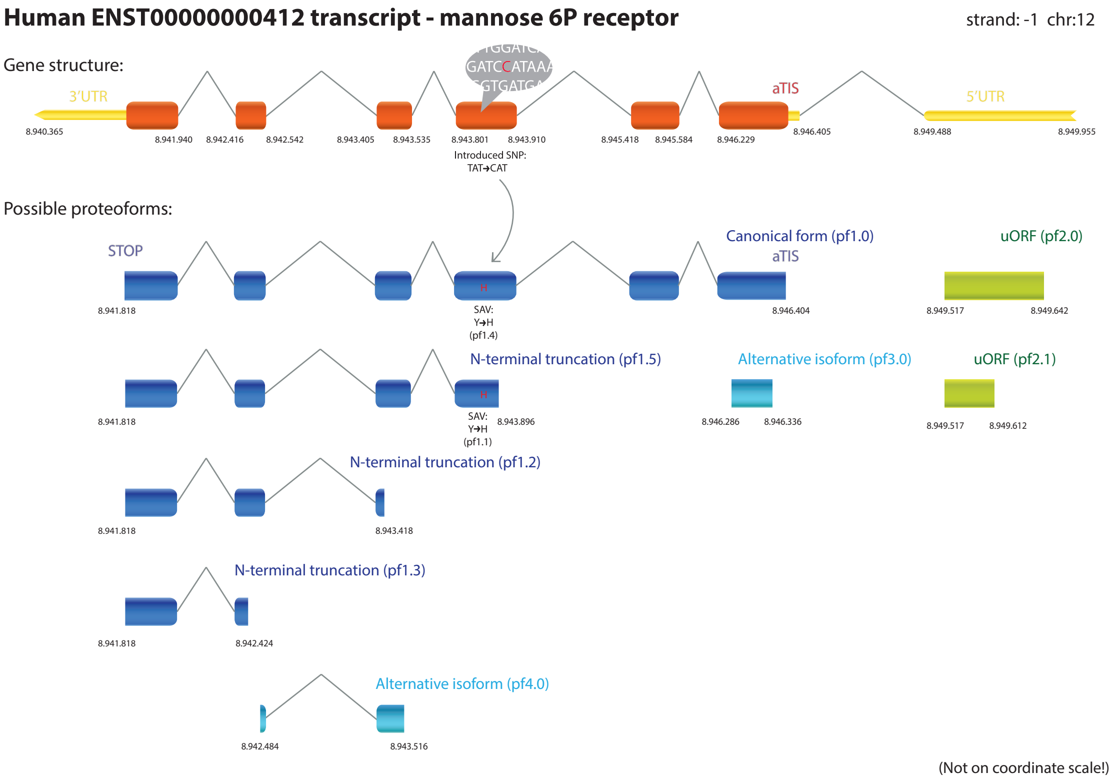

Supplementary Figure S3: Example of the structures of the different proteoforms in human transcript ENST00000000412 (antisense). These different proteoforms are included in the example of the PEFF file in Supplementary File S13. Proteoform 1.0 up to proteoform 1.5 comprise the canonical translation product with its variations due to N-terminal truncations and SNPs (leading to single amino acid variations). Proteoform 2.0 and 2.1 describe 2 linked uORFs of the canonical product, differing by an N-terminal truncation. Proteoform 3.0 and 4.0 are 2 alternative reading frame products.

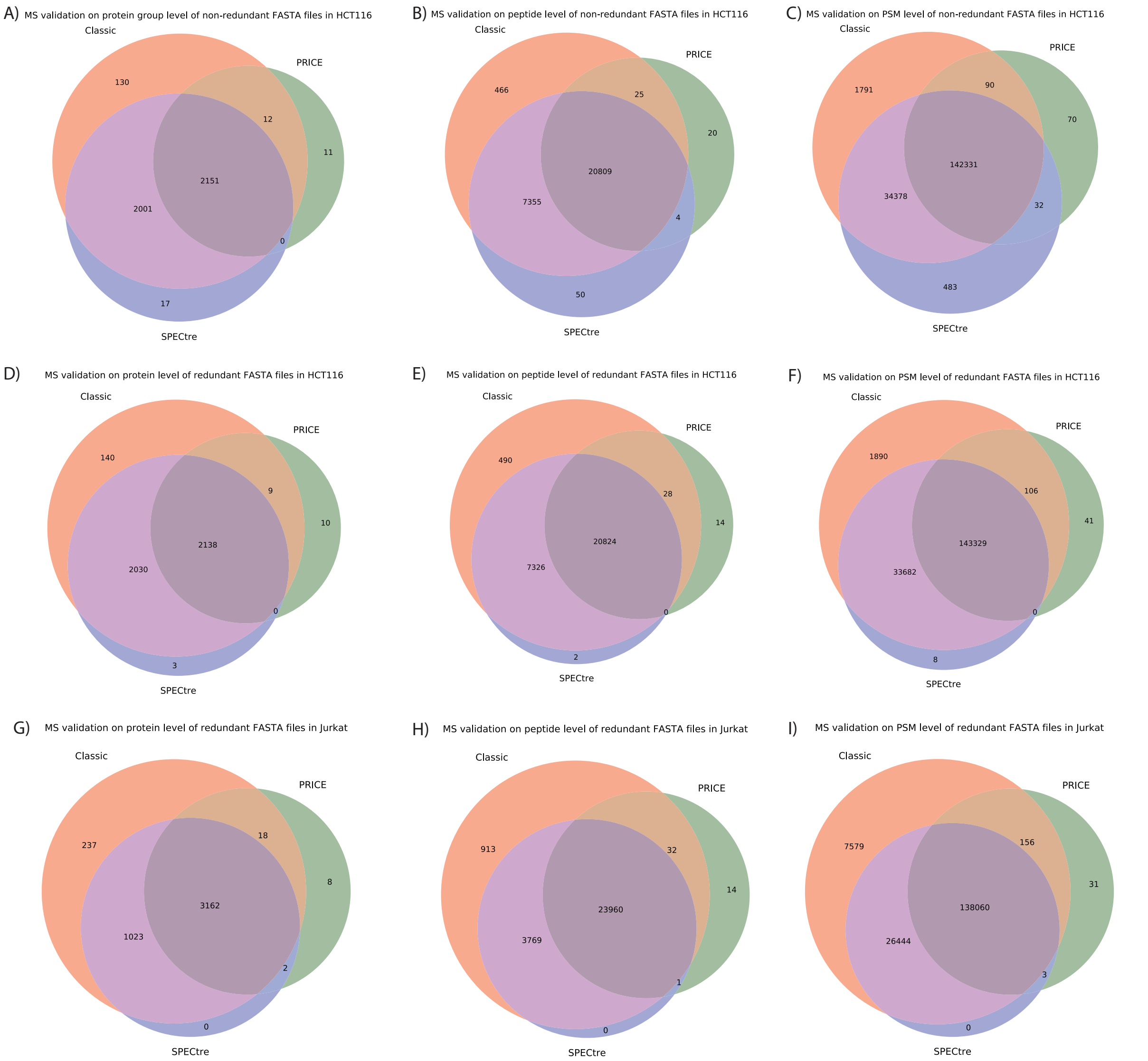

Supplementary Figure S4: Overview of the results of mass spectrometry searches against the combined FASTA files of different proteoform calling methods. Three different methods of proteoform calling (classic PROTEOFORMER with TIS calling, PRICE and SPECtre) were tried on ribosome profiling data and results were combined into one FASTA file. Mass spectrometry data were searched for these sequences and results were counted on the level of proteins, peptides and PSMs. (A-C) Results for HCT116 data against a redundancy-removed FASTA file. (D-F) Results for HCT116 data against a redundant FASTA file. (G-I)

Results for Jurkat data against a redundant FASTA file. The areas of the Venn diagrams are $log_{10}(x+1)$ *transformed for better visual representation.*

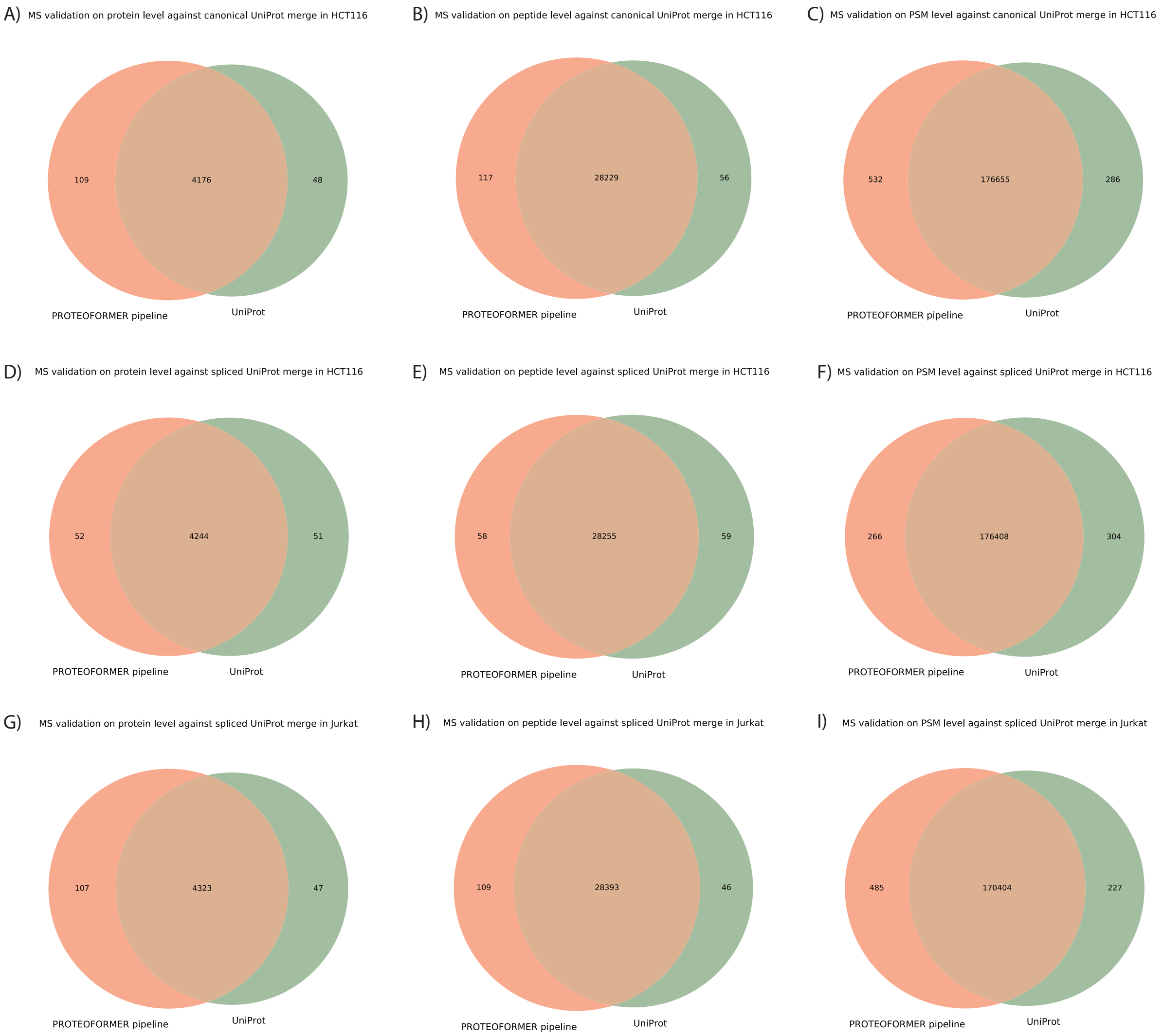

Supplementary Figure S5: Overview of the results of mass spectrometry searches against a merged database of the PROTEOFORMER pipeline and UniProt. Redundant FASTA files of the three proteoform calling methods of PROTEOFORMER were combined and afterwards merged with UniProt in one FASTA file. Mass spectrometry data were searched for these sequences with MaxQuant and the results were counted on the level of proteins, peptides and PSMs. (A-C) Results for HCT116 data against a merge with the canonical human UniProt. (D-F) Results for HCT116 data against a merge with the splice variant-included

human UniProt. (G-I) Results for Jurkat data against a merge with the splice variant-included human UniProt. The areas of the Venn diagrams are $log_{10}(x+1)$ *transformed for better visual representation.*

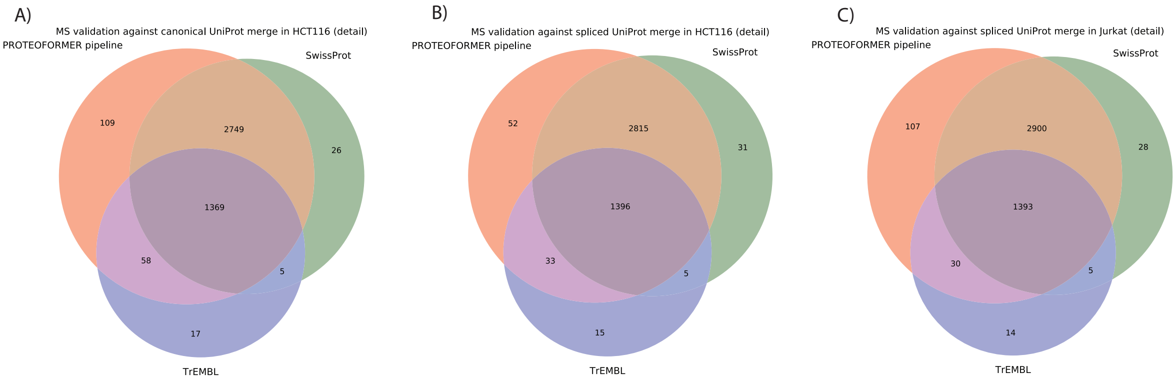

Supplementary Figure S6: Overview of the results of mass spectrometry searches against a merged database of the PROTEOFORMER pipeline and UniProt (split up between SwissProt and TrEMBL). This figure gives more details about the distribution of the validated UniProt sequences between SwissProt and TrEMBL. All counts are on protein level. Peptide and PSMs counts are not shown but are in the same trend. The areas of the Venn diagrams are $log_{10}(x+1)$ *transformed for better visual representation.*

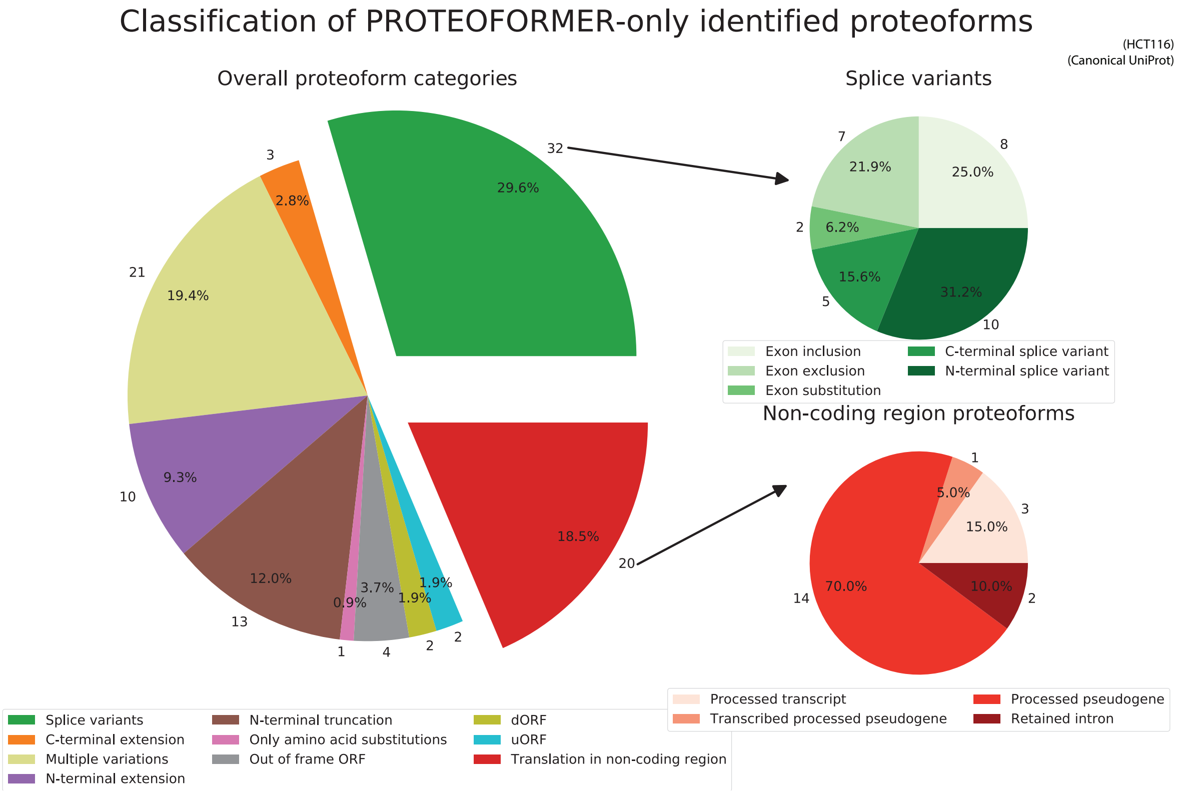

Supplementary Figure S7: Classification of the MS/MS-validated proteoforms found in PROTEOFORMER but not in the canonical UniProt database for HCT116 data. The proteoforms are classified based on the nature of their variation. For new splice variants and proteoforms originating from previously considered non-coding regions, more detailed classifications are added. More information about the different classifications is presented in Supplementary Table S3.

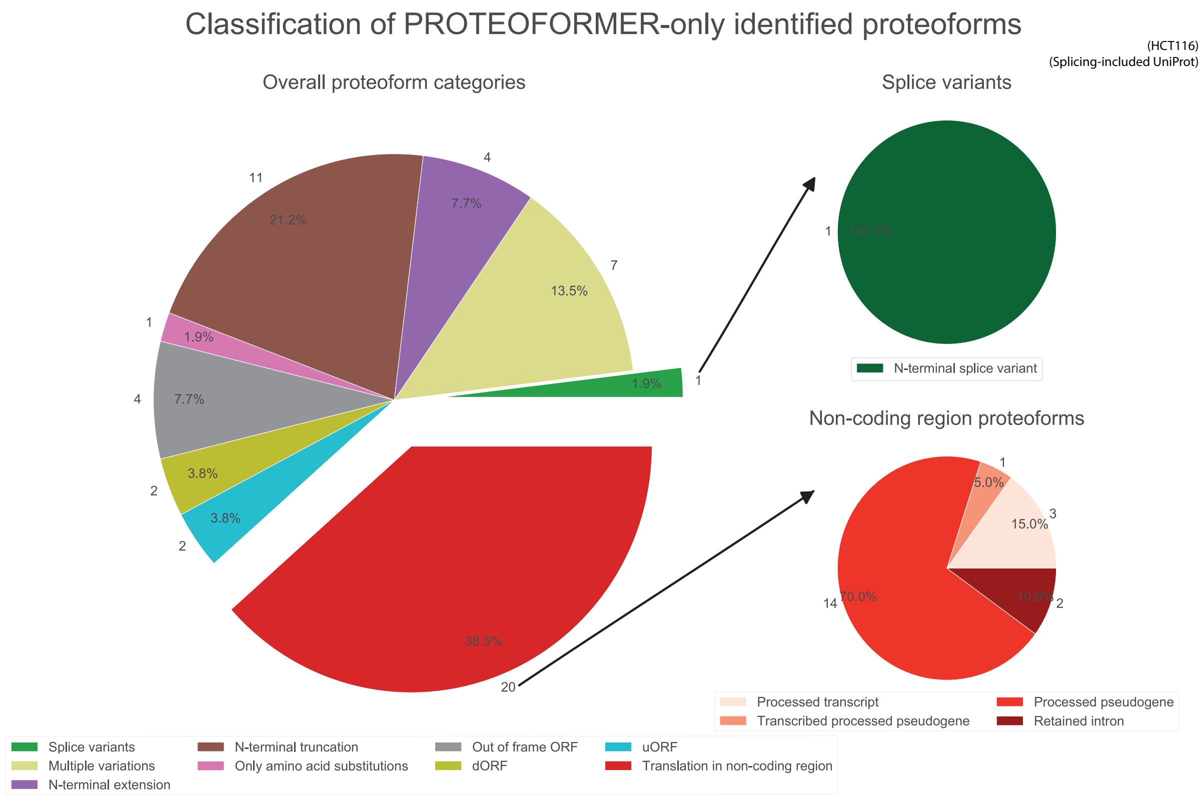

Supplementary Figure S8: Classification of the MS/MS-validated proteoforms found in PROTEOFORMER but not in the splicing-included UniProt database for HCT116 data. The proteoforms are classified based on the nature of their variation. For new splice variants and proteoforms originating from previously considered non-coding regions, more detailed classifications are added. More information about the different classifications is presented in Supplementary Table S3.

A) Combination of redundant fasta files in HEK293

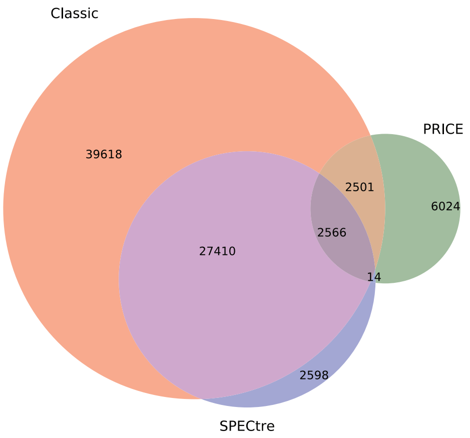

B) Combination of PROTEOFORMER results with spliced UniProt database for HEK293

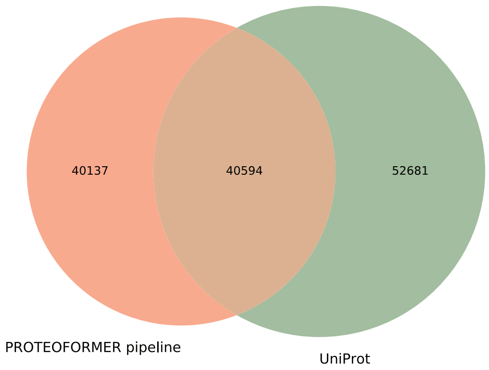

C) MS validation on protein level against spliced UniProt merge in HEK293

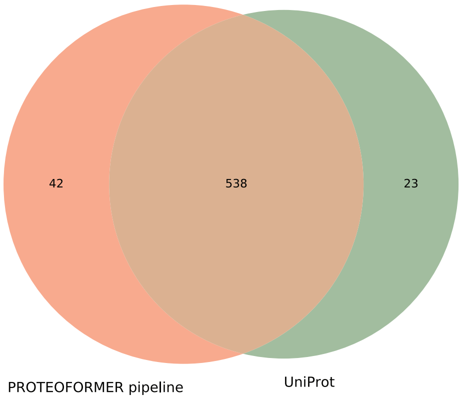

Supplementary Figure S9: Overview of the merging results for HEK293 data (data from online repositories). (A) Proteoform counts on ribosome profiling level for the three different proteoform calling techniques. (B) Combination of the proteoforms from subfigure A with the splicing-included UniProt database on ribosome profiling level. (C) Results of the mass spectrometry searches against the search space given in subfigure B.

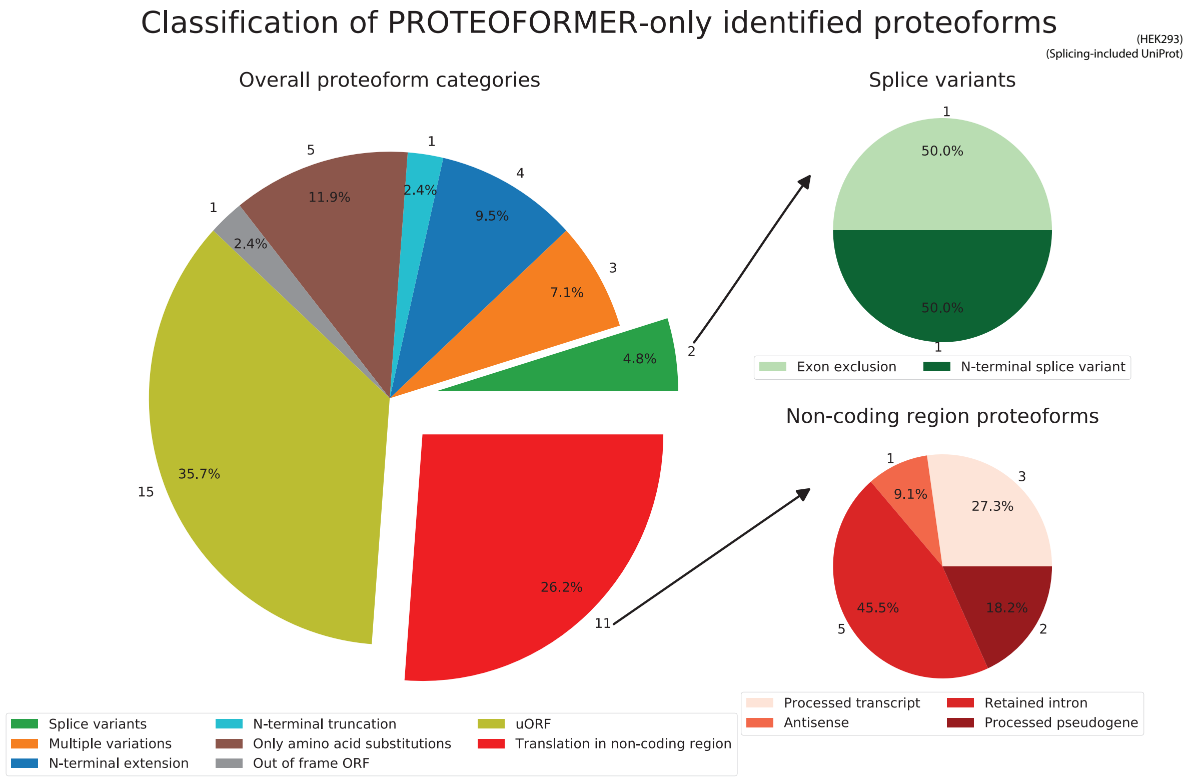

Supplementary Figure S10: Classification of the MS/MS-validated proteoforms found in PROTEOFORMER but not in the splicing-included UniProt database for HEK293 data (data from online repositories). The proteoforms are classified based on the nature of their variation. For new splice variants and proteoforms originating from previously considered non-coding regions, more detailed classifications are added. More information about the different classifications is presented in Supplementary Table S3. MS/MS data was obtained from an N-terminomics experiment (27), which leads to a considerable part of the proteoforms to be classified as uORF, N-terminal extension or N-terminal truncation.
