# Supplementary material for: PROTEOFORMER 2.0: Further Developments in the Ribosome Profiling-assisted Proteogenomic Hunt for New Proteoforms
Source: Mol Cell Proteomics. 2019 Apr 30;18(8 Suppl 1):S126–40. doi: 10.1074/mcp.RA118.001218 (PMC6692777; doi:10.1074/mcp.RA118.001218)
Supplement: Table S1 [file 142014_2_supp_322590_pqrw9j.pdf]

Sample	Treatment	Type	Total reads	Unique mapped	Multimapped	Total mapped	Unmapped	Unique mapped freq.	Multimapped freq.	Total mapped freq.
HCT116	CHX	PhiX	142 802 165			358 516	142 443 649			0,25%
		rRNA	142 443 649			105 649 115	36 794 534			74,17%
		snRNA	36 794 534			1 227 391	35 567 143			3,34%
		tRNA	35 567 143			4 434 117	31 133 026			12,47%
		Genomic	31 133 026	17 606 923	8 728 708	26 335 631	4 797 395	56,55%	28,04%	84,59%
	LTM	PhiX	151 696 882			395 021	151 301 861			0,26%
		rRNA	151 301 861			99 922 436	51 379 425			66,04%
		snRNA	51 379 425			2 119 809	49 259 616			4,13%
		tRNA	49 259 616			4 063 557	45 196 059			8,25%
		Genomic	45 196 059	25 692 092	11 403 645	37 095 737	8 100 322	56,85%	25,23%	82,08%
Jurkat	CHX	PhiX	361 801 191			2 826 450	358 974 741			0,78%
		rRNA	358 974 741			141 995 459	216 979 282			39,56%
		snRNA	216 979 282			25 914 185	191 065 097			11,94%
		tRNA	191 065 097			4 691 636	186 373 461			2,46%
		Genomic	186 373 461	124 995 073	0	124 995 073	61 378 388	67,07%	0,00%	67,07%
	LTM	PhiX	303 819 671			2 794 788	301 024 883			0,92%
		rRNA	301 024 883			119 058 484	181 966 399			39,55%
		snRNA	181 966 399			27 196 696	154 769 703			14,95%
		tRNA	154 769 703			13 357 840	141 411 863			8,63%
		Genomic	141 411 863	97 466 121	0	97 466 121	43 945 742	68,92%	0,00%	68,92%
