# Supplementary material for: PROTEOFORMER 2.0: Further Developments in the Ribosome Profiling-assisted Proteogenomic Hunt for New Proteoforms
Source: Mol Cell Proteomics. 2019 Apr 30;18(8 Suppl 1):S126–40. doi: 10.1074/mcp.RA118.001218 (PMC6692777; doi:10.1074/mcp.RA118.001218)
Supplement: Table S2 [file 142014_2_supp_322591_pqrw9j.pdf]

Sample	Redundancy	UniProt	Sequences in search space	Identified protein groups	Identified peptides	Identified PSMs
HCT116	No	/	92 931	4 322	28 729	179 175
	Yes	/	133 051	4 330	28 684	179 056
Jurkat	Yes	/	201 419	4 450	28 689	172 273
HCT116	Yes	Canonical	176 202	4 333	28 402	177 473
		Spliced	186 627	4 347	28 372	176 978
Jurkat	Yes	Spliced	253 734	4 477	28 548	171 116
HCT116, only UniProt		Canonical	71 356	4 294	28 443	180 526
