# Supplementary material for: PROTEOFORMER 2.0: Further Developments in the Ribosome Profiling-assisted Proteogenomic Hunt for New Proteoforms
Source: Mol Cell Proteomics. 2019 Apr 30;18(8 Suppl 1):S126–40. doi: 10.1074/mcp.RA118.001218 (PMC6692777; doi:10.1074/mcp.RA118.001218)
Supplement: Table S3 [file 142014_2_supp_322592_pqrw9j.pdf]

Proteoform class	Description
Splice variants	Proteoforms with exon variations compared to the canonical form.
Exon inclusion	The proteoform contains additional internal exons.
Exon exclusion	The proteoform lacks one or more internal exons.
Exon substitution	An exon of the canonical protein has been replaced for another exon.
C-terminal splice variant	A C-terminal exon or exon part has been replaced.
N-terminal splice variant	An N-terminal exon or exon part has been replaced.
Translation in non-coding region	Translated proteoforms in formerly considered untranslated transcripts. Subcategories based on Ensembl biotypes.
Processed transcript	Transcripts that do not contain a known ORF, like lncRNAs and ncRNAs.
Processed pseudogene	Pseudogenes are similar to known proteins but they contain a frameshift and/or stop codon that disrupts the ORF. Processed pseudogenes lack introns and are thought to arise from reverse mRNA transcription and DNA reinsertion.
Transcribed processed pseudogene	Protein homology or genomic structure indicates that it is a pseudogene but the presence of locus-specific transcripts indicates expression.
Retained intron	Proteoform translated from a transcript that has intronic sequences compared to other coding transcripts.
C-terminal extension	Proteoform with extra sequence information added to the C-terminus.
Multiple variations	Proteoform with a combination of variants from the other categories, making it difficult to place it in one categorie. Manual inspection can lead to classification.
N-terminal extension	Proteoform with extra sequence information added to the N-terminus. Translation starts in the earlier considered 5' untranslated region and continues over the canonical initiation site.
N-terminal truncation	Proteoform lacking a sequence part at the N-terminus.
Only amino acid substitutions	Proteoform differing from the canonical form only by single amino acid variations.
Out of frame ORF	Translation product contained in frame +1 or +2 of a known ORF.
dORF	Translation product originating from the 3' untranslated region of a known protein-coding transcript.
uORF	Translation product originating from the 5' untranslated region of a known protein-coding transcript.
