# Supplementary material for: PROTEOFORMER 2.0: Further Developments in the Ribosome Profiling-assisted Proteogenomic Hunt for New Proteoforms
Source: Mol Cell Proteomics. 2019 Apr 30;18(8 Suppl 1):S126–40. doi: 10.1074/mcp.RA118.001218 (PMC6692777; doi:10.1074/mcp.RA118.001218)
Supplement: Supplemental file S1 [file 142014_2_supp_322593_pqrw9j.pdf]

FastQC Report

Thu 26 Apr 2018
HCT116_CHX.fastq

Summary

- Basic Statistics
- Per base sequence quality
- Per tile sequence quality
- Per sequence quality scores
- Per base sequence content
- Per sequence GC content
- Per base N content
- Sequence Length Distribution
- Sequence Duplication Levels
- Overrepresented sequences
- Adapter Content
- Kmer Content

Basic Statistics

Measure	Value
Filename	HCT116_CHX.fastq
File type	Conventional base calls
Encoding	Sanger / Illumina 1.9
Total Sequences	155006923
Sequences flagged as poor quality	0
Sequence length	50
%GC	57

Per base sequence quality

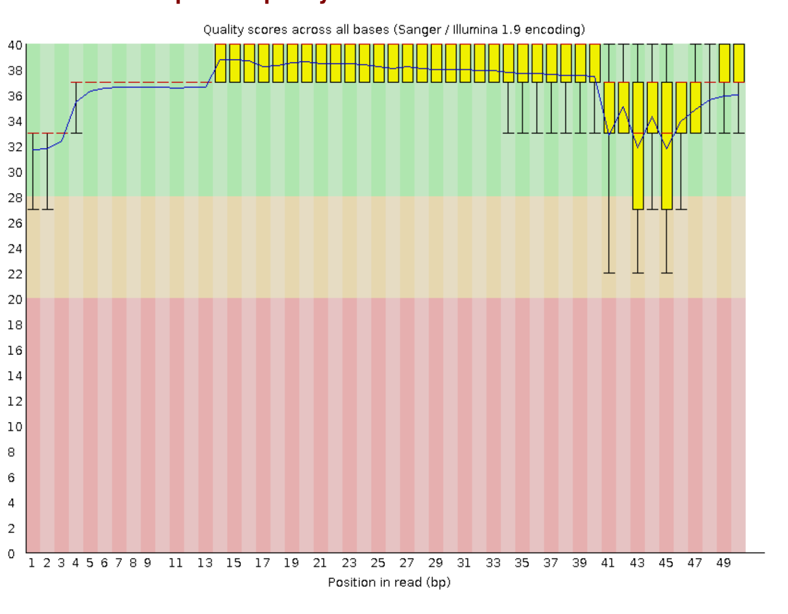

Per tile sequence quality

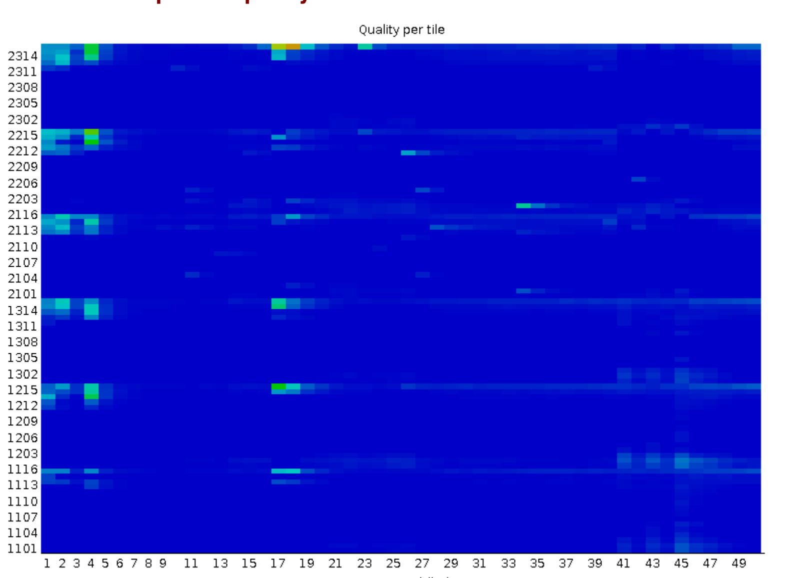

Per sequence quality scores

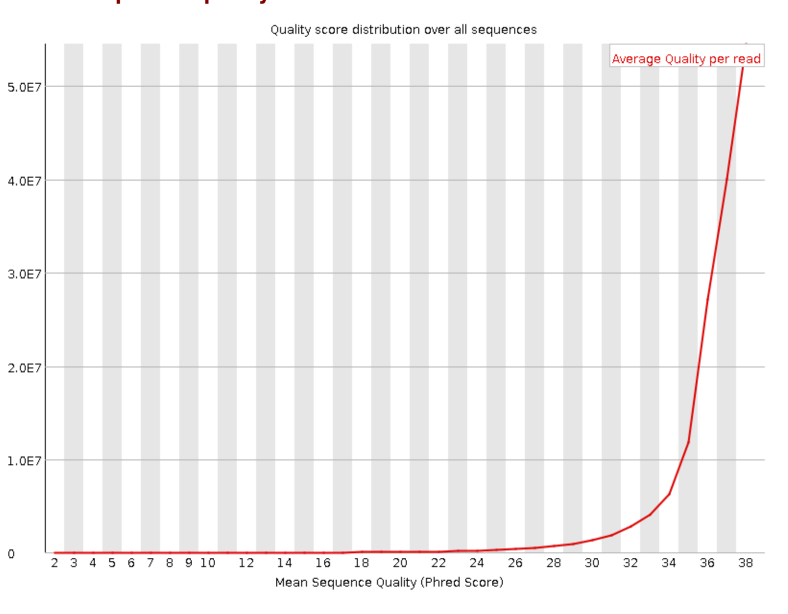

Per base sequence content

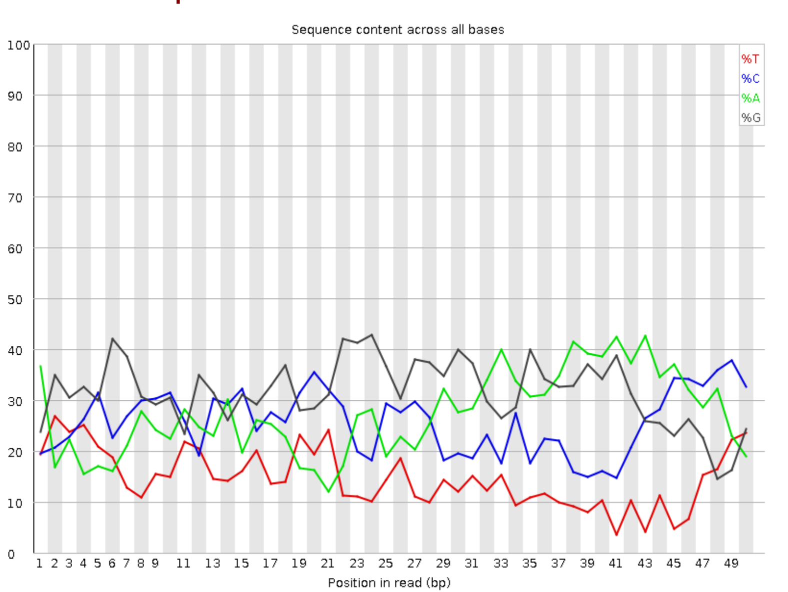

Per sequence GC content

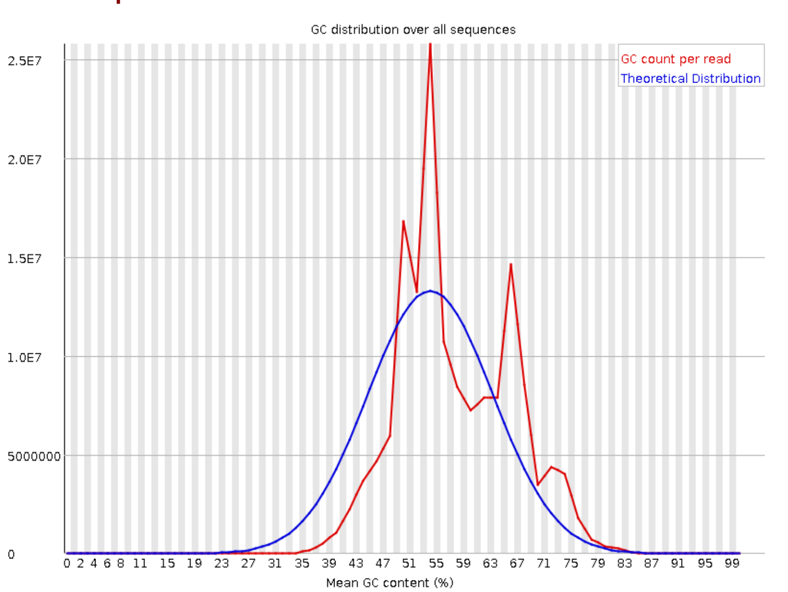

Per base N content

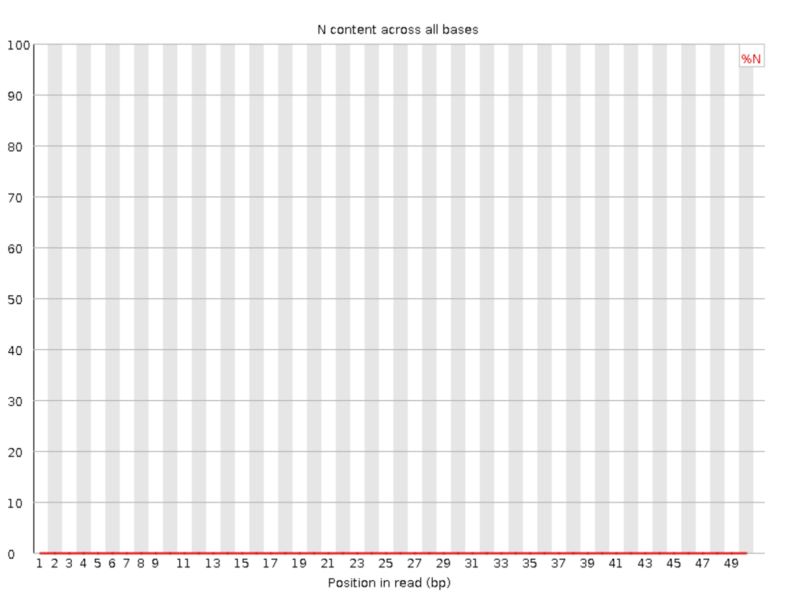

Sequence Length Distribution

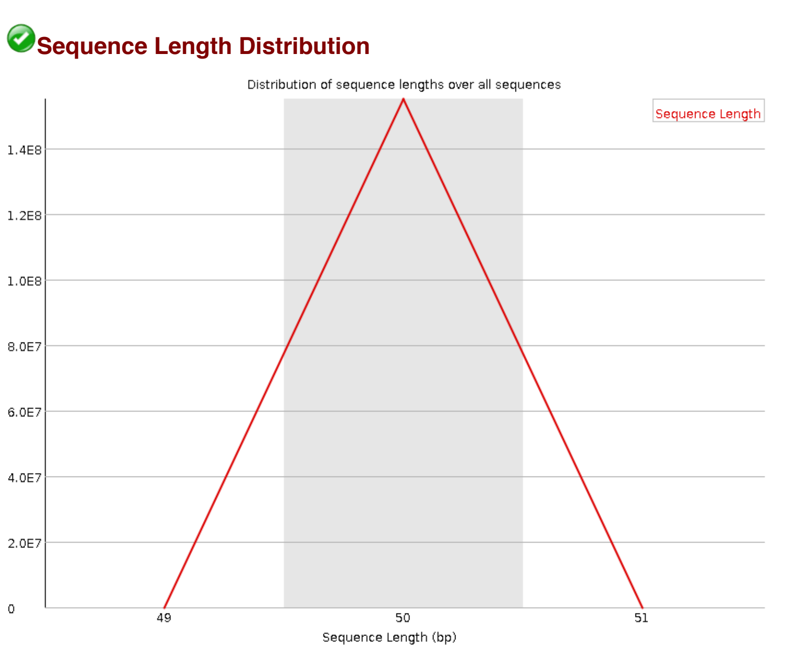

Sequence Duplication Levels

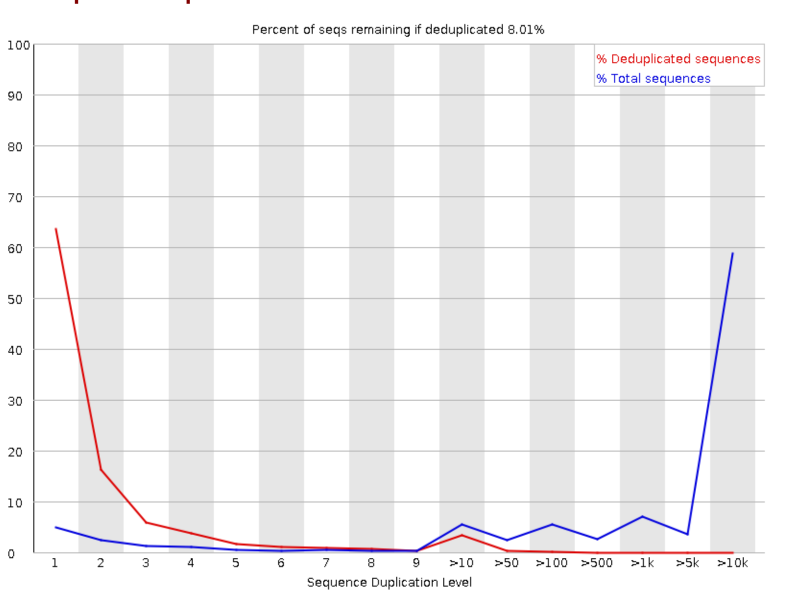

Overrepresented sequences

Sequence	Count	Percentage	Possible Source
AGATCGGAAGAGCACACGTCTGAACTCCAGTCACGCCAATATCTCGTATG	8723099	5.6275544544549145	TruSeq Adapter, Index 6 (100% over 49bp)
TTTGGTGACTCTAGATAACCTCGGGCCGATCGCACAGATCGGAAGAGCAC	5219438	3.3672289591865514	No Hit
TTGGTGACTCTAGATAACCTCGGGCCGATCGCACAGATCGGAAGAGCACA	4838440	3.121434776174481	No Hit
CACTCGCCGAATCCCGGGGCCGAGGGAGCGAAGATCGGAAGAGCACACGT	3020622	1.948701349293928	No Hit
GTTGAACCCCATTCGTGATGGGGATCGGGGATTAGATCGGAAGAGCACAC	2937074	1.8948018212063984	No Hit
ACTCGCCGAATCCCGGGGCCGAGGGAGCGAAGATCGGAAGAGCACACGTC	2226972	1.4366919598810435	No Hit
GCACTCGCCGAATCCCGGGGCCGAGGGAGCGAAGATCGGAAGAGCACACG	1976994	1.2754230338473334	No Hit
CGATCAAAAGGGACAAGCATCAAGCACGCAGCAGATCGGAAGAGCACACG	1782809	1.150147984035526	No Hit
CCGCCGAGGGCGCACCACCGGCCCGTCTCGCAGATCGGAAGAGCACACGT	1038491	0.6699642699184475	No Hit
GGTGCGCCGCGACCGGCTCCGGGACGGCTGGGAGATCGGAAGAGCACACG	934719	0.6030175826404863	No Hit
AGCACTCGCCGAATCCCGGGGCCGAGGGAGCGAGATCGGAAGAGCACACG	910906	0.5876550429944345	No Hit
AAGCTTGGGCTGCAGGTCGACCCGTGCAGATCGGAAGAGCACACGTCTGA	891048	0.5748440022901429	Illumina Multiplexing PCR Primer 2.01 (100% over 23bp)
CTCCCGGGGCTACGCCTGTCTGAGCGTCGCTTAGATCGGAAGAGCACACG	883714	0.5701126007126791	No Hit
AGATAACCTCGGGCCGATCGCACAGATCGGAAGAGCACACGTCTGAACTC	880452	0.5680081785766433	Illumina Multiplexing PCR Primer 2.01 (100% over 27bp)
ACTCGCCGAATCCCGGGGCCGAGGGAGCGAGATCGGAAGAGCACACGTCT	810069	0.522601819532925	Illumina Multiplexing PCR Primer 2.01 (100% over 21bp)
AGCCCGTGGACGGTGTGAGGCCGGTAGATCGGAAGAGCACACGTCTGAAC	785886	0.5070005808708299	Illumina Multiplexing PCR Primer 2.01 (100% over 25bp)
TTTTGGTGACTCTAGATAACCTCGGGCCGATCGCACAGATCGGAAGAGCA	727536	0.4693571009083252	No Hit
AGCACTCGCCGAATCCCGGGGCCGAGGGAGCGAAGATCGGAAGAGCACAC	700082	0.4516456339179122	No Hit
CGTGGGGGGCCCAAGTCCTTCTGATCGAGGCCAGATCGGAAGAGCACACG	631091	0.4071372992804973	No Hit
CGGTGCGCCGCGACCGGCTCCGGGACGGCTGGGAGATCGGAAGAGCACAC	590633	0.3810365295748758	No Hit
TTGGTGACTCTAGATAACCTCGGGCCGATCGCAGATCGGAAGAGCACACG	587725	0.3791604843352706	No Hit
GCCGCCTGGATACCGCAGCTAGGAATAATGGAATAGATCGGAAGAGCACA	576588	0.3719756439523672	No Hit
GTGGGGGGCCCAAGTCCTTCTGATCGAGGCCAGATCGGAAGAGCACACGT	568189	0.36655717628818424	No Hit
ACGATCAAAAGGGACAAGCATCAAGCACGCAGCAGATCGGAAGAGCACAC	551090	0.3555260560846047	No Hit
AGCACTCGCCGAATCCCGGGGCCGAGGGAGCAGATCGGAAGAGCACACGT	544436	0.35123334459068	No Hit
GTGGGGGGCCCAAGTCCTTCTGATCGAGGCAGATCGGAAGAGCACACGTC	474517	0.3061263270157295	No Hit
TTTGGTGACTCTAGATAACCTCGGGCCGATCGCAGATCGGAAGAGCACAC	472376	0.30474509838505726	No Hit
ACTCTGGTGGAGGTCCGTAGCGGTCCTGACGTGCAGATCGGAAGAGCACA	464101	0.29940662714787264	No Hit
TTTGGTGACTCTAGATAACCTCGGGCCGATCAGATCGGAAGAGCACACGT	451188	0.29107603148796135	No Hit
AGCCCGTGGACGGTGTGAGGCCGGTAGCGGCCCCCGAGATCGGAAGAGCA	431635	0.2784617561887865	No Hit
CGGGAGGGAGGAAGACGAACGGAAGGACAGATCGGAAGAGCACACGTCTG	391892	0.2528222562033568	Illumina Multiplexing PCR Primer 2.01 (100% over 22bp)
AGCCCGTGGACGGTGTGAGGCCGGTAGCGAGATCGGAAGAGCACACGTCT	391158	0.2523487289661249	Illumina Multiplexing PCR Primer 2.01 (100% over 21bp)
ACTCGCCGAATCCCGGGGCCGAGGGAGCAGATCGGAAGAGCACACGTCTG	388235	0.2504630067393829	Illumina Multiplexing PCR Primer 2.01 (100% over 22bp)
TTGGTGACTCTAGATAACCTCGGGCCGATCAGATCGGAAGAGCACACGTC	387053	0.24970046015299588	No Hit
AGCACTCGCCGAATCCCGGGGCCGAGGGAGCGAGAGATCGGAAGAGCACA	374849	0.24182726341842165	No Hit
TTTGGTGACTCTAGATAACCTCGGGCCGATCGCAAGATCGGAAGAGCACA	368522	0.23774551024408117	No Hit
GAATACAAGCTTGGGCTGCAGGTCGACCCGAGATCGGAAGAGCACACGTC	350919	0.2263892432727021	No Hit
TTGGTGACTCTAGATAACCTCGGGCCGATCGCAAGATCGGAAGAGCACAC	349551	0.2255067020458177	No Hit
ACCGGGGCCCGCGCGGCCAACCCCCGGGACGGGGACAGATCGGAAGAGCA	333393	0.21508265150195904	No Hit
ACTCCCGACCCGGGGAGGTAGTGACGAAAAATAACAGATCGGAAGAGCAC	331429	0.21381561131950216	No Hit
CGTGGGGGGCCCAAGTCCTTCTGATCGAGGCAGATCGGAAGAGCACACGT	328940	0.2122098765872541	No Hit
GCACTCGCCGAATCCCGGGGCCGAGGGAGCGAGATCGGAAGAGCACACGT	306214	0.19754859594238897	No Hit
CGCGGGAGACCGGGGTTCGATTCCCCGACGGGGAAGATCGGAAGAGCACA	290034	0.1871103524840629	No Hit
GCACTCGCCGAATCCCGGGGCCGAGGGAGCGAGAGATCGGAAGAGCACAC	276186	0.17817655795928547	No Hit
AGGGCGAAGCCAGAGGAAACTCTGGTGGAGGTCCAGATCGGAAGAGCACA	275772	0.1779094731143073	No Hit
TCCCGGGGCTACGCCTGTCTGAGCGTCGCTTAGATCGGAAGAGCACACGT	271890	0.17540506884328	No Hit
ATGTGGCGTACGGAAGACCCGCTCCCCGGCGCAGATCGGAAGAGCACACG	267332	0.17246455501861682	No Hit
TGGATAACTGTGGTAATTCTAGAGCTAATACAAGATCGGAAGAGCACACG	262846	0.1695704907322107	No Hit
ATGTGGCGTACGGAAGACCCGCTCCCCGGCGAGATCGGAAGAGCACACGT	255954	0.16512423770904736	No Hit
TAGATAACCTCGGGCCGATCGCACAGATCGGAAGAGCACACGTCTGAACT	248271	0.16016768489753197	Illumina Multiplexing PCR Primer 2.01 (100% over 26bp)
ACTCGCCGAATCCCGGGGCCGAGGGAGAGATCGGAAGAGCACACGTCTGA	245921	0.1586516235794191	Illumina Multiplexing PCR Primer 2.01 (100% over 23bp)
CTTTGGTGACTCTAGATAACCTCGGGCCGATCGCACAGATCGGAAGAGCA	239179	0.15430214042762463	No Hit
AGCCCGTGGACGGTGTGAGGCCGGTAGCGGCCCCCAGATCGGAAGAGCAC	236193	0.1523757748549076	No Hit
CTCCCGGGGCTACGCCTGTCTGAGCGTCGCTAGATCGGAAGAGCACACGT	233665	0.15074487995610364	No Hit
CTCTGGTGGAGGTCCGTAGCGGTCCTGACGTGCAGATCGGAAGAGCACAC	232163	0.149775890977463	No Hit
TGGTGACTCTAGATAACCTCGGGCCGATCGCACAGATCGGAAGAGCACAC	228060	0.1471289124292855	No Hit
CCGCCTGGATACCGCAGCTAGGAATAATGGAATAGATCGGAAGAGCACAC	222398	0.14347617235134716	No Hit
TTTGGTGACTCTAGATAACCTCGGGCAGATCGGAAGAGCACACGTCTGAA	210443	0.13576361360324532	Illumina Multiplexing PCR Primer 2.01 (100% over 24bp)
GCGCCCGATCTCGTCTGATCTCGGAAGCTAAGCAGATCGGAAGAGCACAC	206405	0.13315856866599435	No Hit
GTGGACGGTGTGAGGCCGGTAGCGGCCCCCGAGATCGGAAGAGCACACGT	206322	0.13310502267050356	No Hit
GCGACCTCAGATCAGACGTGGCGACCCGCTGAAAGATCGGAAGAGCACAC	203917	0.13155347906622208	No Hit
AAGCTTGGGCTGCAGGTCGACCCGTGCGGAGGAGCAGATCGGAAGAGCAC	200712	0.12948582948130646	No Hit
GGTGACTCTAGATAACCTCGGGCCGATCGCACAGATCGGAAGAGCACACG	194225	0.12530085511083916	No Hit
AGCATCGCGAAGGCCCGCGGCGGGTGTTGACGCGATGAGATCGGAAGAGC	187013	0.12064815969542213	No Hit
AGGATTCAACCCGGCGGCGGGTCCGGCCGTGAGATCGGAAGAGCACACGT	185422	0.11962175392643591	No Hit
GGGAGACCGGGGTTCGATTCCCCGACGGGGAAGATCGGAAGAGCACACGT	184777	0.11920564347954962	No Hit
GCGACCTCAGATCAGACGTGGCGACCCGCTGAAGATCGGAAGAGCACACG	182023	0.11742894864121649	No Hit
ATGGAAGTCGGAATCCGCTAAGGAGTGTGTAACAGATCGGAAGAGCACAC	181265	0.11693993822456561	No Hit
AGTGAAAAAACGATCAGAGTAGTGGTATTTCAGATCGGAAGAGCACACGT	179133	0.11556451578617556	No Hit
CGGGAGACCGGGGTTCGATTCCCCGACGGGGAAGATCGGAAGAGCACACG	178463	0.11513227702739444	No Hit
AATCGGTAGTAGCGACGGGCGGTGTGTACAGATCGGAAGAGCACACGTCT	176803	0.11406135711757855	Illumina Multiplexing PCR Primer 2.01 (100% over 21bp)
GAACGGAAGGACGGACGGCGCCGGACGCGCAGATCGGAAGAGCACACGTC	174960	0.11287237796469259	No Hit
TTGGTGACTCTAGATAACCTCGGGCAGATCGGAAGAGCACACGTCTGAAC	170037	0.10969639078636506	Illumina Multiplexing PCR Primer 2.01 (100% over 25bp)
AACCTCGGGCCGATCGCACAGATCGGAAGAGCACACGTCTGAACTCCAGT	169263	0.10919705825010151	Illumina Multiplexing PCR Primer 2.01 (100% over 31bp)
CGTTGAACCCCATTCGTGATGGGGATCGGGGATTAGATCGGAAGAGCACA	169126	0.10910867510091792	No Hit
AGACCCCAGAAAAGGTGTTGGTTGATATAGACAGATCGGAAGAGCACACG	167994	0.10837838513832057	No Hit
GAGCCGCCCCGGCGGTGAACGGGGAGGAGGCGGGAACAGATCGGAAGAGC	165515	0.10677910173083044	No Hit
CACTCGCCGAATCCCGGGGCCGAGGGAGCGAGATCGGAAGAGCACACGTC	164268	0.10597462153351693	No Hit
CTAGATAACCTCGGGCCGATCGCACAGATCGGAAGAGCACACGTCTGAAC	163432	0.10543529078375423	Illumina Multiplexing PCR Primer 2.01 (100% over 25bp)
GTTGAACCCCATTCGTGATGGGGATCGGGGATTGAGATCGGAAGAGCACA	160422	0.10349344203161816	No Hit
AAGCTTGGGCTGCAGGTCGACCCGAGATCGGAAGAGCACACGTCTGAACT	160056	0.10325732354547802	Illumina Multiplexing PCR Primer 2.01 (100% over 26bp)
TCTAGATAACCTCGGGCCGATCGCACAGATCGGAAGAGCACACGTCTGAA	159115	0.10265025388575709	Illumina Multiplexing PCR Primer 2.01 (100% over 24bp)
CGGCGGTGCGCCGCGACCGGCTCCGGGACGGCTGGGAGATCGGAAGAGCA	155862	0.10055163794200339	No Hit
GGCGGTGCGCCGCGACCGGCTCCGGGACGGCTGGGAGATCGGAAGAGCAC	155556	0.10035422740441084	No Hit

Adapter Content

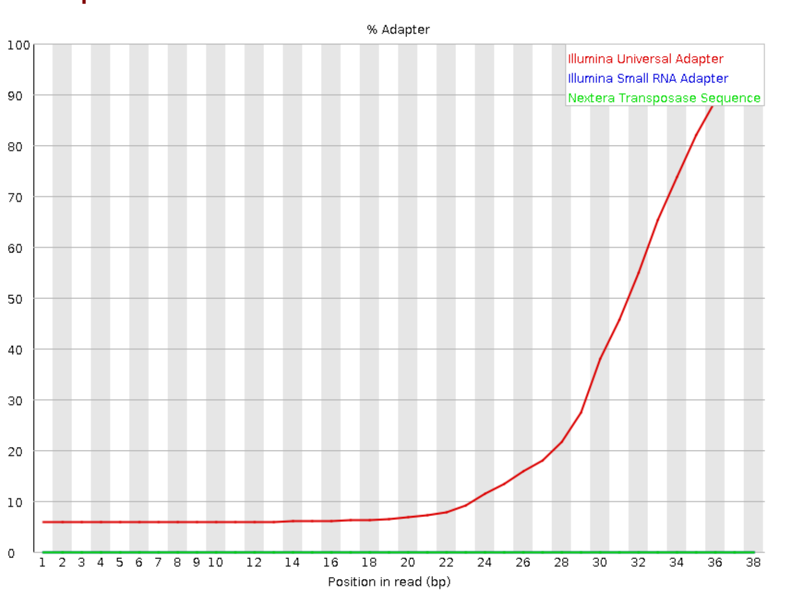

Kmer Content

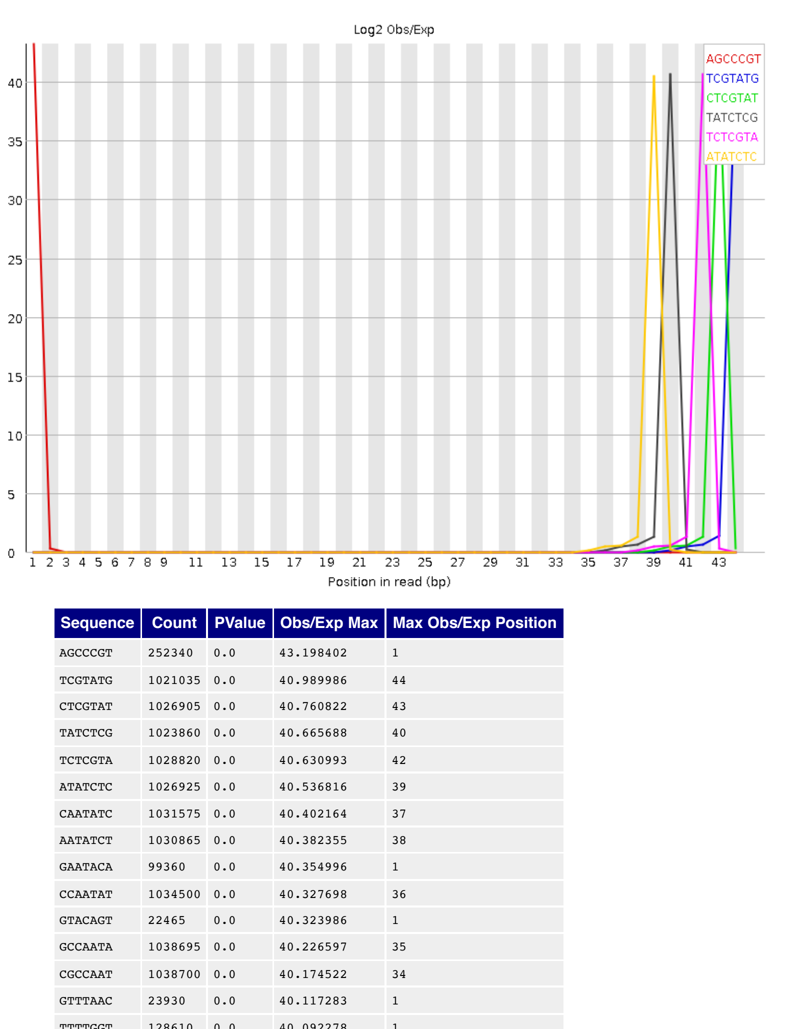

Sequence	Count	PValue	Obs/Exp Max	Max Obs/Exp Position
AGCCCGT	252340	0.0	43.198402	1
TCGTATG	1021035	0.0	40.989986	44
CTCGTAT	1026905	0.0	40.760822	43
TATCTCG	1023860	0.0	40.665688	40
TCTCGTA	1028820	0.0	40.630993	42
ATATCTC	1026925	0.0	40.536816	39
CAATATC	1031575	0.0	40.402164	37
AATATCT	1030865	0.0	40.382355	38
GAATACA	99360	0.0	40.354996	1
CCAATAT	1034500	0.0	40.327698	36
GTACAGT	22465	0.0	40.323986	1
GCCAATA	1038695	0.0	40.226597	35
CGCCAAT	1038700	0.0	40.174522	34
GTTTAAC	23930	0.0	40.117283	1
TTTTGGT	128610	0.0	40.092278	1
ACGCCAA	1052485	0.0	39.84175	33
TAACCCC	25320	0.0	39.817616	4
CACGCCA	1057570	0.0	39.675858	32
TCACGCC	1061400	0.0	39.537346	31
TTGAACC	405935	0.0	39.518867	2

Produced by FastQC (version 0.11.2)
