# Supplementary material for: PROTEOFORMER 2.0: Further Developments in the Ribosome Profiling-assisted Proteogenomic Hunt for New Proteoforms
Source: Mol Cell Proteomics. 2019 Apr 30;18(8 Suppl 1):S126–40. doi: 10.1074/mcp.RA118.001218 (PMC6692777; doi:10.1074/mcp.RA118.001218)
Supplement: Supplemental file S2 [file 142014_2_supp_322594_pqrw9j.pdf]

FastQC Report

Thu 26 Apr 2018
HCT116_LTM.fastq

Summary

- Basic Statistics
- Per base sequence quality
- Per tile sequence quality
- Per sequence quality scores
- Per base sequence content
- Per sequence GC content
- Per base N content
- Sequence Length Distribution
- Sequence Duplication Levels
- Overrepresented sequences
- Adapter Content
- Kmer Content

Basic Statistics

Measure	Value
Filename	HCT116_LTM.fastq
File type	Conventional base calls
Encoding	Sanger / Illumina 1.9
Total Sequences	158957695
Sequences flagged as poor quality	0
Sequence length	50
%GC	57

Per base sequence quality

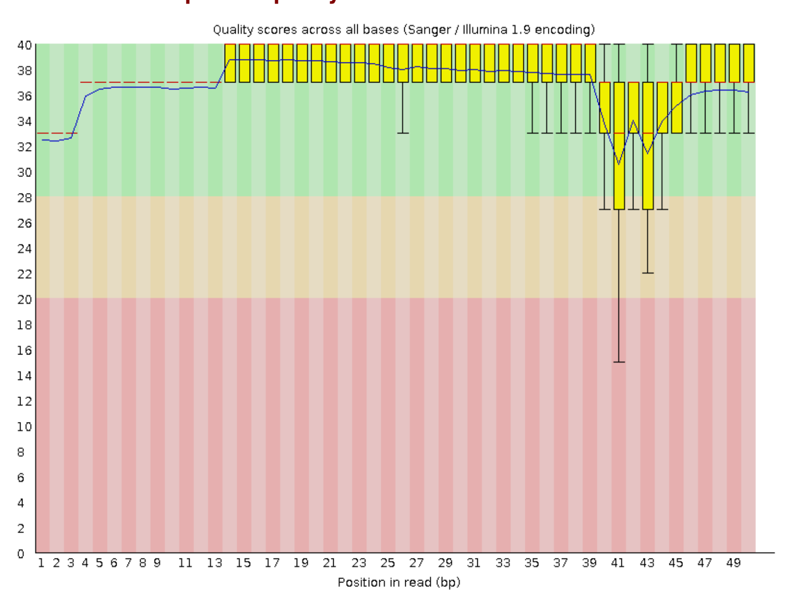

Per tile sequence quality

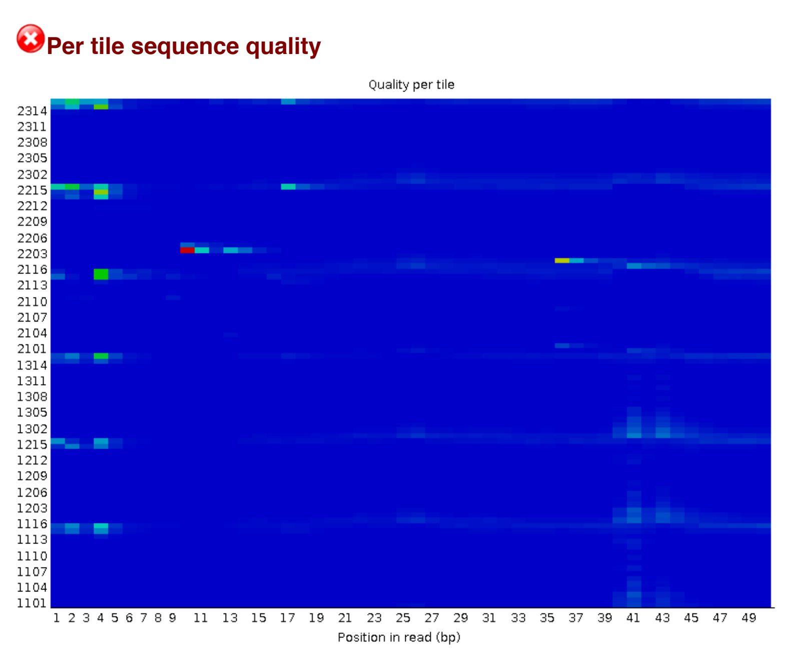

Per sequence quality scores

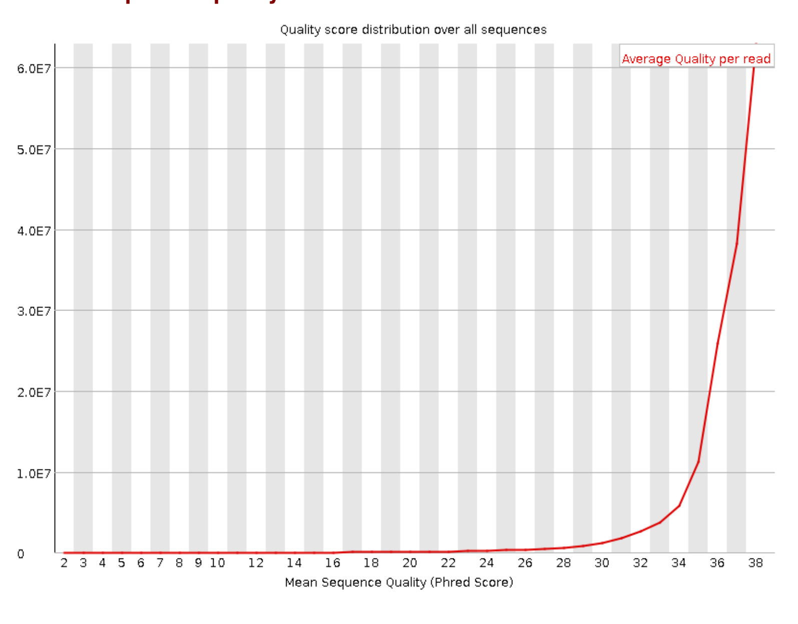

Per base sequence content

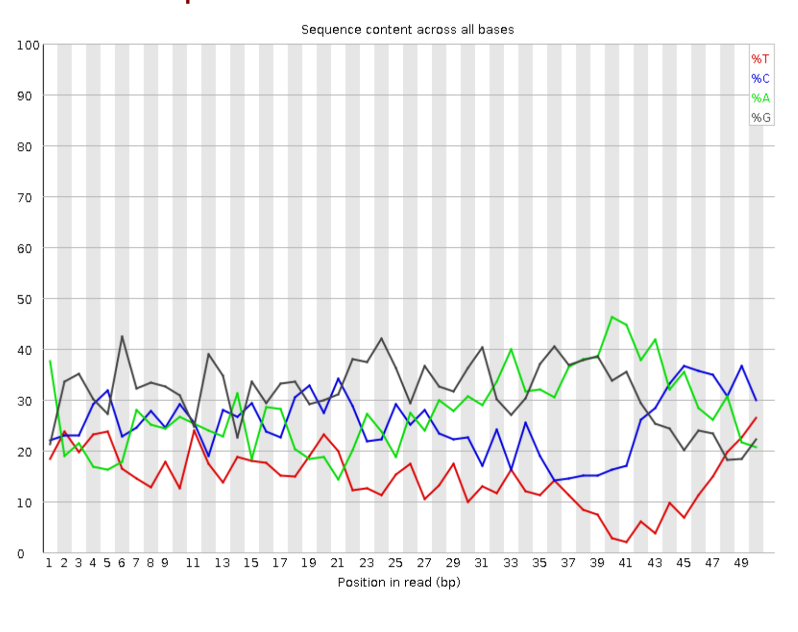

Per sequence GC content

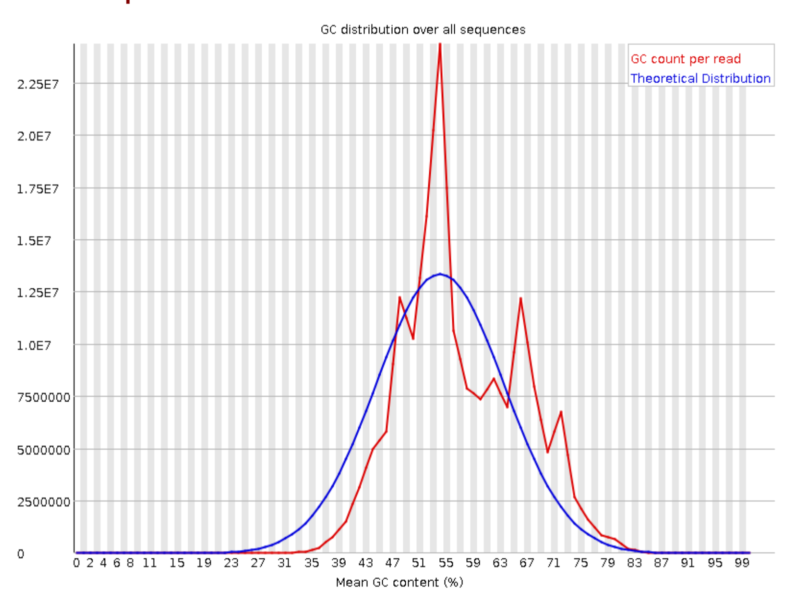

Per base N content

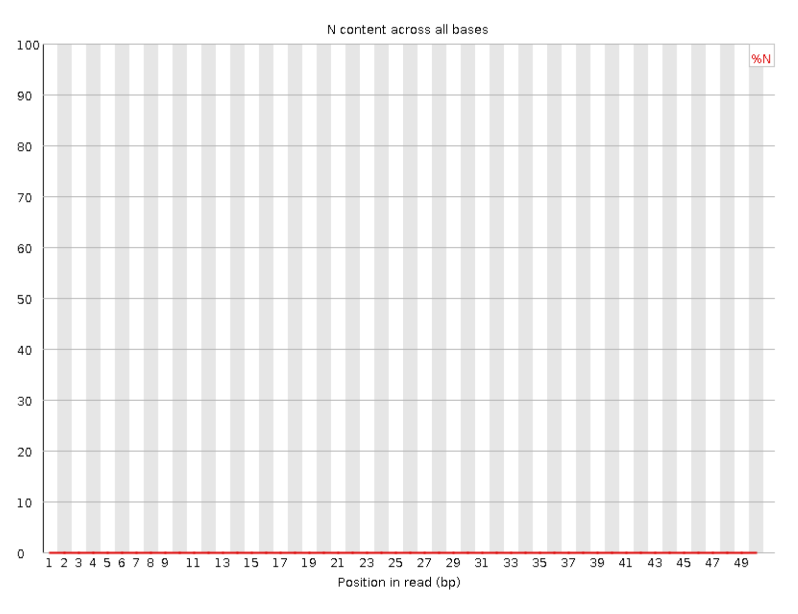

Sequence Length Distribution

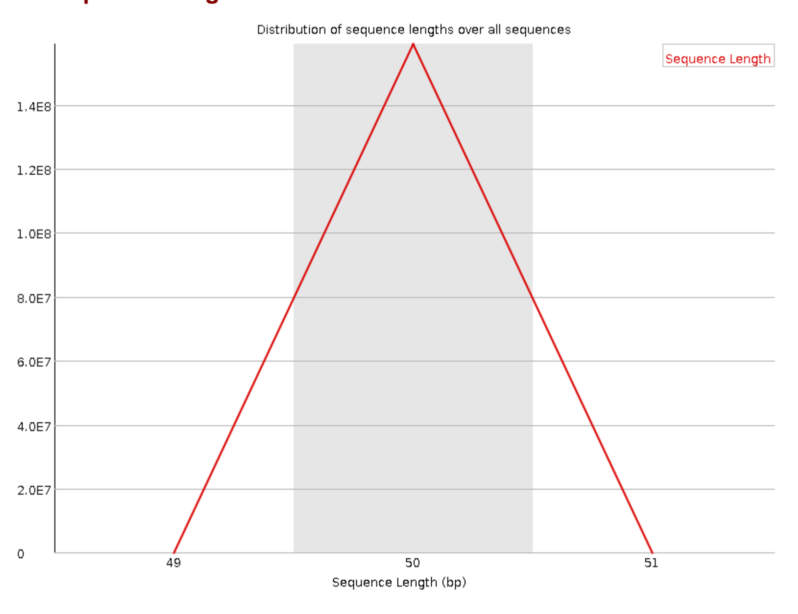

Sequence Duplication Levels

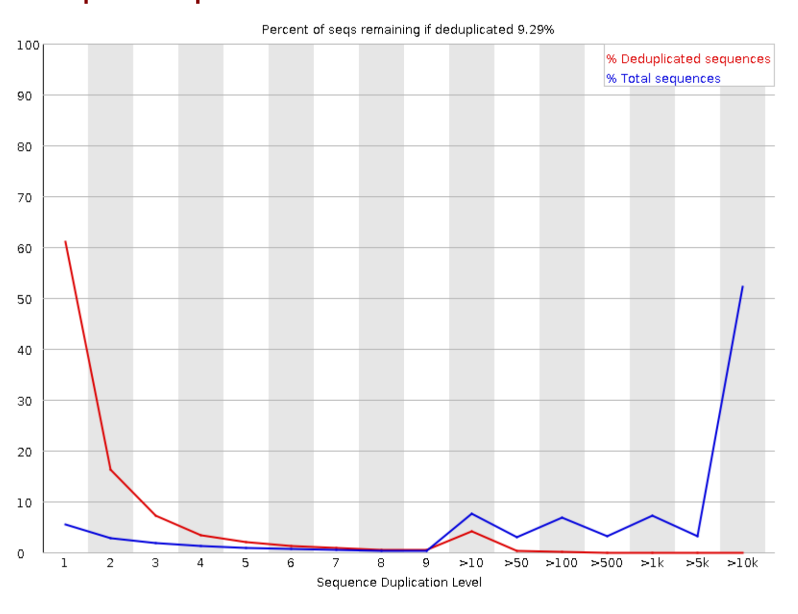

Overrepresented sequences

Sequence	Count	Percentage	Possible Source
TTGGTGACTCTAGATAACCTCGGGCCGATCGCACAGATCGGAAGAGCACA	5644087	3.5506849794217263	No Hit
AGATCGGAAGAGCACACGTCTGAACTCCAGTCACCTTGTAATCTCGTATG	4694321	2.9531888972094116	TruSeq Adapter, Index 12 (100% over 49bp)
CGATCAAAAGGGACAAGCATCAAGCACGCAGCAGATCGGAAGAGCACACG	4006562	2.520520947413084	No Hit
CACTCGCCGAATCCCGGGGCCGAGGGAGCGAAGATCGGAAGAGCACACGT	2456281	1.5452419588746553	No Hit
CCGCCGAGGGCGCACCACCGGCCCGTCTCGCAGATCGGAAGAGCACACGT	2133273	1.3420382070839665	No Hit
AGCCCGTGGACGGTGTGAGGCCGGTAGATCGGAAGAGCACACGTCTGAAC	2113359	1.3295103455041921	Illumina Multiplexing PCR Primer 2.01 (100% over 25bp)
ACGATCAAAAGGGACAAGCATCAAGCACGCAGCAGATCGGAAGAGCACAC	2007035	1.2626221083540496	No Hit
ACTCGCCGAATCCCGGGGCCGAGGGAGCGAAGATCGGAAGAGCACACGTC	1924034	1.2104063285517572	No Hit
TTTGGTGACTCTAGATAACCTCGGGCCGATCGCACAGATCGGAAGAGCAC	1633735	1.0277797498258892	No Hit
AAGCTTGGGCTGCAGGTCGACCCGTGCAGATCGGAAGAGCACACGTCTGA	1534784	0.9655298537135935	Illumina Multiplexing PCR Primer 2.01 (100% over 23bp)
CCGCCGAGGGCGCACCACCGGCCCGTCTCGAGATCGGAAGAGCACACGTC	1514450	0.9527377708892923	No Hit
GTTGAACCCCATTCGTGATGGGGATCGGGGATTAGATCGGAAGAGCACAC	1405248	0.8840389891159406	No Hit
GCACTCGCCGAATCCCGGGGCCGAGGGAGCGAAGATCGGAAGAGCACACG	958134	0.6027603759604089	No Hit
ACTCGCCGAATCCCGGGGCCGAGGGAGCGAGATCGGAAGAGCACACGTCT	915225	0.5757664012427961	Illumina Multiplexing PCR Primer 2.01 (100% over 21bp)
CACTCGCCGAATCCCGGGGCCGAGGGAGCGAGATCGGAAGAGCACACGTC	846841	0.5327461498482348	No Hit
TTGGTGACTCTAGATAACCTCGGGCCGATCGCAGATCGGAAGAGCACACG	831586	0.5231492567881033	No Hit
AGCCCGTGGACGGTGTGAGGCCGGTAGCGGCCCCCGAGATCGGAAGAGCA	766687	0.48232141262491257	No Hit
AGATAACCTCGGGCCGATCGCACAGATCGGAAGAGCACACGTCTGAACTC	692679	0.4357631129464981	Illumina Multiplexing PCR Primer 2.01 (100% over 27bp)
CGTGGGGGGCCCAAGTCCTTCTGATCGAGGCCAGATCGGAAGAGCACACG	669041	0.4208924896652534	No Hit
TTGGTGACTCTAGATAACCTCGGGCCGATCAGATCGGAAGAGCACACGTC	574247	0.3612577547755709	No Hit
GTGGGGGGCCCAAGTCCTTCTGATCGAGGCCAGATCGGAAGAGCACACGT	569769	0.35844065303035505	No Hit
AAAAGGGACAAGCATCAAGCACGCAGCAGATCGGAAGAGCACACGTCTGA	510505	0.32115777723123123	Illumina Multiplexing PCR Primer 2.01 (100% over 23bp)
AGCACTCGCCGAATCCCGGGGCCGAGGGAGCGAAGATCGGAAGAGCACAC	501475	0.3154770204739066	No Hit
GGTGCGCCGCGACCGGCTCCGGGACGGCTGGGAGATCGGAAGAGCACACG	488672	0.3074226762032502	No Hit
AGCACTCGCCGAATCCCGGGGCCGAGGGAGCGAGATCGGAAGAGCACACG	465855	0.29306854254523507	No Hit
TTGGTGACTCTAGATAACCTCGGGCAGATCGGAAGAGCACACGTCTGAAC	461229	0.2901583342662335	Illumina Multiplexing PCR Primer 2.01 (100% over 25bp)
AGCCCGTGGACGGTGTGAGGCCGGTAGCGAGATCGGAAGAGCACACGTCT	439232	0.2763200611332468	Illumina Multiplexing PCR Primer 2.01 (100% over 21bp)
TGGATAACTGTGGTAATTCTAGAGCTAATACAAGATCGGAAGAGCACACG	437180	0.2750291516242734	No Hit
ACTCGCCGAATCCCGGGGCCGAGGGAGCAGATCGGAAGAGCACACGTCTG	430893	0.27107401123298874	Illumina Multiplexing PCR Primer 2.01 (100% over 22bp)
GTGGACGGTGTGAGGCCGGTAGCGGCCCCCGAGATCGGAAGAGCACACGT	430195	0.27063490068851337	No Hit
GCCGCCTGGATACCGCAGCTAGGAATAATGGAATAGATCGGAAGAGCACA	408209	0.2568035476357404	No Hit
CTCCCGGGGCTACGCCTGTCTGAGCGTCGCTTAGATCGGAAGAGCACACG	378181	0.2379129868484819	No Hit
TTTGGTGACTCTAGATAACCTCGGGCCGATCGCAGATCGGAAGAGCACAC	374999	0.23591119637209132	No Hit
ACTCTGGTGGAGGTCCGTAGCGGTCCTGACGTGCAGATCGGAAGAGCACA	370756	0.2332419327041701	No Hit
TTGGTGACTCTAGATAACCTCGGGCCGATCGCAAGATCGGAAGAGCACAC	369496	0.23244926897058993	No Hit
GAATACAAGCTTGGGCTGCAGGTCGACCCGAGATCGGAAGAGCACACGTC	364880	0.22954535167360096	No Hit
AGCACTCGCCGAATCCCGGGGCCGAGGGAGCAGATCGGAAGAGCACACGT	359940	0.22643760655940565	No Hit
CGCCGAGGGCGCACCACCGGCCCGTCTCGCAGATCGGAAGAGCACACGTC	354689	0.2231342119046203	No Hit
GTGGGGGGCCCAAGTCCTTCTGATCGAGGCAGATCGGAAGAGCACACGTC	349199	0.21968046277973519	No Hit
TTTGGTGACTCTAGATAACCTCGGGCCGATCAGATCGGAAGAGCACACGT	342806	0.21565863797911766	No Hit
TTTGGTGACTCTAGATAACCTCGGGCCGATCGCAAGATCGGAAGAGCACA	337140	0.21209416757081181	No Hit
TTTTGGTGACTCTAGATAACCTCGGGCCGATCGCACAGATCGGAAGAGCA	334786	0.21061327040505967	No Hit
GCACTCGCCGAATCCCGGGGCCGAGGGAGCGAGATCGGAAGAGCACACGT	322439	0.20284579491417512	No Hit
ACTCCCGACCCGGGGAGGTAGTGACGAAAAATAACAGATCGGAAGAGCAC	304359	0.19147169943550074	No Hit
ACCCCTCCCACGGCGCGACCGCTCAGATCGGAAGAGCACACGTCTGAACT	285432	0.17956475778036413	Illumina Multiplexing PCR Primer 2.01 (100% over 26bp)
TGGTGACTCTAGATAACCTCGGGCCGATCGCACAGATCGGAAGAGCACAC	284389	0.17890860835645608	No Hit
CCGCCGAGGGCGCACCACCGGCCCGTCTCAGATCGGAAGAGCACACGTCT	258678	0.1627338645040116	Illumina Multiplexing PCR Primer 2.01 (100% over 21bp)
CTCCCGGGGCTACGCCTGTCTGAGCGTCGCTAGATCGGAAGAGCACACGT	257689	0.16211168638297127	No Hit
AGCCCGTGGACGGTGTGAGGCCGGTAGCGGCCCCCAGATCGGAAGAGCAC	257030	0.1616971106683448	No Hit
TAGATAACCTCGGGCCGATCGCACAGATCGGAAGAGCACACGTCTGAACT	255912	0.1609937788793427	Illumina Multiplexing PCR Primer 2.01 (100% over 26bp)
ACTCGCCGAATCCCGGGGCCGAGGGAGAGATCGGAAGAGCACACGTCTGA	255142	0.16050937326437703	Illumina Multiplexing PCR Primer 2.01 (100% over 23bp)
CGTGGGGGGCCCAAGTCCTTCTGATCGAGGCAGATCGGAAGAGCACACGT	246058	0.15479464520418468	No Hit
CGGTGCGCCGCGACCGGCTCCGGGACGGCTGGGAGATCGGAAGAGCACAC	246035	0.15478017594555582	No Hit
GGACGGTGTGAGGCCGGTAGCGGCCCCCGAGATCGGAAGAGCACACGTCT	244426	0.15376795693973797	Illumina Multiplexing PCR Primer 2.01 (100% over 21bp)
AAGCTTGGGCTGCAGGTCGACCCGAGATCGGAAGAGCACACGTCTGAACT	242093	0.15230027083621211	Illumina Multiplexing PCR Primer 2.01 (100% over 26bp)
AGCACTCGCCGAATCCCGGGGCCGAGGGAGCGAGAGATCGGAAGAGCACA	239256	0.1505155192392542	No Hit
CGATCAAAAGGGACAAGCATCAAGCACGCAGATCGGAAGAGCACACGTCT	224280	0.14109414457727257	Illumina Multiplexing PCR Primer 2.01 (100% over 21bp)
GTGCGCCGCGACCGGCTCCGGGACGGCTGGGAGATCGGAAGAGCACACGT	218155	0.13724091809459116	No Hit
CGTGGACGGTGTGAGGCCGGTAGCGGCCCCCGAGATCGGAAGAGCACACG	215977	0.1358707422122597	No Hit
GCACTCGCCGAATCCCGGGGCCGAGGGAGCGAGAGATCGGAAGAGCACAC	215070	0.1353001501437222	No Hit
AGCCCGTGGACGGTGTGAGGCCGGTAGCAGATCGGAAGAGCACACGTCTG	212063	0.1334084518525511	Illumina Multiplexing PCR Primer 2.01 (100% over 22bp)
ACCGGGGCCCGCGCGGCCAACCCCCGGGACGGGGACAGATCGGAAGAGCA	206182	0.12970872533097563	No Hit
GAATACAAGCTTGGGCTGCAGGTCGACCCGTGCAGATCGGAAGAGCACAC	203331	0.12791516635920017	No Hit
TTTGGTGACTCTAGATAACCTCGGGCAGATCGGAAGAGCACACGTCTGAA	199640	0.12559316489837125	Illumina Multiplexing PCR Primer 2.01 (100% over 24bp)
CGCGGGAGACCGGGGTTCGATTCCCCGACGGGGAAGATCGGAAGAGCACA	189011	0.11890648011724125	No Hit
CCGCCGAGGGCGCACCACCGGCCCGTCTCGAGATCGGAAAAGCACACGTC	187382	0.1178816791473983	No Hit
TCCCGGGGCTACGCCTGTCTGAGCGTCGCTTAGATCGGAAGAGCACACGT	183964	0.11573142149551174	No Hit
ATGTGGCGTACGGAAGACCCGCTCCCCGGCGAGATCGGAAGAGCACACGT	183822	0.11564208955093366	No Hit
CTGGTCCGAAGGTAGTGAGTTATCTCAAAGATCGGAAGAGCACACGTCTG	177973	0.11196249417179836	Illumina Multiplexing PCR Primer 2.01 (100% over 22bp)
GTGGGGGGGCCGGGCCACCCCTCCCACGGCGCGACCGCAGATCGGAAGAG	175001	0.11009281431767114	No Hit
GCACTCGCCGAATCCCGGGGCCGAGGGAGCAGATCGGAAGAGCACACGTC	173403	0.10908751539206706	No Hit
AGTGAAAAAACGATCAGAGTAGTGGTATTTCAGATCGGAAGAGCACACGT	173160	0.1089346445291623	No Hit
ATGTGGCGTACGGAAGACCCGCTCCCCGGCGCAGATCGGAAGAGCACACG	172037	0.10822816724915393	No Hit
CGGGAGGGAGGAAGACGAACGGAAGGACAGATCGGAAGAGCACACGTCTG	170557	0.10729710191129783	Illumina Multiplexing PCR Primer 2.01 (100% over 22bp)
GTTGAACCCCATTCGTGATGGGGATCGGGGATTGAGATCGGAAGAGCACA	169073	0.10636352018063674	No Hit
GGTGACTCTAGATAACCTCGGGCCGATCGCACAGATCGGAAGAGCACACG	160309	0.10085010354484569	No Hit

Adapter Content

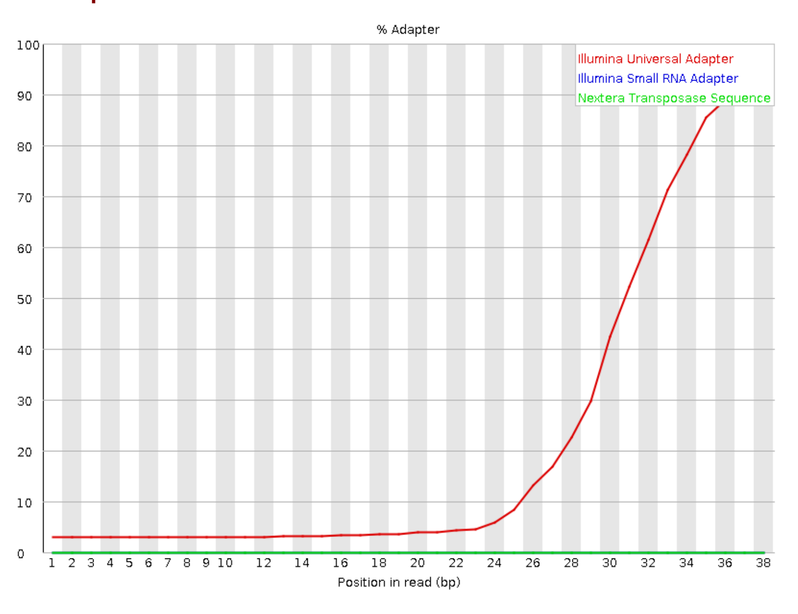

Kmer Content

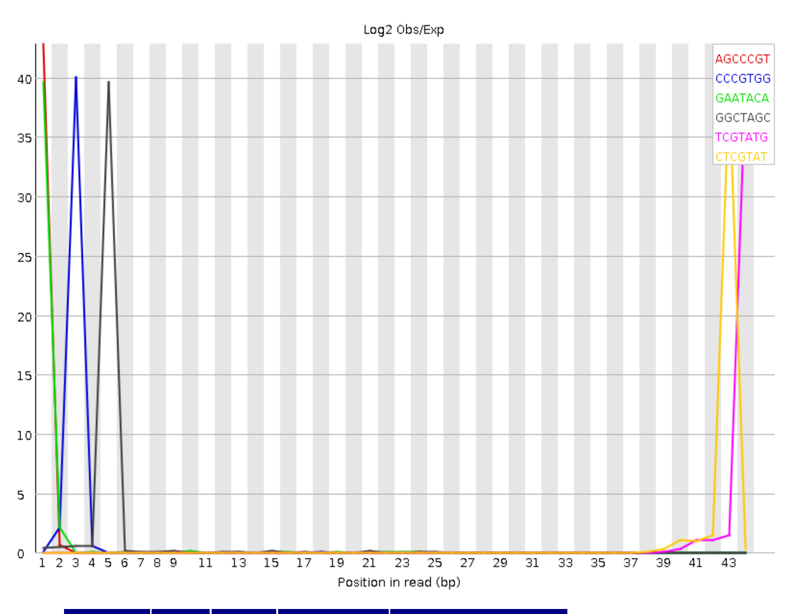

Sequence	Count	PValue	Obs/Exp Max	Max Obs/Exp Position
AGCCCGT	458350	0.0	42.801205	1
CCCGTGG	495835	0.0	40.03166	3
GAATACA	125355	0.0	39.647346	1
GGCTAGC	28265	0.0	39.621346	5
TCGTATG	559910	0.0	39.45886	44
CTCGTAT	561620	0.0	39.332058	43
TTTTGGT	90580	0.0	39.21854	1
TCTCGTA	565680	0.0	39.038307	42
GCCCGTG	504720	0.0	39.028297	2
GTACAGT	25695	0.0	38.874607	1
GTAATCT	557485	0.0	38.866055	38
AATCTCG	570490	0.0	38.66079	40
TGTAATC	562095	0.0	38.572563	37
TTGTAAT	562435	0.0	38.517956	36
ATTATAC	18395	0.0	38.382095	1
ATCTCGT	575570	0.0	38.308865	41
CCGCCGA	593100	0.0	38.3001	1
TAGCGAG	56595	0.0	38.206505	25
TATTCAC	12930	0.0	38.146706	22
CCGTGGA	517605	0.0	38.06869	4

Produced by FastQC (version 0.11.2)
