# Supplementary material for: PROTEOFORMER 2.0: Further Developments in the Ribosome Profiling-assisted Proteogenomic Hunt for New Proteoforms
Source: Mol Cell Proteomics. 2019 Apr 30;18(8 Suppl 1):S126–40. doi: 10.1074/mcp.RA118.001218 (PMC6692777; doi:10.1074/mcp.RA118.001218)
Supplement: Supplemental file S3 [file 142014_2_supp_322595_pqrw9j.pdf]

FastQC Report

Fri 10 Aug 2018
jurkat_chx.fastq

Summary

- Basic Statistics
- Per base sequence quality
- Per tile sequence quality
- Per sequence quality scores
- Per base sequence content
- Per sequence GC content
- Per base N content
- Sequence Length Distribution
- Sequence Duplication Levels
- Overrepresented sequences
- Adapter Content
- Kmer Content

Basic Statistics

Measure	Value
Filename	jurkat_chx.fastq
File type	Conventional base calls
Encoding	Sanger / Illumina 1.9
Total Sequences	388812515
Sequences flagged as poor quality	0
Sequence length	76
%GC	53

Per base sequence quality

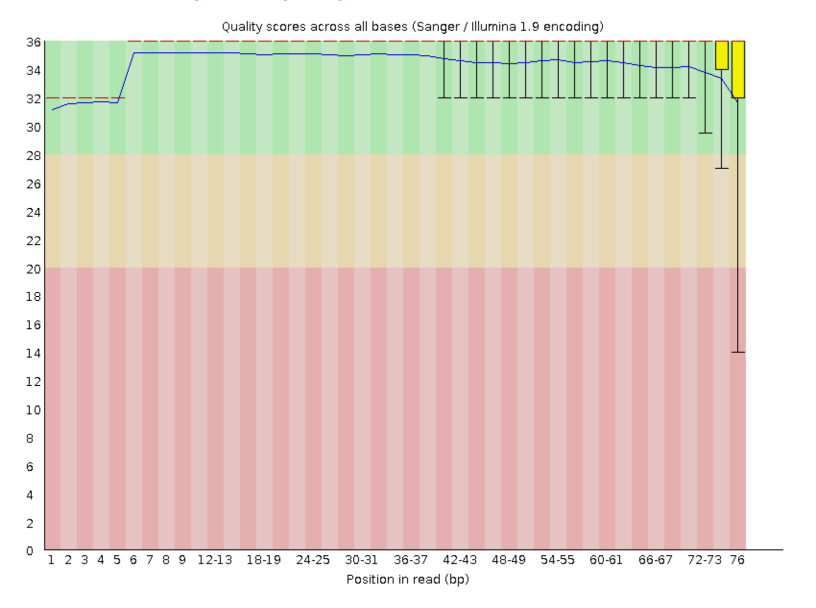

Per tile sequence quality

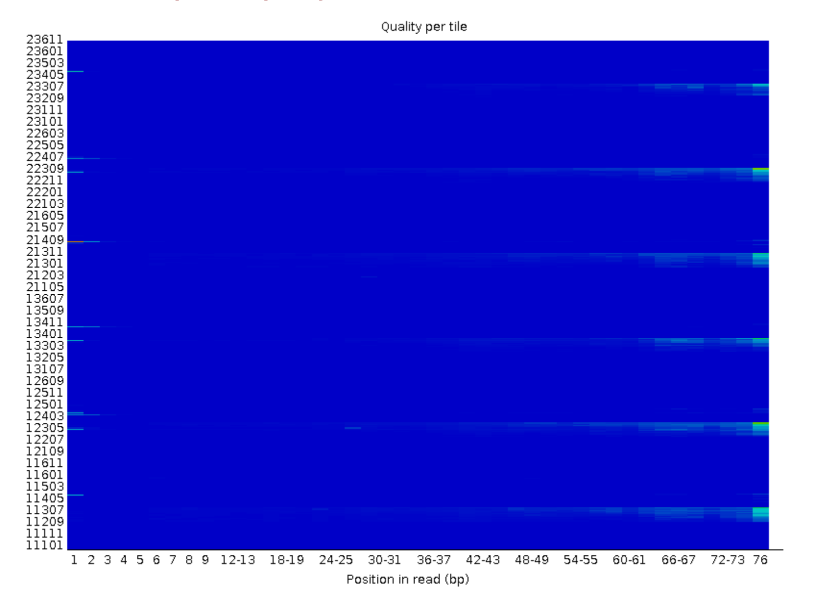

Per sequence quality scores

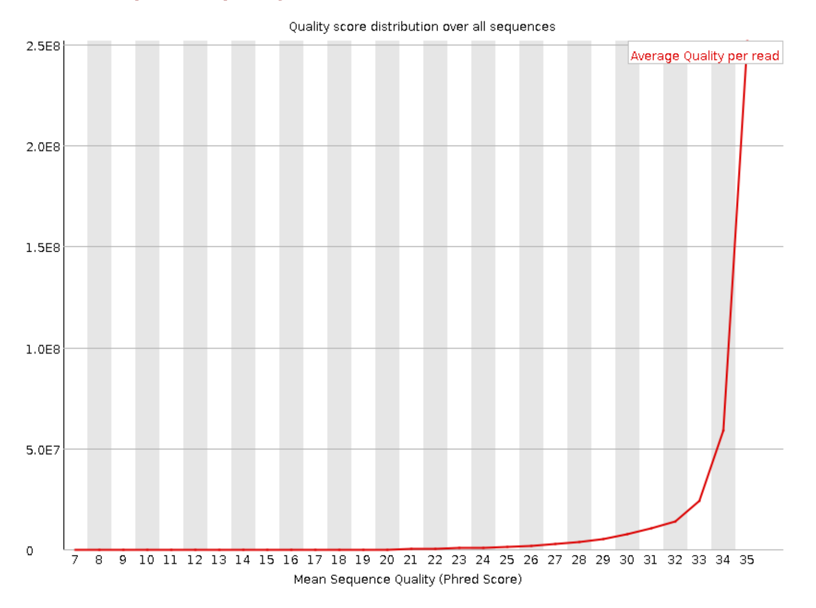

Per base sequence content

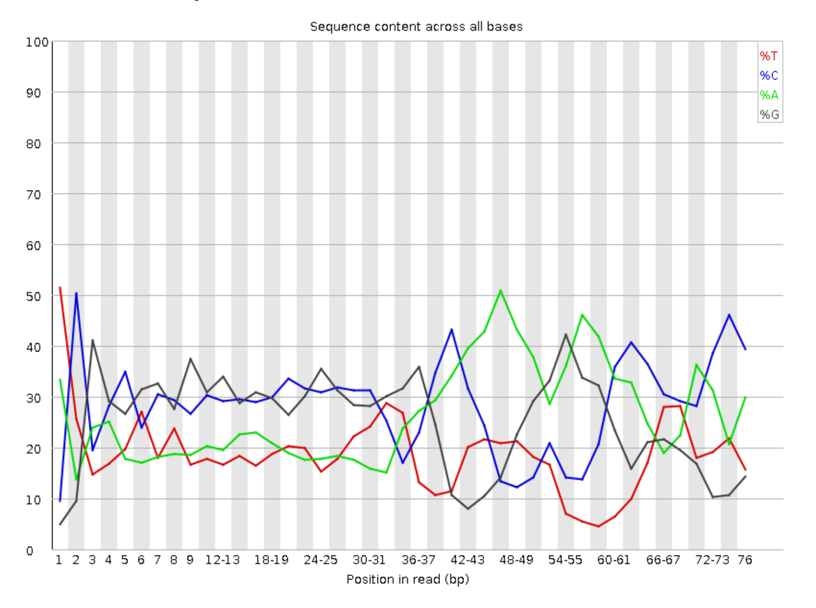

Per sequence GC content

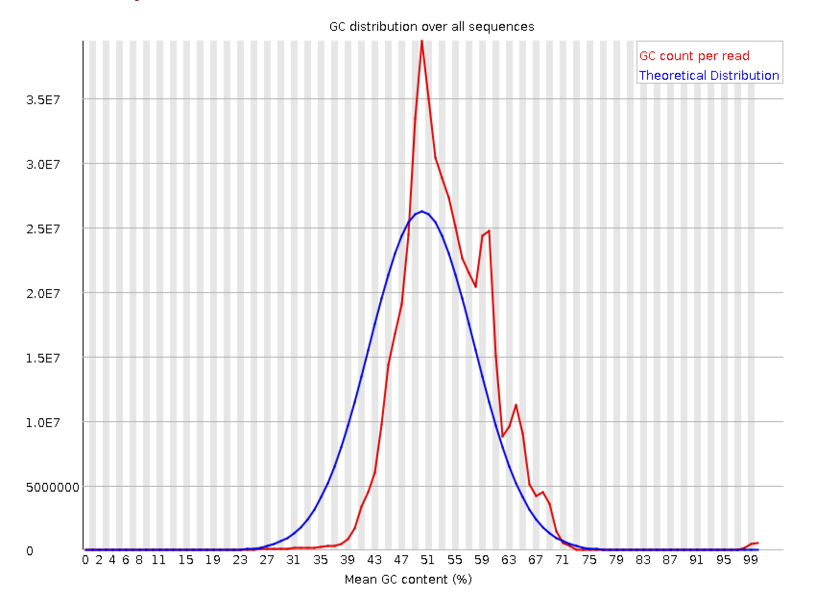

Per base N content

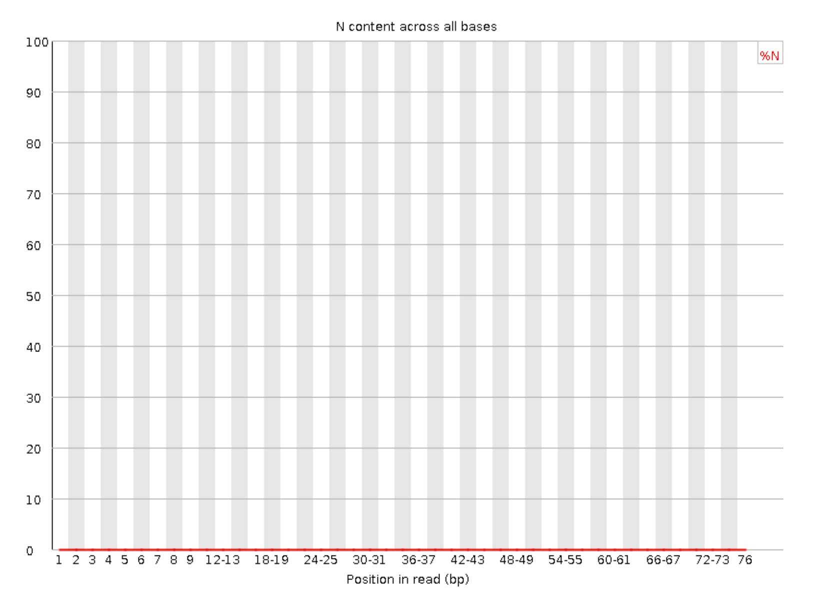

Sequence Length Distribution

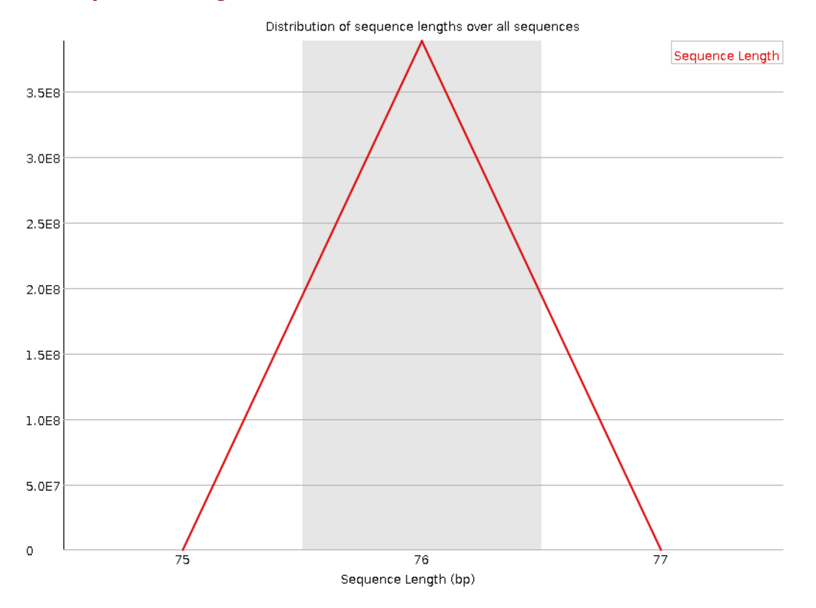

Sequence Duplication Levels

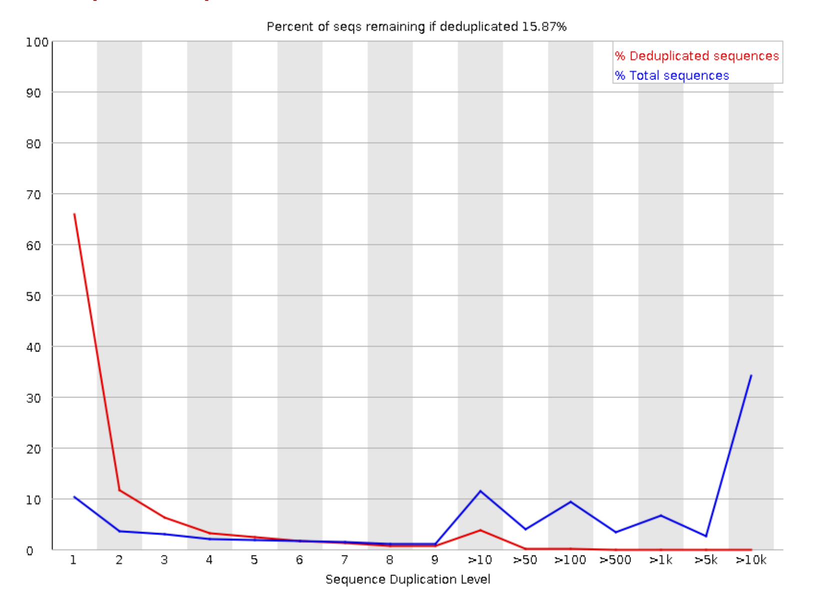

Overrepresented sequences

Sequence	Count	Percentage	Possible Source
TCGCTTCTGGCGCCAAGCGCCCGGCCCTGTAGGCACCATCAATAGATCGG	7922948	2.0377296754452465	No Hit
TCGCTTCTGGCGCCAAGCGCCCGGCCGCTGTAGGCACCATCAATAGATCG	3818970	0.9822137541020253	No Hit
TCGACTGCATAATTTGTGGTAGTGGGGGCTGTAGGCACCATCAATAGATC	3343300	0.8598745850554734	No Hit
CCGCCGAGGGCGCACCACCGGCCCGTCTCGCCTGTAGGCACCATCAATAG	3163222	0.8135597178501314	No Hit
TCGCCCCGGGCGGGTCGCGCCGTCGGGCCCGGGGGCTGTAGGCACCATCA	2885122	0.7420342423905774	No Hit
TCGTGGGGGGCCCAAGTCCTTCTGATCGAGGCCCTGTAGGCACCATCAAT	2863447	0.7364595761533036	No Hit
TCGACTGCATAATTTGTGGTAGTGGGGCTGTAGGCACCATCAATAGATCG	2854714	0.7342135064762512	No Hit
TCGCCGGCCGAGGTGGGATCCCGAGGCCTCTGTAGGCACCATCAATAGAT	2323714	0.5976438284143194	No Hit
TCGACTGCATAATTTGTGGTAGTGGGGACTGTAGGCACCATCAATAGAT	2175366	0.5594897067549381	No Hit
TGTGGGGGGGCCGGGCCACCCCTCCCACGGCGCCTGTAGGCACCATCAAT	2167007	0.557339827397274	No Hit
TCGCCGAGGGCGCACCACCGGCCCGTCTCGCCTGTAGGCACCATCAATAG	1790049	0.4603887300284046	No Hit
TCGCTTCTGGCGCCAAGCGCCCGGCCTGTAGGCACCATCAATAGATCGGA	1736508	0.4466183399472108	No Hit
TCTCGCTTCTGGCGCCAAGCGCCCGGCCCTGTAGGCACCATCAATAGATC	1609466	0.41394398017255185	No Hit
CTGTAGGCACCATCAATAGATCGGAAGAGCACACGTCTGAACTCCAGTCA	1514545	0.3895309285504866	Illumina Multiplexing PCR Primer 2.01 (100% over 33bp)
TCGCCGGCCGAGGTGGGATCCCGAGGCCCTGTAGGCACCATCAATAGATC	1475944	0.379603007377476	No Hit
ACGATGCCGACCGGCGATGCGGCGGCGTTCTGTAGGCACCATCAATAGAT	1383058	0.3557133442579645	No Hit
ACGATGCCGACCGGCGATGCGGCGGCGTTACTGTAGGCACCATCAATAGA	1353352	0.3480731580875168	No Hit
TCCGCCGAGGGCGCACCACCGGCCCGTCTCGCCTGTAGGCACCATCAATA	1348307	0.34677561754924474	No Hit
TGTGGGGGGGCCGGGCCACCCCTCCCACGGCGCTGTAGGCACCATCAATA	1282183	0.3297689633267077	No Hit
TTTTTTTCACTGACCCGGTGAGGCGGGGCTGTAGGCACCATCAATAGATC	1142492	0.29384136464846045	No Hit
ACCCCTCCCACGGCGCCTGTAGGCACCATCAATAGATCGGAAGAGCACAC	1048951	0.26978323987333586	No Hit
CTCGCTTCTGGCGCCAAGCGCCCGGCCCTGTAGGCACCATCAATAGATCG	990848	0.2548395336502993	No Hit
ATCCCACCGCTGCCACCACTGTAGGCACCATCAATAGATCGGAAGAGCAC	932440	0.23981738345022152	No Hit
ATCCCACTTCTGACACCACTGTAGGCACCATCAATAGATCGGAAGAGCAC	799872	0.20572177312759596	No Hit
CGTGGGGGGCCCAAGTCCTTCTGATCGAGGCCCTGTAGGCACCATCAATA	747604	0.19227879020303654	No Hit
CCGCCGAGGGCGCACCACCGGCCCGTCTCGCTGTAGGCACCATCAATAGA	736363	0.18938767956067465	No Hit
GTGGGGGGGCCGGGCCACCCCTCCCACGGCGCCTGTAGGCACCATCAATA	733694	0.1887012304632221	No Hit
TCGCCGGCCGAGGTGGGATCCCGAGGCCTCCTGTAGGCACCATCAATAGA	708202	0.18214485714277998	No Hit
TCAGTGCGCCCCGGGCGGGTCGCGCCGTCGGGCCCGGGGCTGTAGGCACC	706480	0.18170197016420625	No Hit
TCTCGCTTCTGGCGCCAAGCGCCCGGCCTGTAGGCACCATCAATAGATCG	631139	0.1623247646748202	No Hit
TTTTTTCACTGACCCGGTGAGGCGGGGCTGTAGGCACCATCAATAGATCG	629930	0.16201381789369615	No Hit
ATCCCACTCCTGACACCACTGTAGGCACCATCAATAGATCGGAAGAGCAC	621572	0.1598641957293993	No Hit
ATCCCATCCTCGTCGCCACTGTAGGCACCATCAATAGATCGGAAGAGCAC	569447	0.14645799145637067	No Hit
TGGGCGCGCGCCGCGGCTGGACGAGGCGCCGCTGTAGGCACCATCAATAG	559081	0.1437919250104385	No Hit
ATGTACACGGAGTCGACCCGCAACGCGACTGTAGGCACCATCAATAGATC	555159	0.14278321262369859	No Hit
TTCTCGCTTCTGGCGCCAAGCGCCCGGCCCTGTAGGCACCATCAATAGAT	533066	0.13710103955887326	No Hit
TCTCGCTTCTGGCGCCAAGCGCCCGGCCGCTGTAGGCACCATCAATAGAT	512245	0.1317460164573149	No Hit
CTCGACTGCATAATTTGTGGTAGTGGGGGCTGTAGGCACCATCAATAGAT	499959	0.12858613874607405	No Hit
TGTGGGGGGGCCGGGCCACCCCTCCCACGGCGCGACCGCTCTGTAGGCAC	497011	0.12782793269913137	No Hit
TCGAGGGCGCACCACCGGCCCGTCTCGCCTGTAGGCACCATCAATAGATC	484515	0.12461404438074736	No Hit
ATGTACACGGAGTCGAGCTCAACCCGCAACGCGACTGTAGGCACCATCAA	481602	0.12386484010166185	No Hit
ATAAACGATGCCGACCGGCGATGCGGCGGCGTTCTGTAGGCACCATCAAT	479331	0.12328075396441393	No Hit
TGGGCGCGCGCCGCGGCTGGACGAGGCGCCGCCTGTAGGCACCATCAATA	468573	0.12051386771848123	No Hit
ACCCCACTCCTGGTACCACTGTAGGCACCATCAATAGATCGGAAGAGCAC	466932	0.12009181340266273	No Hit
TTGGGGGGGCCGGGCCACCCCTCCCACGGCGCCTGTAGGCACCATCAATA	465096	0.1196196063802113	No Hit
TCGCTTCTGGCGCCAAGCGCCCGGCCGCCTGTAGGCACCATCAATAGATC	456749	0.11747281334295528	No Hit
GTGGGGGGGCCGGGCCACCCCTCCCACGGCGCTGTAGGCACCATCAATAG	452176	0.11629666807407164	No Hit
TCTCCCGGGGCTACGCCTGTCTGAGCGTCGCTCTGTAGGCACCATCAATA	448218	0.11527869672610719	No Hit
ACAAGCTTGGGCTGCAGGTCGACCCGTGCCTGTAGGCACCATCAATAGAT	435164	0.11192129450874286	No Hit
TGGGGGGGGCCGGGCCACCCCTCCCACGGCGCCTGTAGGCACCATCAATAG	426281	0.10963664582658818	No Hit
CAGTGCGCCCCGGGCGGGTCGCGCCGTCGGGCCCGGGGCTGTAGGCACCA	414252	0.10654286681075582	No Hit
TCAGTGCGCCCCGGGCGGGTCGCGCCGTCGGGCCCGGGGGCTGTAGGCAC	406699	0.10460028530717433	No Hit
GCGCTTCTGGCGCCAAGCGCCCGGCCCTGTAGGCACCATCAATAGATCGG	402856	0.10361189119645493	No Hit
TTCGCTTCTGGCGCCAAGCGCCCGGCCCTGTAGGCACCATCAATAGATCG	398032	0.10237119039236686	No Hit
AACGATGCCGACCGGCGATGCGGCGGCGTTCTGTAGGCACCATCAATAGA	391376	0.10065931133929679	No Hit

Adapter Content

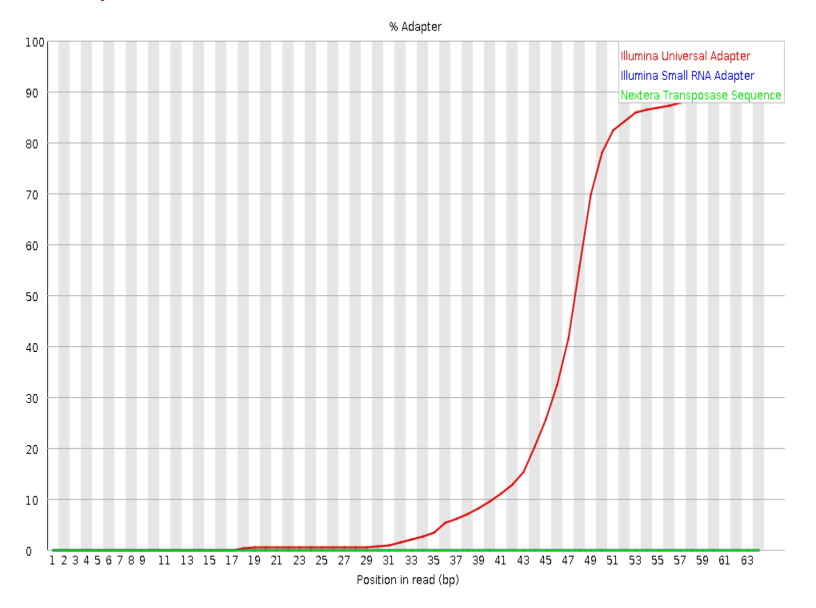

Kmer Content

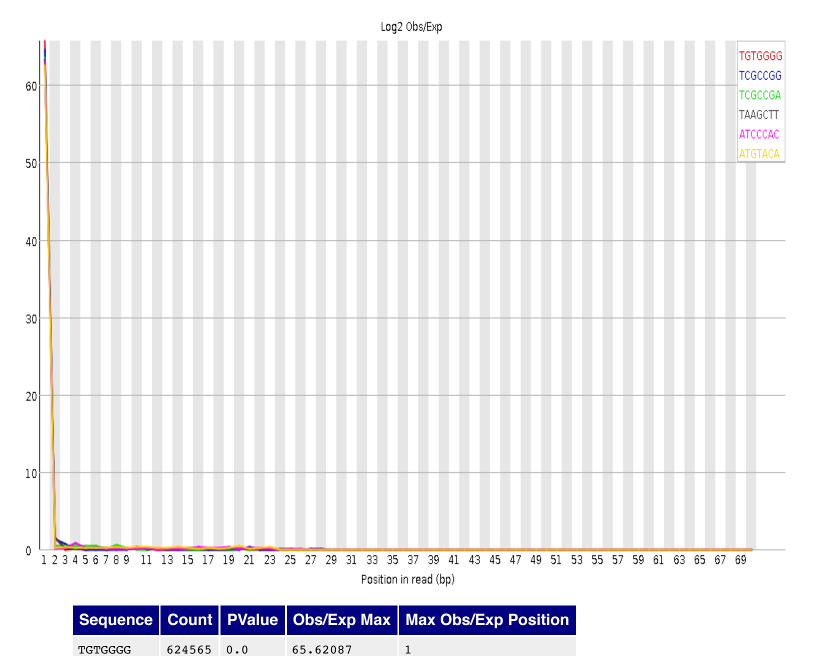

Sequence	Count	PValue	Obs/Exp Max	Max Obs/Exp Position
TGTGGGG	624565	0.0	65.62087	1
TCGCCGG	655100	0.0	64.59275	1
TCGCCGA	315085	0.0	63.509754	1
TAAGCTT	112160	0.0	63.26144	1
ATCCCAC	398715	0.0	63.0085	1
ATGTACA	170575	0.0	62.56173	1
TGTACAC	171295	0.0	61.695644	2
TTGGGGG	229240	0.0	60.8116	1
TTGGGCG	102930	0.0	60.59643	1
TCGTGGG	516245	0.0	60.462112	1
TACACGG	177535	0.0	59.757473	4
ACCCCGT	244590	0.0	59.39103	12
TGAATAC	164600	0.0	59.0104	1
CCCACTT	139300	0.0	59.001675	3
ACGCTCC	247665	0.0	58.77864	1
GTACACG	181135	0.0	58.13455	3
TGACACC	242565	0.0	58.121563	11
TTCGCTT	106905	0.0	57.910954	1
CTGACAC	248320	0.0	57.574	10
TCCCACT	248200	0.0	57.50503	2

Produced by FastQC (version 0.11.2)
