# Supplementary material for: PROTEOFORMER 2.0: Further Developments in the Ribosome Profiling-assisted Proteogenomic Hunt for New Proteoforms
Source: Mol Cell Proteomics. 2019 Apr 30;18(8 Suppl 1):S126–40. doi: 10.1074/mcp.RA118.001218 (PMC6692777; doi:10.1074/mcp.RA118.001218)
Supplement: Supplemental file S4 [file 142014_2_supp_322596_pqrw9j.pdf]

Fri 10 Aug 2018
jurkat_ltm.fastq

FastQC Report

Summary

- Basic Statistics
- Per base sequence quality
- Per tile sequence quality
- Per sequence quality scores
- Per base sequence content
- Per sequence GC content
- Per base N content
- Sequence Length Distribution
- Sequence Duplication Levels
- Overrepresented sequences
- Adapter Content
- Kmer Content

Basic Statistics

Measure	Value
Filename	jurkat_ltm.fastq
File type	Conventional base calls
Encoding	Sanger / Illumina 1.9
Total Sequences	378053973
Sequences flagged as poor quality	0
Sequence length	76
%GC	52

Per base sequence quality

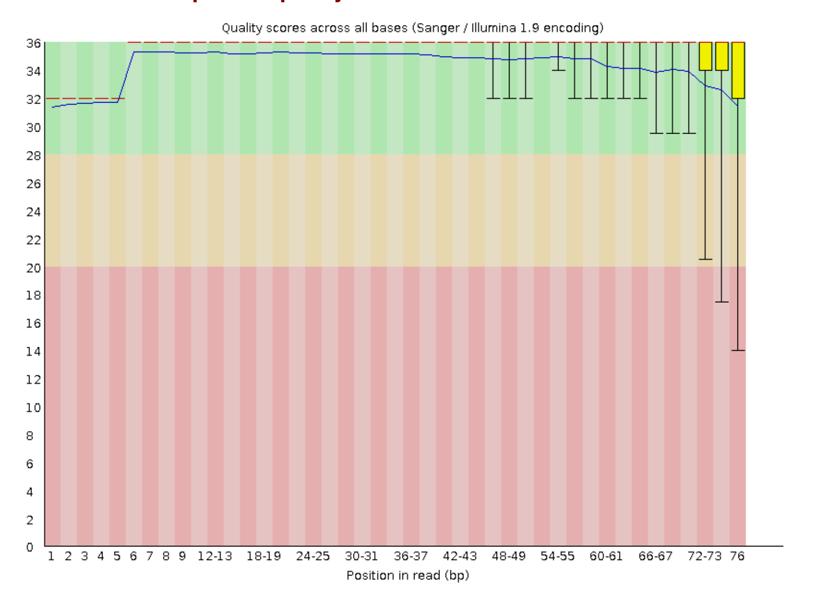

Per tile sequence quality

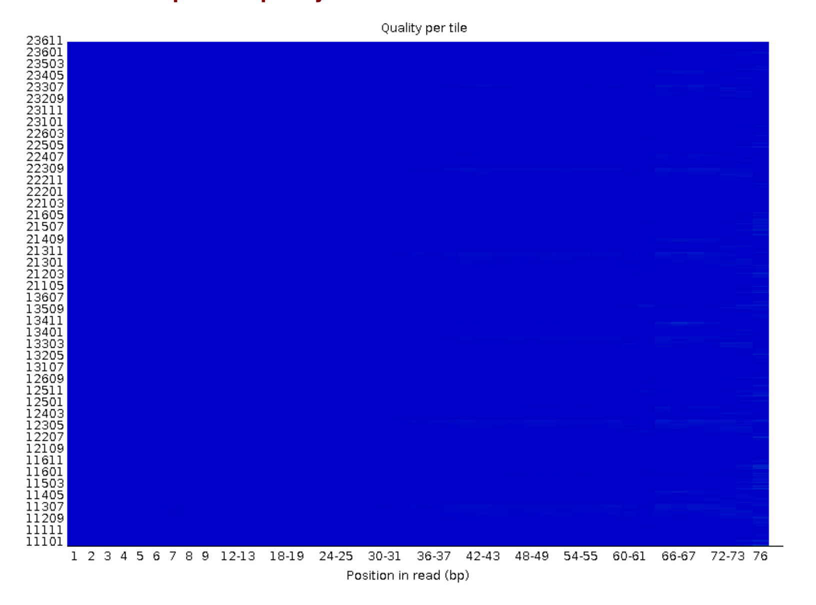

Per sequence quality scores

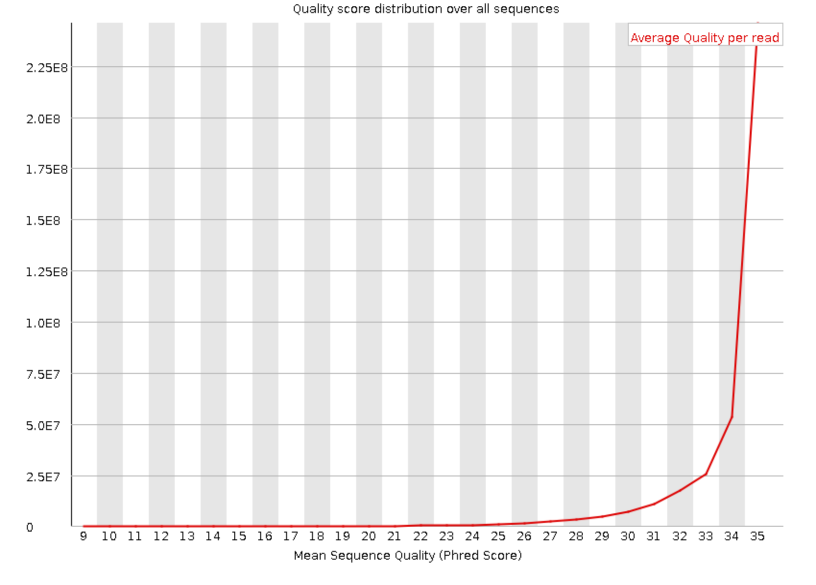

Per base sequence content

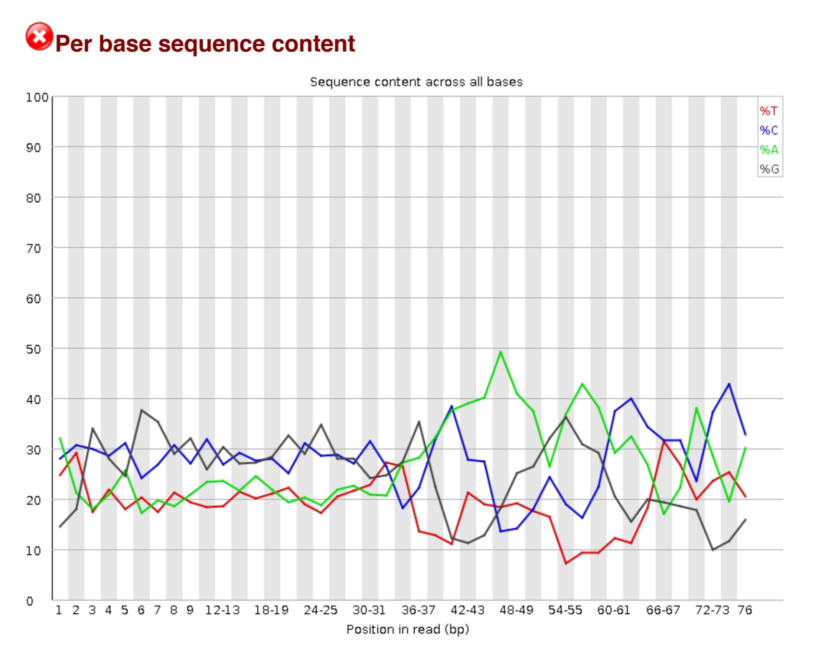

Per sequence GC content

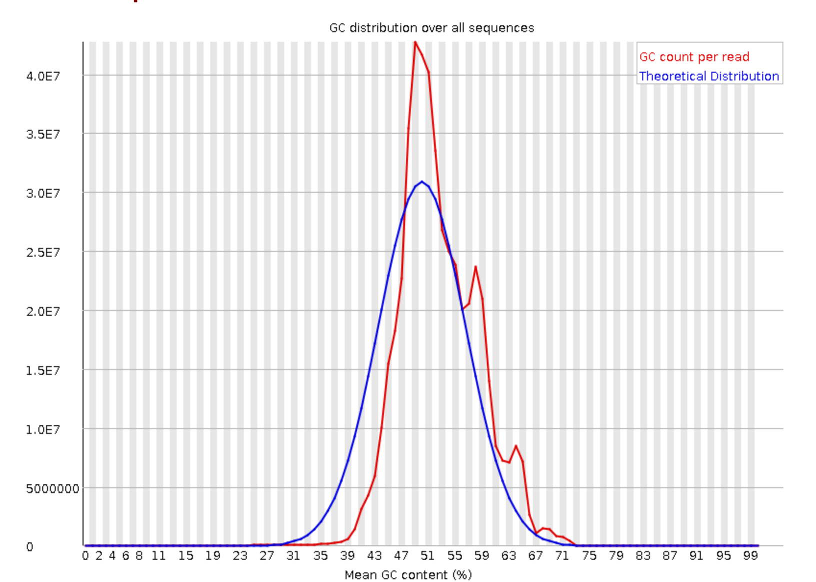

Per base N content

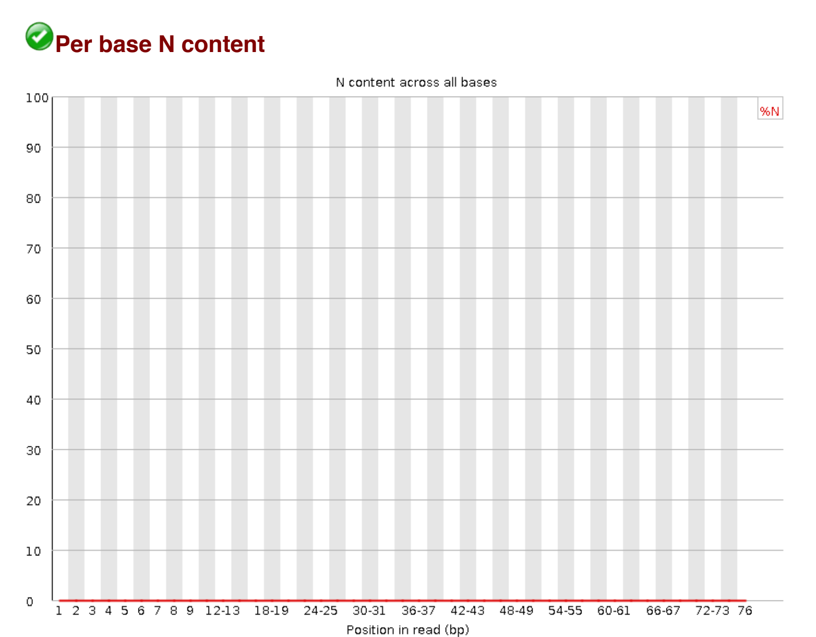

Sequence Length Distribution

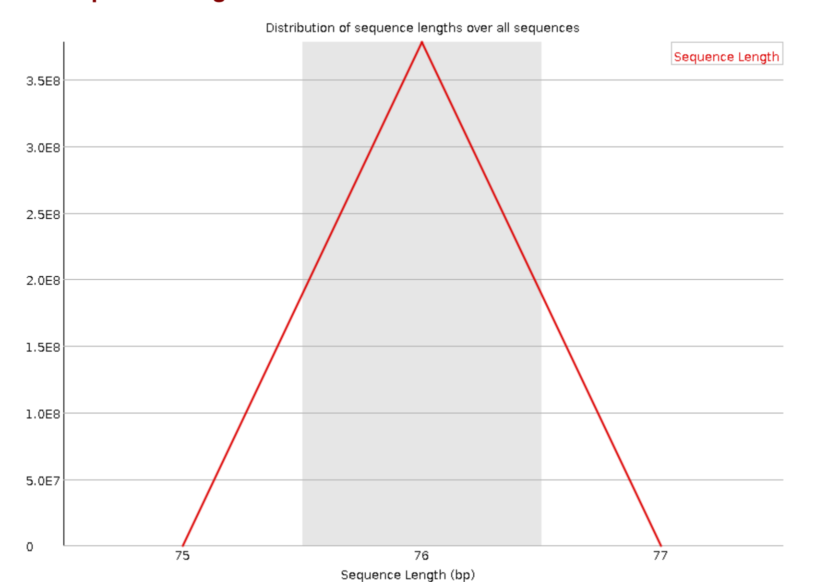

Sequence Duplication Levels

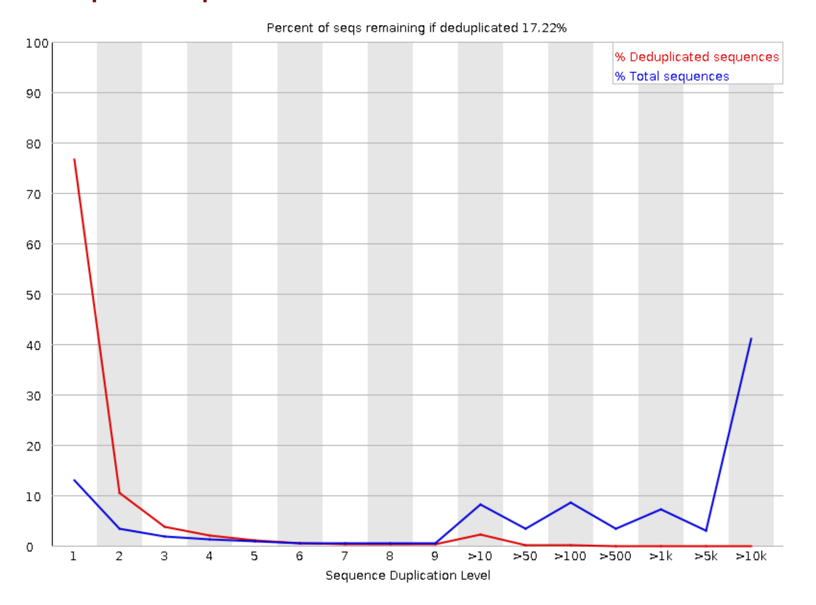

Overrepresented sequences

Sequence	Count	Percentage	Possible Source
CTGTAGGCACCATCAATAGATCGGAAGAGCACACGTCTGAACTCCAGTCA	10588610	2.8008196596838832	Illumina Multiplexing PCR Primer 2.01 (100% over 33bp)
CCGCCGAGGGCGCACCACCGGCCCGTCTCGCCTGTAGGCACCATCAATAG	7350290	1.944243553816587	No Hit
ACGATGCCGACCGGCGATGCGGCGGCGTTCTGTAGGCACCATCAATAGAT	2176818	0.5757955623971184	No Hit
AACGATGCCGACCGGCGATGCGGCGGCGTTCTGTAGGCACCATCAATAGA	2097414	0.5547922121691339	No Hit
ACGATGCCGACCGGCGATGCGGCGGCGTTACTGTAGGCACCATCAATAGA	1842720	0.48742246652702154	No Hit
CCGCCGAGGGCGCACCACCGGCCCGTCTCGCTGTAGGCACCATCAATAGA	1627833	0.43058216981097563	No Hit
GCGCAGCGGAAGCGTGCTGGGCCTGTAGGCACCATCAATAGATCGGAAGA	1572530	0.41595383524775176	No Hit
CGCGACCTCAGATCAGACGTGGCGCTGTAGGCACCATCAATAGATCGGAA	1559287	0.41245089626395753	No Hit
AACGATGCCGACCGGCGATGCGGCGGCGTTACTGTAGGCACCATCAATAG	1516999	0.4012651918354525	No Hit
AACCATCCTCTGCTACCACTGTAGGCACCATCAATAGATCGGAAGAGCAC	1478598	0.3911076474786842	No Hit
CTCGCTTCTGGCGCCAAGCGCCCGGCCCTGTAGGCACCATCAATAGATCG	1380304	0.36510765620230634	No Hit
CGAGGGCGCACCACCGGCCCGTCTCGCCTGTAGGCACCATCAATAGATCG	1368816	0.36206893664889483	No Hit
CGTGGGGGGCCCAAGTCCTTCTGATCGAGGCCCTGTAGGCACCATCAATA	1345673	0.35594732395524914	No Hit
CCTCCCGGGGCTACGCCTGTCTGAGCGTCGCTCTGTAGGCACCATCAATA	1334404	0.3529665326384495	No Hit
ACGCGACCTCAGATCAGACGTGGCGCTGTAGGCACCATCAATAGATCGGA	1322042	0.3496966291635824	No Hit
AACCATCCTCTGCCTGTAGGCACCATCAATAGATCGGAAGAGCACACGTC	1272139	0.33649666207845935	No Hit
TCGCTTCTGGCGCCAAGCGCCCGGCCCTGTAGGCACCATCAATAGATCGG	1268959	0.33565551234135554	No Hit
GTGGGGGGGCCGGGCCACCCCTCCCACGGCGCTGTAGGCACCATCAATAG	1267042	0.33514844188662973	No Hit
CTCGACTGCATAATTTGTGGTAGTGGGGGCTGTAGGCACCATCAATAGAT	1228477	0.3249475174805265	No Hit
TCGACTGCATAATTTGTGGTAGTGGGGGCTGTAGGCACCATCAATAGATC	1152910	0.30495910169948137	No Hit
GTGGGGGGGCCGGGCCACCCCTCCCACGGCGCCTGTAGGCACCATCAATA	1151064	0.3044708116319677	No Hit
TCGACTGCATAATTTGTGGTAGTGGGGCTGTAGGCACCATCAATAGATCG	1107206	0.2928698225848297	No Hit
ATCCCACTTCTGACACCACTGTAGGCACCATCAATAGATCGGAAGAGCAC	1095795	0.289851470493606	No Hit
CCTGCGGAAGGATCCTGTAGGCACCATCAATAGATCGGAAGAGCACACGT	1046321	0.27676497926924315	No Hit
TCGACTGCATAATTTGTGGTAGTGGGGGACTGTAGGCACCATCAATAGAT	927819	0.2454197194748169	No Hit
TTTTTTTCACTGACCCGGTGAGGCGGGGCTGTAGGCACCATCAATAGATC	900209	0.23811652946178669	No Hit
ATCCCACTCCTGACACCACTGTAGGCACCATCAATAGATCGGAAGAGCAC	891082	0.23570232391130036	No Hit
CCTGCGGAAGGATCATTCTGTAGGCACCATCAATAGATCGGAAGAGCACA	792728	0.20968646188516576	No Hit
GCGCAGCGGAAGCGTGCTGGGCTGTAGGCACCATCAATAGATCGGAAGAG	746661	0.19750116473448623	No Hit
ATCCCACCGCTGCCACCACTGTAGGCACCATCAATAGATCGGAAGAGCAC	734201	0.19420533903501655	No Hit
AAACGATGCCGACCGGCGATGCGGCGGCGTTCTGTAGGCACCATCAATAG	706115	0.18677624107391672	No Hit
CTCCCGGGGCTACGCCTGTCTGAGCGTCGCTCTGTAGGCACCATCAATAG	705618	0.18664477836343227	No Hit
CCTGCGGAAGGATCATTACTGTAGGCACCATCAATAGATCGGAAGAGCAC	690417	0.18262392391257848	No Hit
CAGTGCGCCCCGGGCGGGTCGCGCCGTCGGGCCCGGGGGCTGTAGGCACC	687348	0.18181213506252453	No Hit
GTGGGGGGCCCAAGTCCTTCTGATCGAGGCCCTGTAGGCACCATCAATAG	657237	0.17384739929713686	No Hit
CTCGACTGCATAATTTGTGGTAGTGGGGGACTGTAGGCACCATCAATAGA	645757	0.17081079584369294	No Hit
ATCCCATCCTCGTCGCCACTGTAGGCACCATCAATAGATCGGAAGAGCAC	631052	0.16692113959082766	No Hit
CCTGCGGAAGGATCTGTAGGCACCATCAATAGATCGGAAGAGCACACGTC	629871	0.16660875033311712	No Hit
CGATGCCGACCGGCGATGCGGCGGCGTTACTGTAGGCACCATCAATAGAT	628544	0.16625774225099862	No Hit
CTCGCTTCTGGCGCCAAGCGCCCGGCCTGTAGGCACCATCAATAGATCGG	616219	0.16299762573848153	No Hit
CGCCGAGGGCGCACCACCGGCCCGTCTCGCCTGTAGGCACCATCAATAGA	610073	0.16137193193840604	No Hit
AACGATGCCGACCGGCGATGCGGCGGCGTCTGTAGGCACCATCAATAGAT	605418	0.16014062626978398	No Hit
ACCCCACTCCTGGTACCACTGTAGGCACCATCAATAGATCGGAAGAGCAC	584504	0.15460861192959874	No Hit
AAACGATGCCGACCGGCGATGCGGCGGCGTTACTGTAGGCACCATCAATA	578863	0.1531164969399753	No Hit
AAATGGATTTTTGGAGCAGGGACTGTAGGCACCATCAATAGATCGGAAGA	567595	0.1501359701356716	No Hit
ACGATGCCGACCGGCGATGCGGCGGCGTCTGTAGGCACCATCAATAGATC	566094	0.14973893687925877	No Hit
CGATGCCGACCGGCGATGCGGCGGCGTTCTGTAGGCACCATCAATAGATC	552430	0.14612463813467186	No Hit
ATCCGAGTCACGGCCTGTAGGCACCATCAATAGATCGGAAGAGCACACGT	525944	0.13911876016708336	No Hit
TTTTTCACTGACCCGGTGAGGCGGGGCTGTAGGCACCATCAATAGATCGG	521721	0.1380017238967093	No Hit
AACCATCCTCTGCTCTGTAGGCACCATCAATAGATCGGAAGAGCACACGT	521229	0.1378715837487046	No Hit
TCGCTTCTGGCGCCAAGCGCCCGGCCGCTGTAGGCACCATCAATAGATCG	519915	0.13752401432903338	No Hit
GTGGGGGGGCCGGGCCACCCCTCCCACGGCGCGACCGCTCTGTAGGCACC	517471	0.1368775457889448	No Hit
TCCGCCGAGGGCGCACCACCGGCCCGTCTCGCCTGTAGGCACCATCAATA	513570	0.13584568254226492	No Hit
CCGAGGGCGCACCACCGGCCCGTCTCGCCTGTAGGCACCATCAATAGATC	497685	0.13164390154418507	No Hit
GCGCAGCGGAAGCGTGCTGGGCCCTGTAGGCACCATCAATAGATCGGAAG	477025	0.12617907337796977	No Hit
AGGGGAGATACCATGATCACGAAGGTGGTTCTGTAGGCACCATCAATAGA	472218	0.1249075618099641	No Hit
CTGTAGGCACCATCAATAGATTGGAAGAGCACACGTCTGAACTCCAGTCA	450815	0.11924620085926196	Illumina Multiplexing PCR Primer 2.01 (96% over 33bp)
GTGGGGGGGCCGGGCCACCCCTCCCACGGCGCGACCGCTCCTGTAGGCAC	448765	0.11870395024257555	No Hit
AACGATGCCGACCGGCGATGCGGCGGCGCTGTAGGCACCATCAATAGATC	443761	0.1173803297128688	No Hit
CTCGACTGCATAATTTGTGGTAGTGGGGCTGTAGGCACCATCAATAGATC	431618	0.11416835447461361	No Hit
ATGTACACGGAGTCGAGCTCAACCCGCAACGCGACTGTAGGCACCATCAA	407222	0.10771530762354929	No Hit
TGTAGGCACCATCAATAGATCGGAAGAGCACACGTCTGAACTCCAGTCAC	405476	0.10725346880563003	Illumina Multiplexing PCR Primer 2.01 (100% over 34bp)
GAGGGCGCACCACCGGCCCGTCTCGCCTGTAGGCACCATCAATAGATCGG	400226	0.10586477820192092	No Hit
CTGTAGGCACCATTAATAGATCGGAAGAGCACACGTCTGAACTCCAGTCA	392536	0.103830677108107	Illumina Multiplexing PCR Primer 2.01 (100% over 33bp)
CGTTTTTTCACTGACCCGGTGAGGCGGGGCTGTAGGCACCATCAATAGAT	390806	0.10337307049012284	No Hit
CCTCCCGGGGCTACGCCTGTCTGAGCGTCGCTTCTGTAGGCACCATCAAT	387449	0.10248510204123684	No Hit
ATCCTGCCGACTACGCCACTGTAGGCACCATCAATAGATCGGAAGAGCAC	387405	0.1024734634914153	No Hit
ATGTACACGGAGTCGACCCGCAACGCGACTGTAGGCACCATCAATAGATC	379647	0.10042137554787713	No Hit

Adapter Content

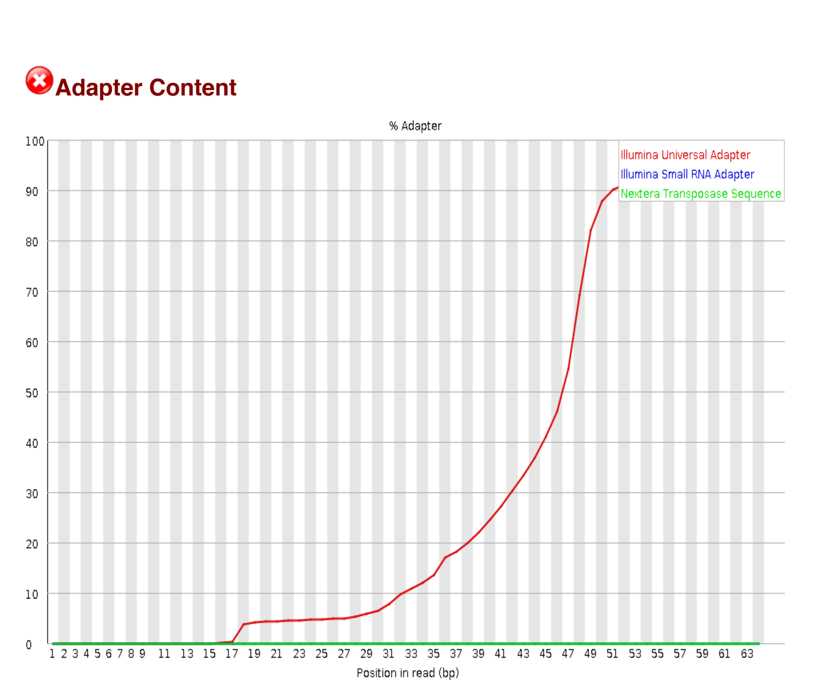

Kmer Content

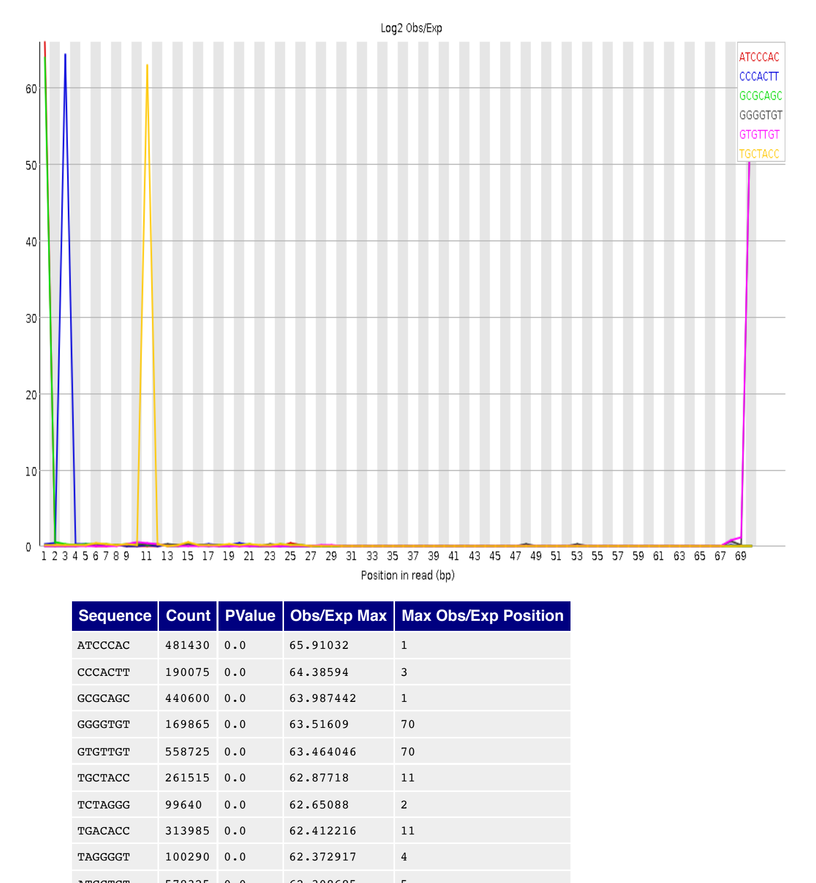

Sequence	Count	PValue	Obs/Exp Max	Max Obs/Exp Position
ATCCCAC	481430	0.0	65.91032	1
CCCACTT	190075	0.0	64.38594	3
GCGCAGC	440600	0.0	63.987442	1
GGGGTGT	169865	0.0	63.51609	70
GTGTTGT	558725	0.0	63.464046	70
TGCTACC	261515	0.0	62.87718	11
TCTAGGG	99640	0.0	62.65088	2
TGACACC	313985	0.0	62.412216	11
TAGGGGT	100290	0.0	62.372917	4
ATCCTCT	578325	0.0	62.308685	5
CGGGGTG	180950	0.0	62.220688	69
AGTCACG	129645	0.0	62.13122	6
TCCCACT	342980	0.0	62.004993	2
TCTGCTA	281110	0.0	61.97206	9
TGCCGTG	799675	0.0	61.96389	66
CCGAGTC	134415	0.0	61.84839	3
GCTCGAA	179900	0.0	61.844402	6
GTCACGG	117320	0.0	61.837925	7
CGTGTTG	580730	0.0	61.8265	69
CTGACAC	332400	0.0	61.62826	10

Produced by FastQC (version 0.11.2)
