# Supplementary material for: PROTEOFORMER 2.0: Further Developments in the Ribosome Profiling-assisted Proteogenomic Hunt for New Proteoforms
Source: Mol Cell Proteomics. 2019 Apr 30;18(8 Suppl 1):S126–40. doi: 10.1074/mcp.RA118.001218 (PMC6692777; doi:10.1074/mcp.RA118.001218)
Supplement: Supplemental file S5 [file 142014_2_supp_322597_pqrw9k.pdf]

FastQC Report

Fri 27 Apr 2018
untreat.sam

Summary

- Basic Statistics
- Per base sequence quality
- Per sequence quality scores
- Per base sequence content
- Per sequence GC content
- Per base N content
- Sequence Length Distribution
- Sequence Duplication Levels
- Overrepresented sequences
- Adapter Content
- Kmer Content

Basic Statistics

Measure	Value
Filename	untreat.sam
File type	Conventional base calls
Encoding	Sanger / Illumina 1.9
Total Sequences	51660913
Sequences flagged as poor quality	0
Sequence length	20-50
%GC	52

Per base sequence quality

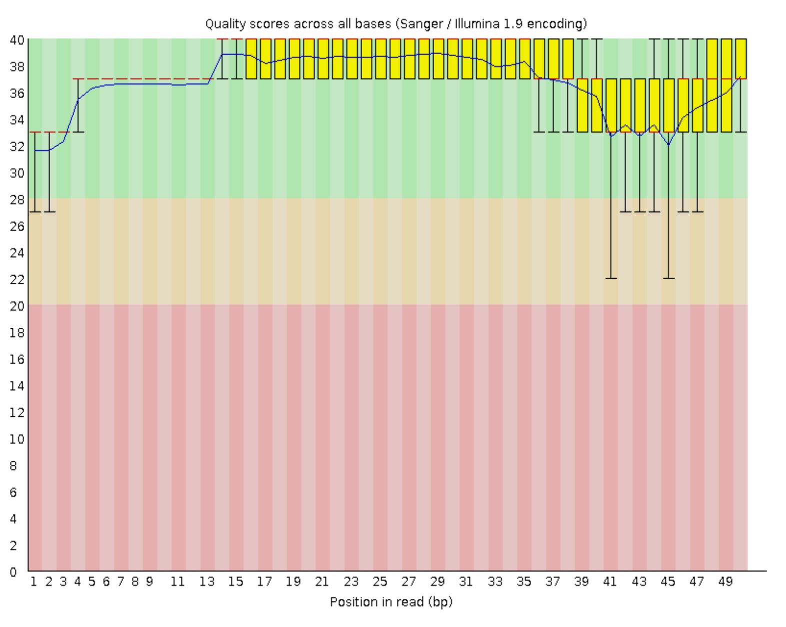

Per sequence quality scores

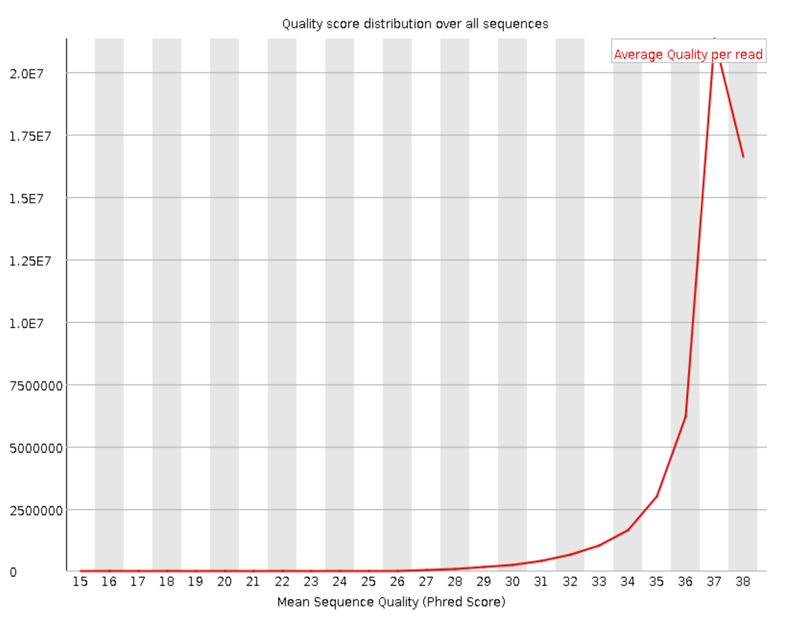

Per base sequence content

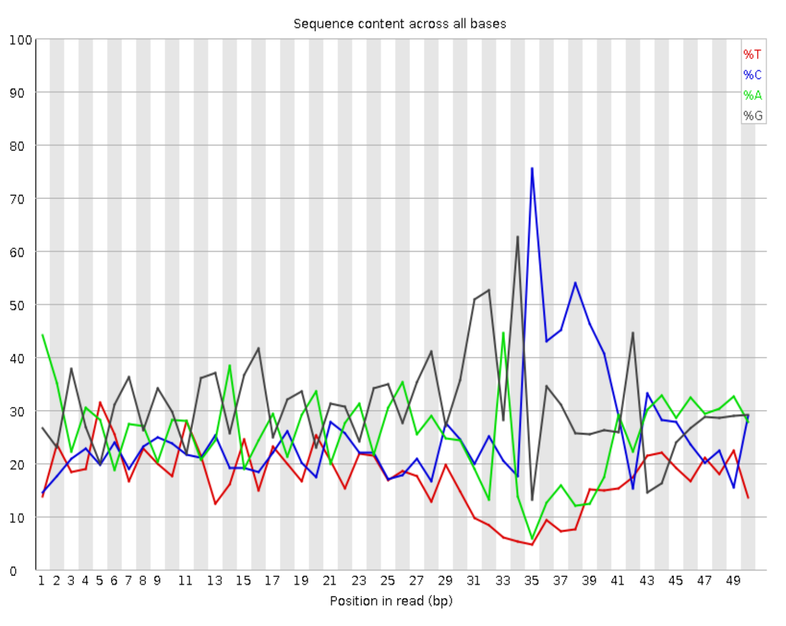

Per sequence GC content

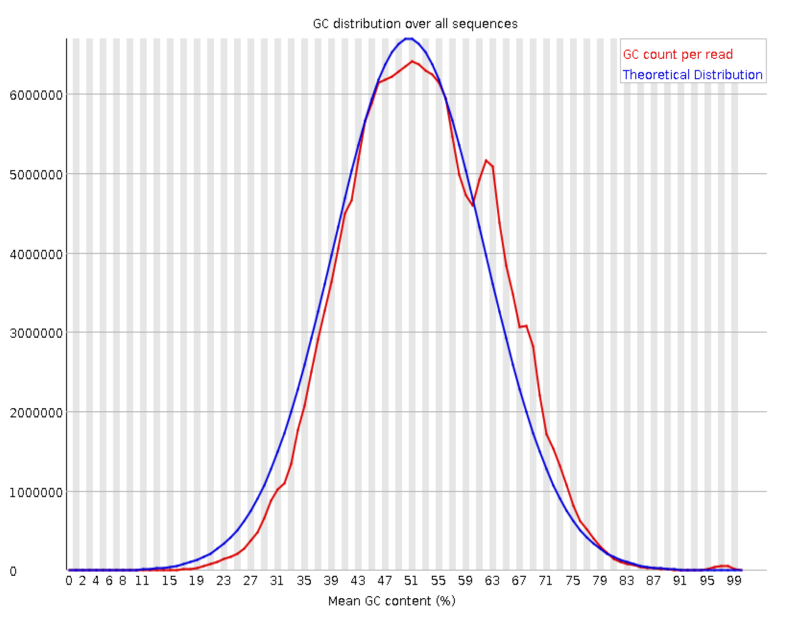

Per base N content

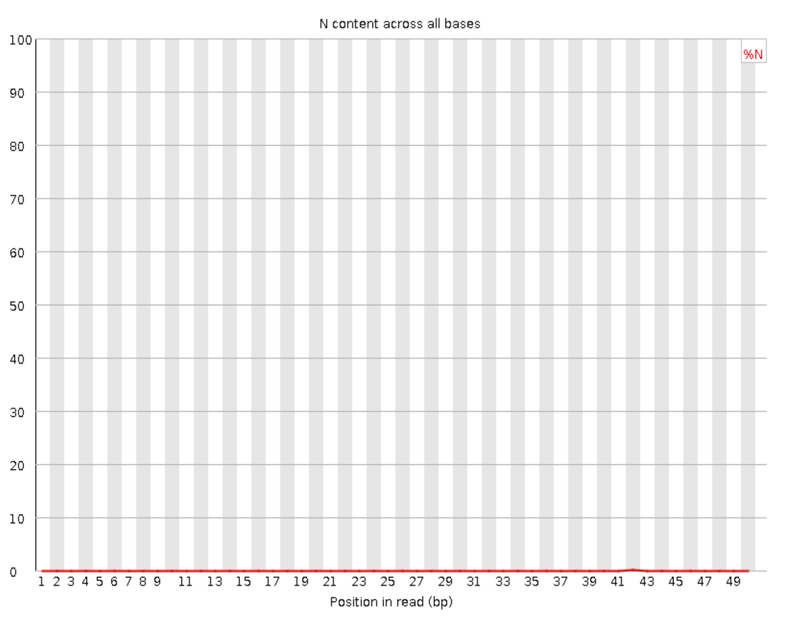

Sequence Length Distribution

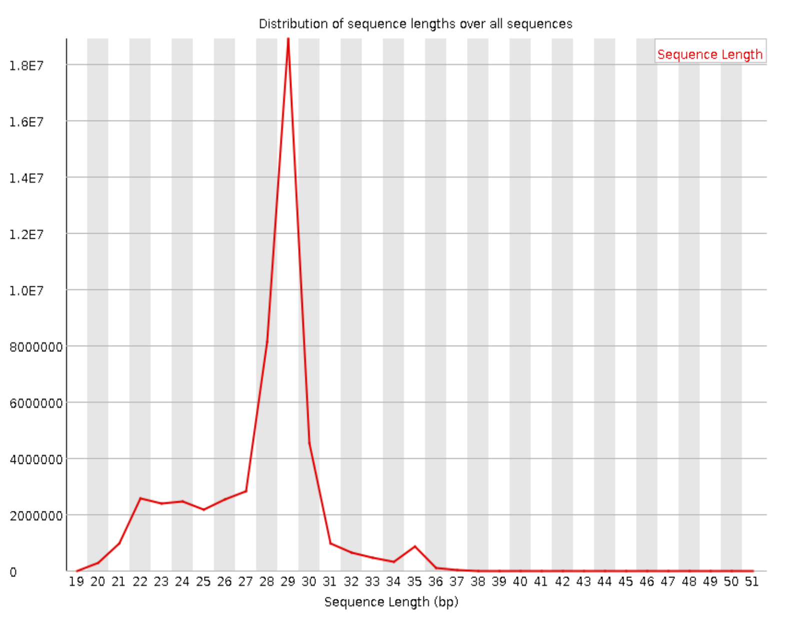

Sequence Duplication Levels

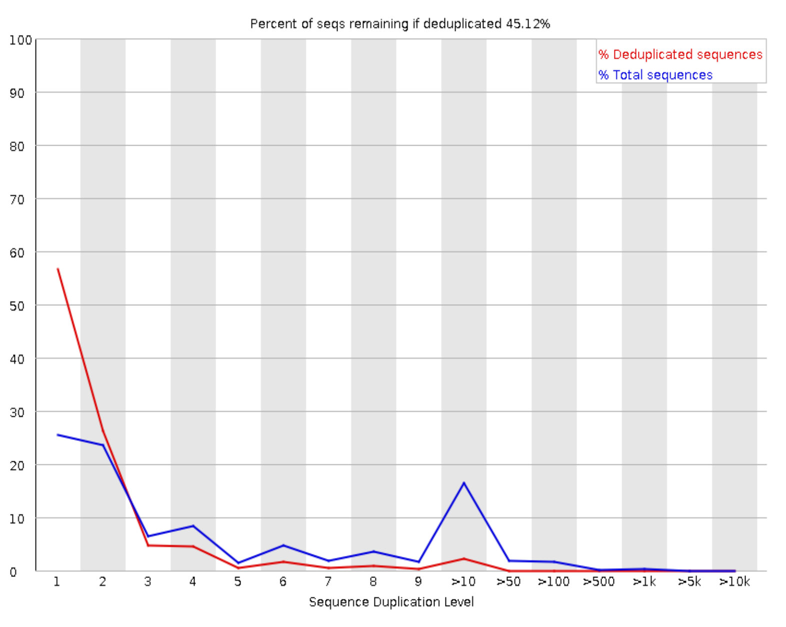

Overrepresented sequences

No overrepresented sequences

Adapter Content

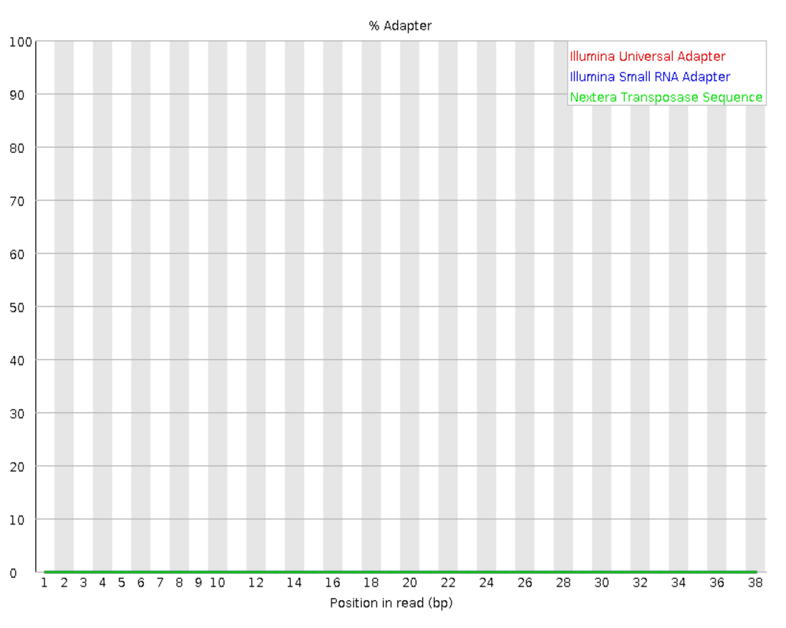

Kmer Content

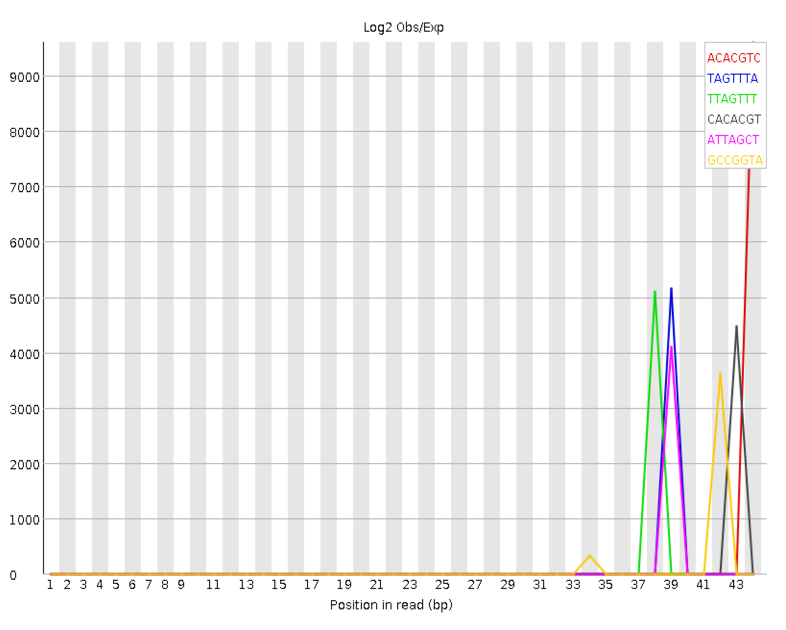

Sequence	Count	PValue	Obs/Exp Max	Max Obs/Exp Position
ACACGTC	615	8.424978E-5	9597.465	44
TAGTTTA	805	2.962759E-4	5159.714	39
TTAGTTT	595	3.07117E-4	5094.0894	38
CACACGT	1255	0.0	4467.9834	43
ATTAGCT	1010	4.6637375E-4	4112.4453	39
GCCGGTA	1470	5.910596E-4	3632.8596	42
GGTAATT	1620	0.0	3296.484	42
CGGTAGA	1825	7.418074E-4	3234.214	44
CCGGTAG	1785	7.884906E-4	3141.3552	43
TAATTCT	2030	0.0	2907.6064	44
GTAATTC	1990	0.0	2817.7483	43
TTTAATT	1930	0.0	2766.997	42
TTTATTT	1505	0.0010354578	2759.847	39
TTTAGTT	870	0.0011592836	2630.6917	37
CCGTGGG	3090	0.0	2629.9512	36
GGCCGGT	2055	0.0011550254	2598.688	41
TAGCTGC	2265	0.0014031174	2357.75	41
TCGGCGC	2385	0.0015557094	2239.121	42
GGGGATT	2800	0.0	2164.988	38
GCACACG	2470	0.0	2162.0664	42

Produced by FastQC (version 0.11.2)
