# Supplementary material for: PROTEOFORMER 2.0: Further Developments in the Ribosome Profiling-assisted Proteogenomic Hunt for New Proteoforms
Source: Mol Cell Proteomics. 2019 Apr 30;18(8 Suppl 1):S126–40. doi: 10.1074/mcp.RA118.001218 (PMC6692777; doi:10.1074/mcp.RA118.001218)
Supplement: Supplemental file S6 [file 142014_2_supp_322598_pqrw9k.pdf]

FastQC Report

Fri 27 Apr 2018
treat.sam

Summary

- Basic Statistics
- Per base sequence quality
- Per sequence quality scores
- Per base sequence content
- Per sequence GC content
- Per base N content
- Sequence Length Distribution
- Sequence Duplication Levels
- Overrepresented sequences
- Adapter Content
- Kmer Content

Basic Statistics

Measure	Value
Filename	treat.sam
File type	Conventional base calls
Encoding	Sanger / Illumina 1.9
Total Sequences	68465701
Sequences flagged as poor quality	0
Sequence length	20-50
%GC	51

Per base sequence quality

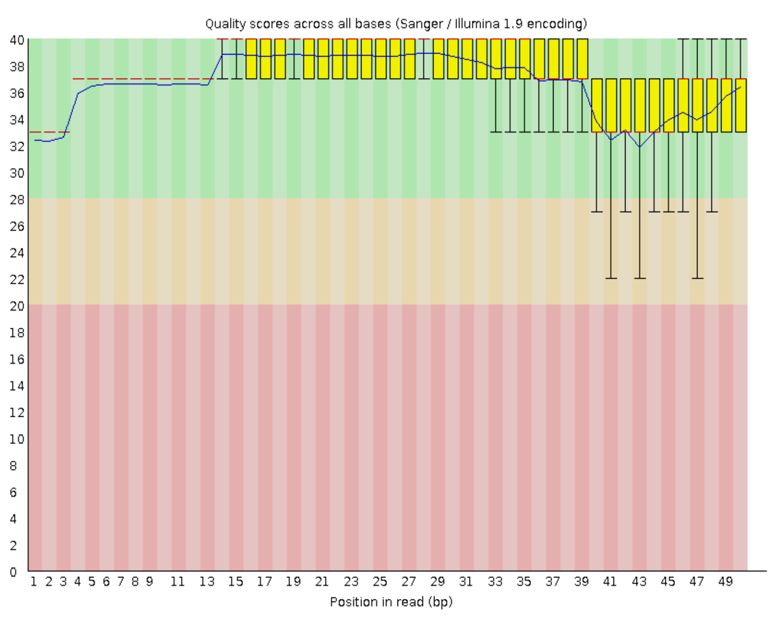

Per sequence quality scores

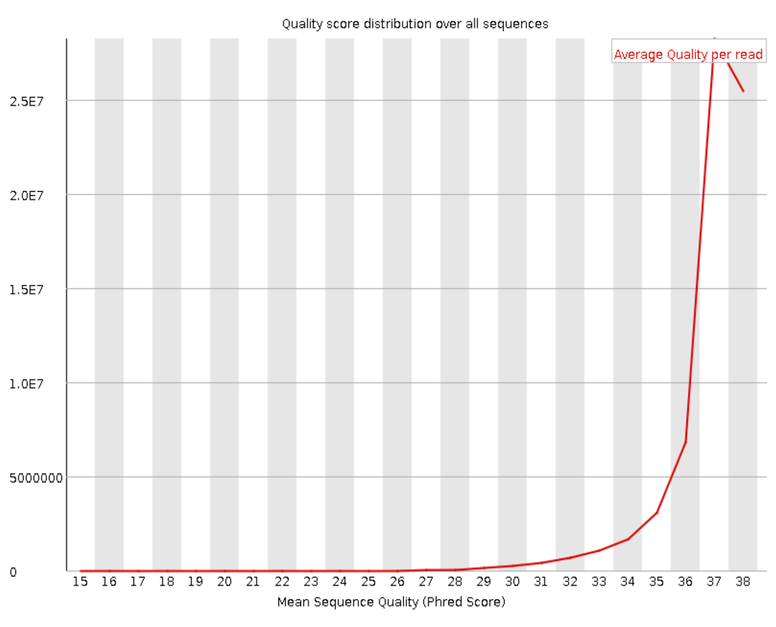

Per base sequence content

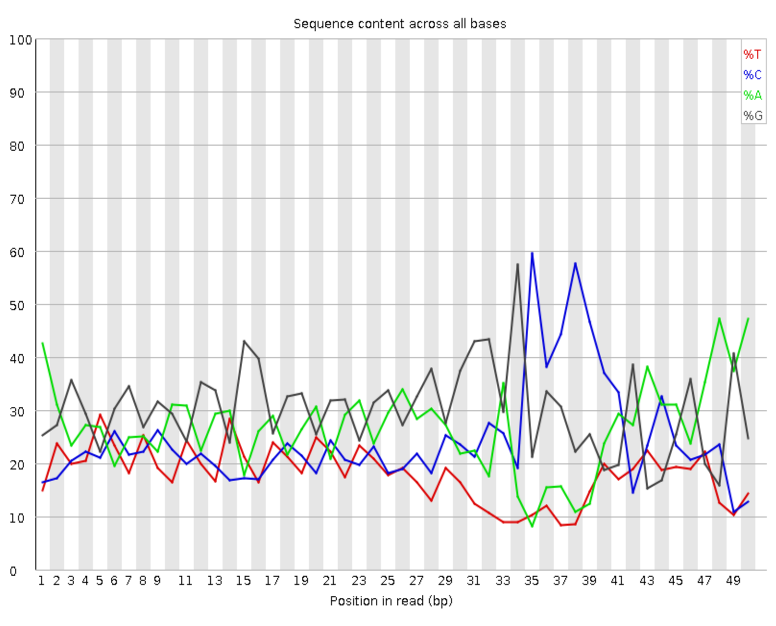

Per sequence GC content

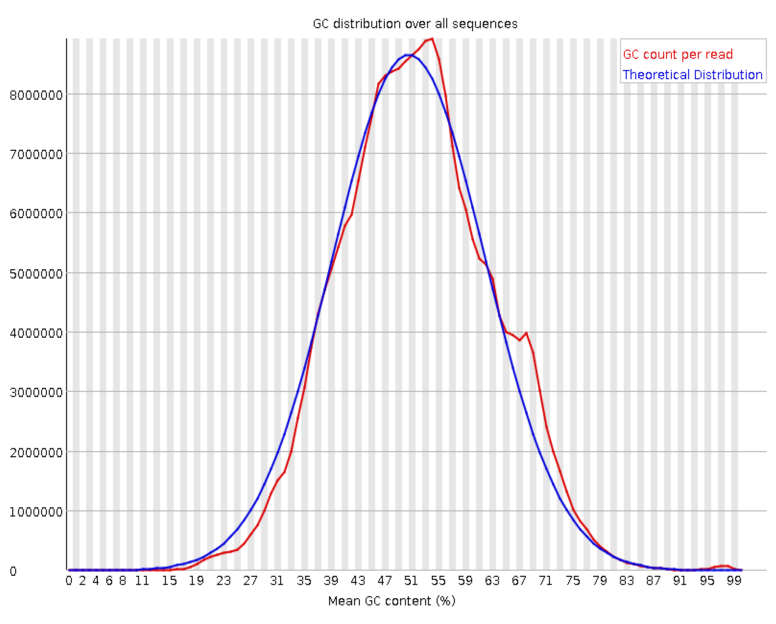

Per base N content

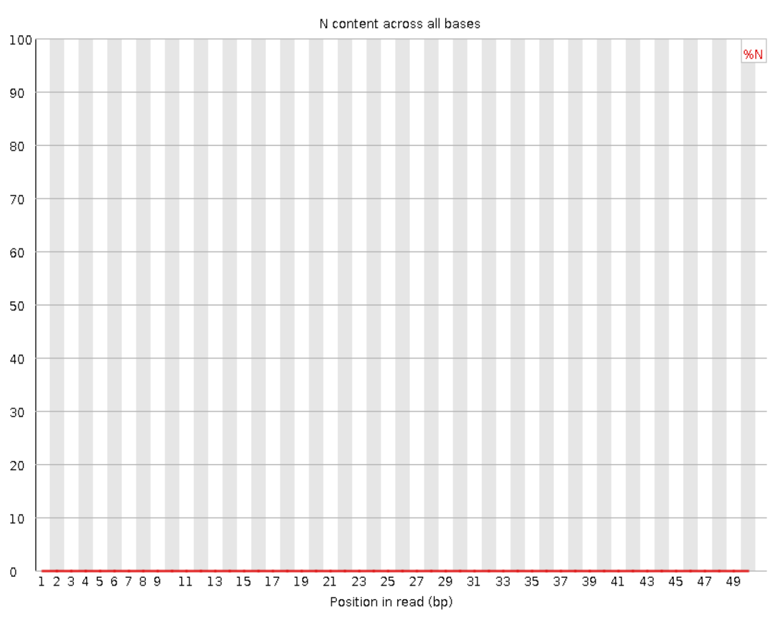

Sequence Length Distribution

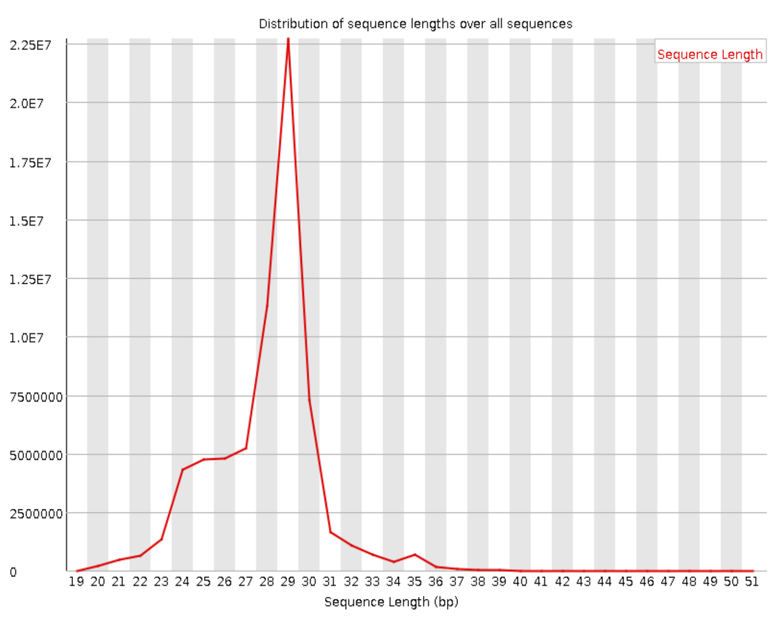

Sequence Duplication Levels

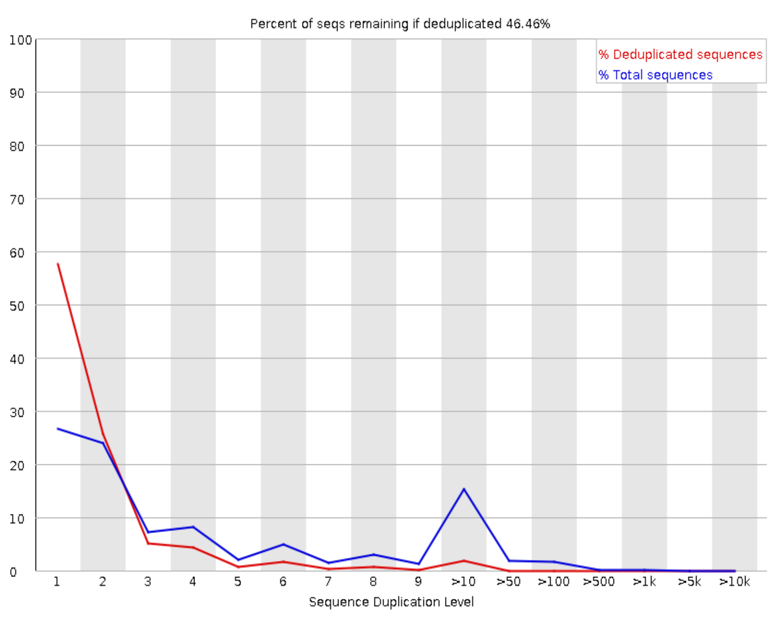

Overrepresented sequences

No overrepresented sequences

Adapter Content

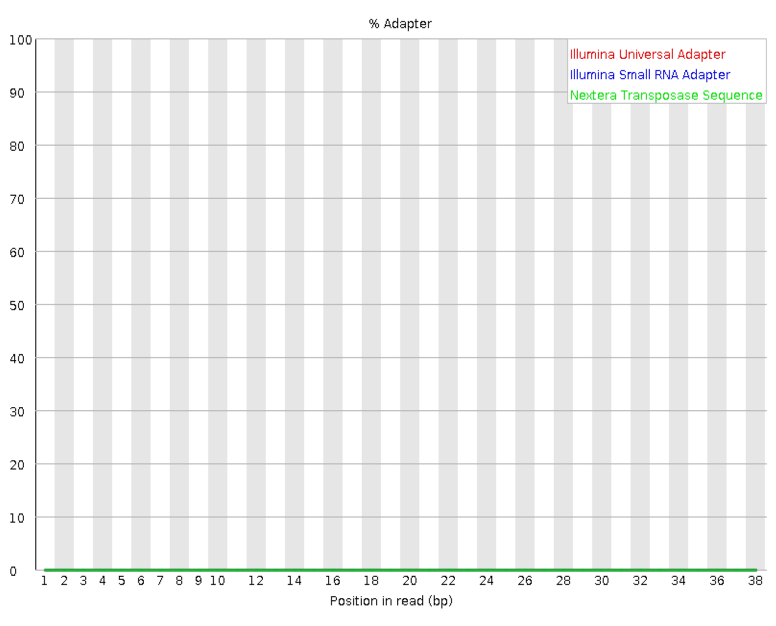

Kmer Content

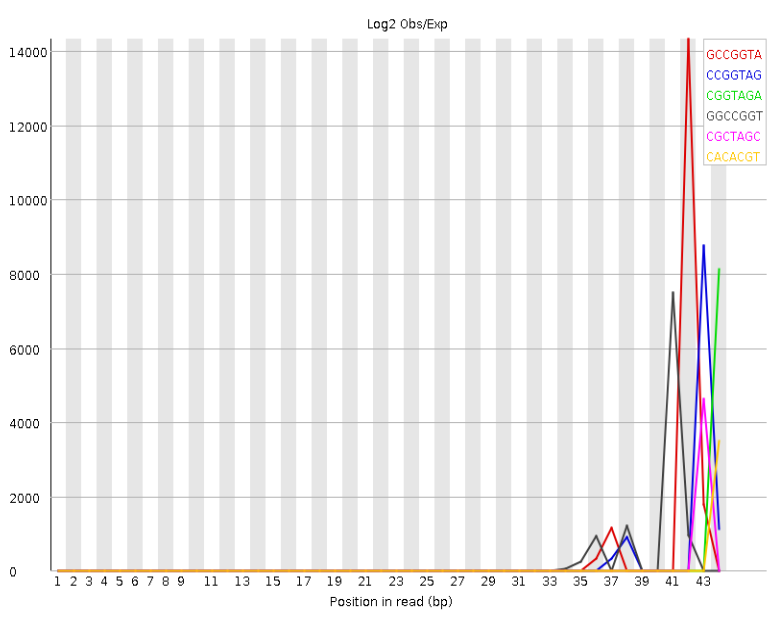

Sequence	Count	PValue	Obs/Exp Max	Max Obs/Exp Position
GCCGGTA	1530	0.0	14317.783	42
CCGGTAG	2500	0.0	8762.485	43
CGGTAGA	2790	0.0	8147.978	44
GGCCGGT	2765	0.0	7512.886	41
CGCTAGC	590	2.1464075E-10	4641.1465	43
CACACGT	1615	0.0	3519.0188	44
AGGCCGG	6175	0.0	2748.1133	40
ACTCGAT	1045	0.0010867242	2719.2417	44
CGAAAAA	7275	0.0	2587.7183	39
TAACAAT	2295	1.5200967E-6	2386.2976	42
AAATAAC	3760	9.094947E-12	2071.7883	41
AACAATA	2780	2.7014867E-6	1969.983	43
CCGCTAG	1480	0.0	1850.1869	42
GCTAGCA	1545	0.0023751701	1839.2281	44
CACGATC	1480	0.0026144295	1754.4874	41
ATAACAA	3100	4.405103E-6	1675.2526	41
TAGCCGG	1775	0.0	1636.1873	33
GCACACG	3365	0.0	1627.5045	43
ACAATAG	1750	0.0030471492	1623.7758	44
ATTCAAT	1790	0.0031880108	1587.4902	44

Produced by FastQC (version 0.11.2)
