# Supplementary material for: PROTEOFORMER 2.0: Further Developments in the Ribosome Profiling-assisted Proteogenomic Hunt for New Proteoforms
Source: Mol Cell Proteomics. 2019 Apr 30;18(8 Suppl 1):S126–40. doi: 10.1074/mcp.RA118.001218 (PMC6692777; doi:10.1074/mcp.RA118.001218)
Supplement: Supplemental file S7 [file 142014_2_supp_322599_pqrw9k.pdf]

FastQC Report

Mon 13 Aug 2018
untreat.sam

Summary

- Basic Statistics
- Per base sequence quality
- Per sequence quality scores
- Per base sequence content
- Per sequence GC content
- Per base N content
- Sequence Length Distribution
- Sequence Duplication Levels
- Overrepresented sequences
- Adapter Content
- Kmer Content

Basic Statistics

Measure	Value
Filename	untreat.sam
File type	Conventional base calls
Encoding	Sanger / Illumina 1.9
Total Sequences	124445850
Sequences flagged as poor quality	0
Sequence length	20-36
%GC	51

Per base sequence quality

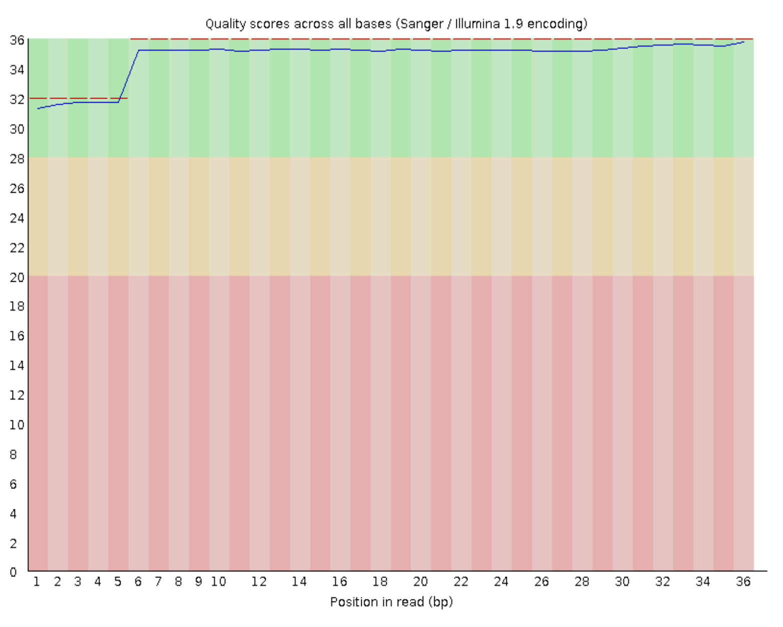

Per sequence quality scores

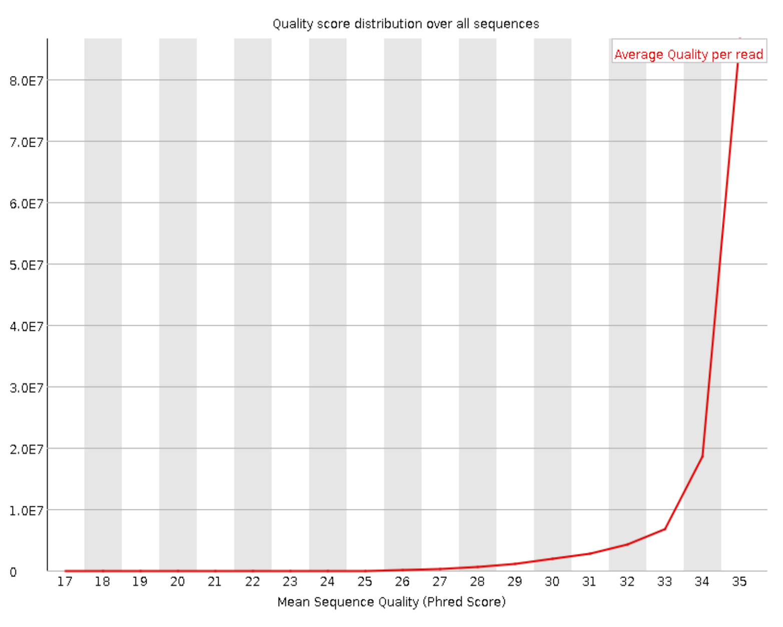

Per base sequence content

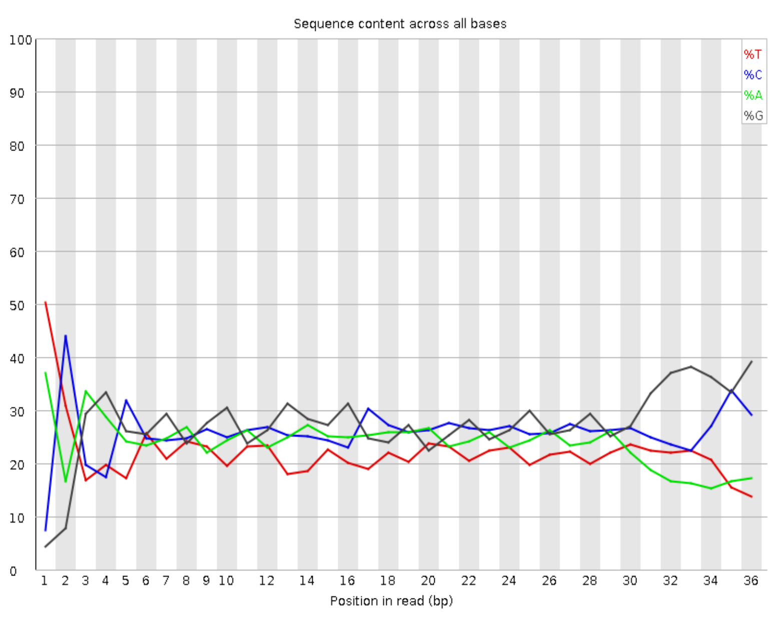

Per sequence GC content

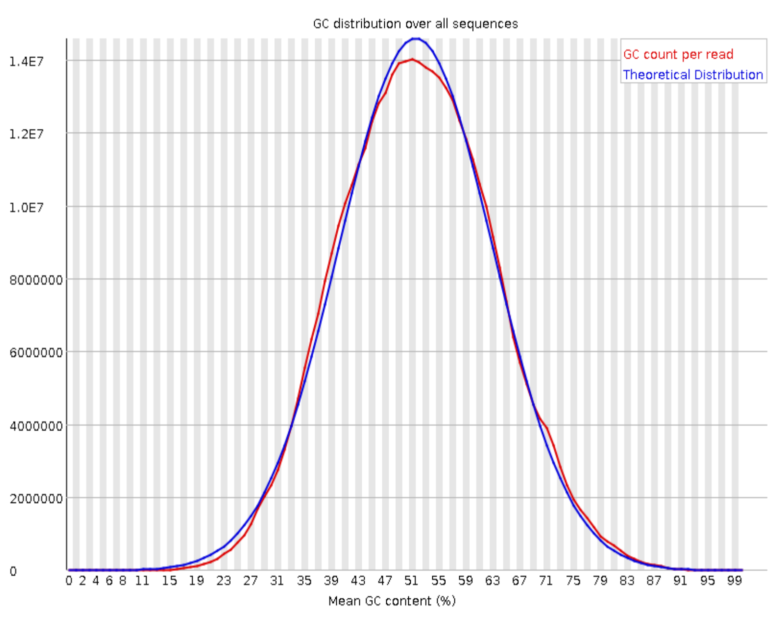

Per base N content

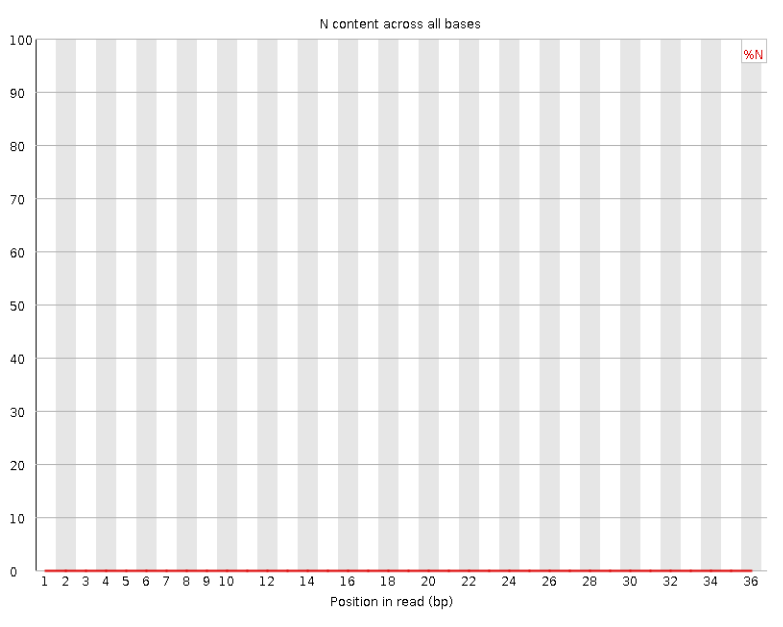

Sequence Length Distribution

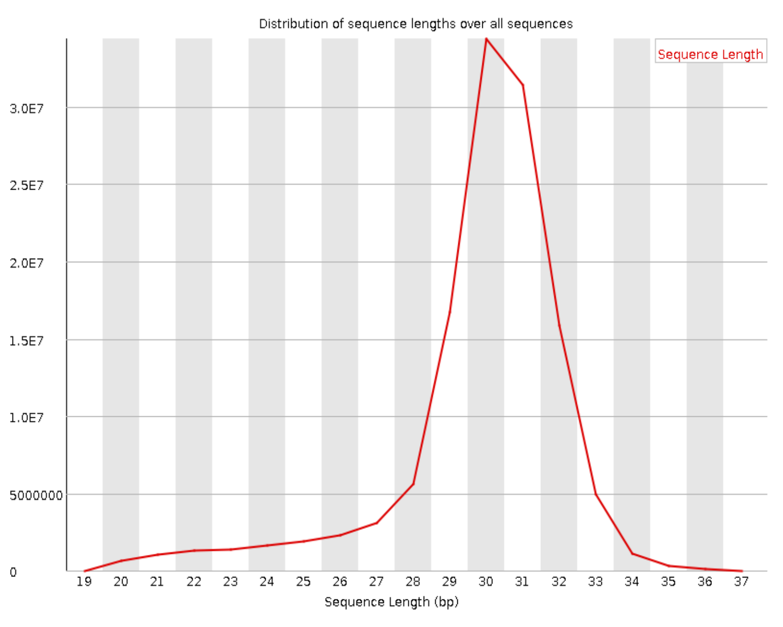

Sequence Duplication Levels

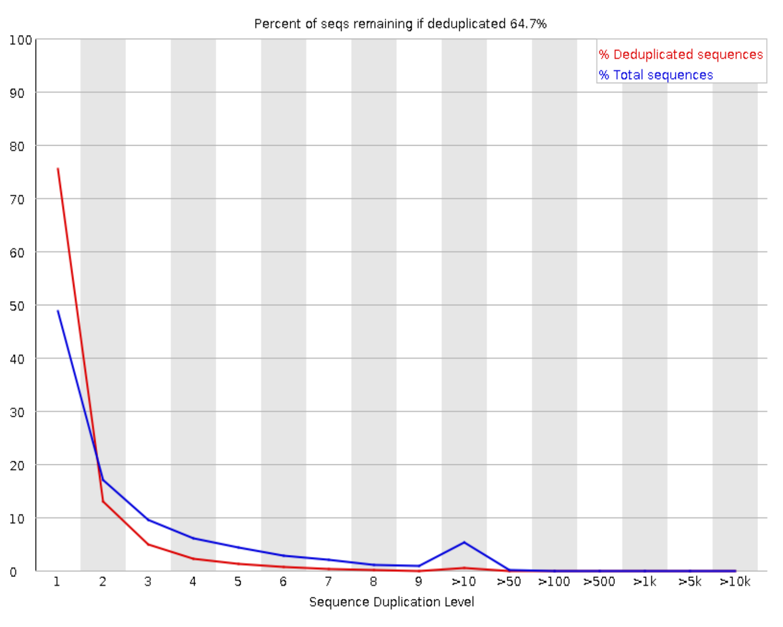

Overrepresented sequences

No overrepresented sequences

Adapter Content

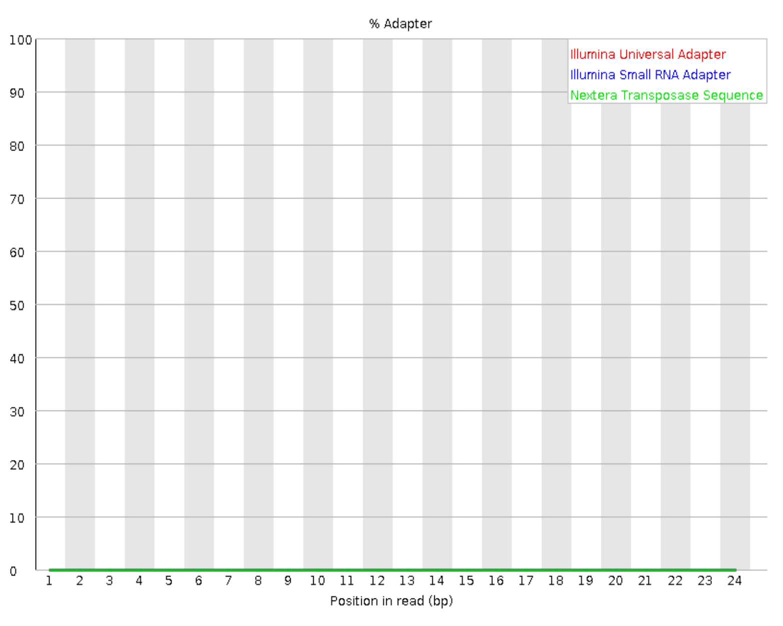

Kmer Content

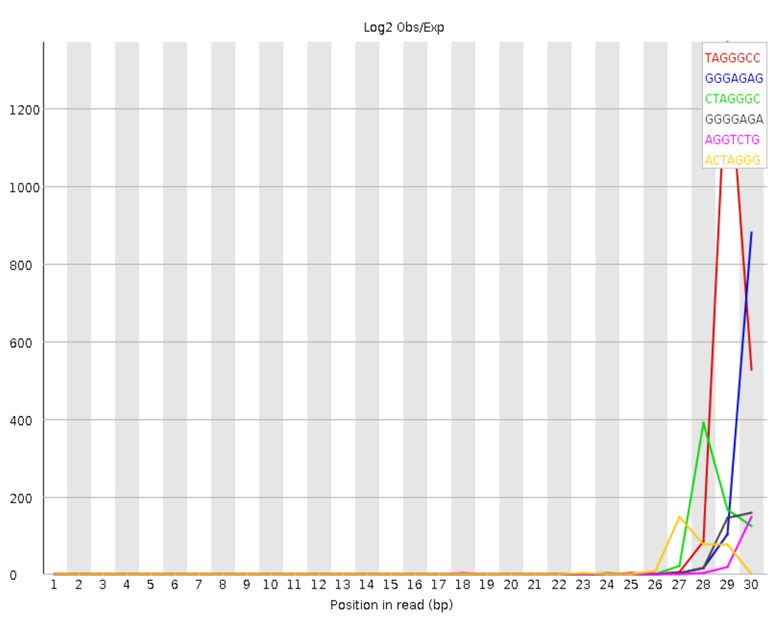

Sequence	Count	PValue	Obs/Exp Max	Max Obs/Exp Position
TAGGGCC	10845	0.0	1370.7036	29
GGGAGAG	29905	0.0	880.93665	30
CTAGGGC	11605	0.0	392.6474	28
GGGGAGA	23780	0.0	159.4307	30
AGGTCTG	25375	0.0	149.40933	30
ACTAGGG	7750	0.0	148.97504	27
CATTTCG	6225	1.07320375E-10	109.31452	30
AGGGCCA	30055	0.0	106.73735	30
TTAGACG	2625	0.0	103.38996	27
TCCCCCC	6890	0.0	98.76385	30
TTCTAGG	9890	0.0	98.29306	30
AAGGTCT	24445	0.0	95.95199	29
TTTCTTT	22435	0.0	95.326965	30
ACTGAGG	25845	0.0	90.27218	30
CGGGAGA	24885	0.0	86.91085	29
AGGGAGA	38715	0.0	66.879486	29
GGGTGTA	4430	0.002833302	65.83195	30
AGGGGGG	4430	0.002833302	65.83195	30
TTTAGAC	13470	0.0	64.83344	26
GCGGGGG	10860	8.8475645E-9	62.65957	30

Produced by FastQC (version 0.11.2)
