# Supplementary material for: PROTEOFORMER 2.0: Further Developments in the Ribosome Profiling-assisted Proteogenomic Hunt for New Proteoforms
Source: Mol Cell Proteomics. 2019 Apr 30;18(8 Suppl 1):S126–40. doi: 10.1074/mcp.RA118.001218 (PMC6692777; doi:10.1074/mcp.RA118.001218)
Supplement: Supplemental file S8 [file 142014_2_supp_322600_pqrw9k.pdf]

FastQC Report

Mon 13 Aug 2018
treat.sam

Summary

- Basic Statistics
- Per base sequence quality
- Per sequence quality scores
- Per base sequence content
- Per sequence GC content
- Per base N content
- Sequence Length Distribution
- Sequence Duplication Levels
- Overrepresented sequences
- Adapter Content
- Kmer Content

Basic Statistics

Measure	Value
Filename	treat.sam
File type	Conventional base calls
Encoding	Sanger / Illumina 1.9
Total Sequences	97308141
Sequences flagged as poor quality	0
Sequence length	20-36
%GC	51

Per base sequence quality

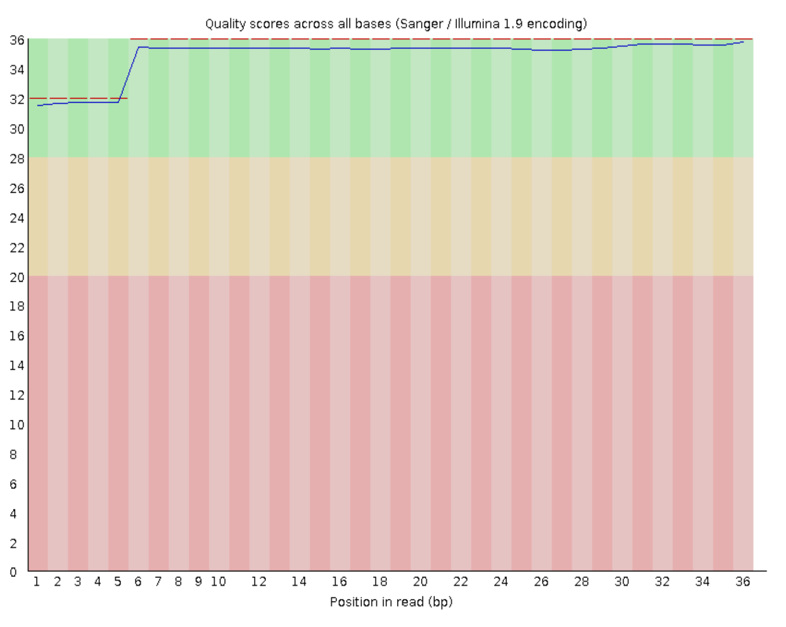

Per sequence quality scores

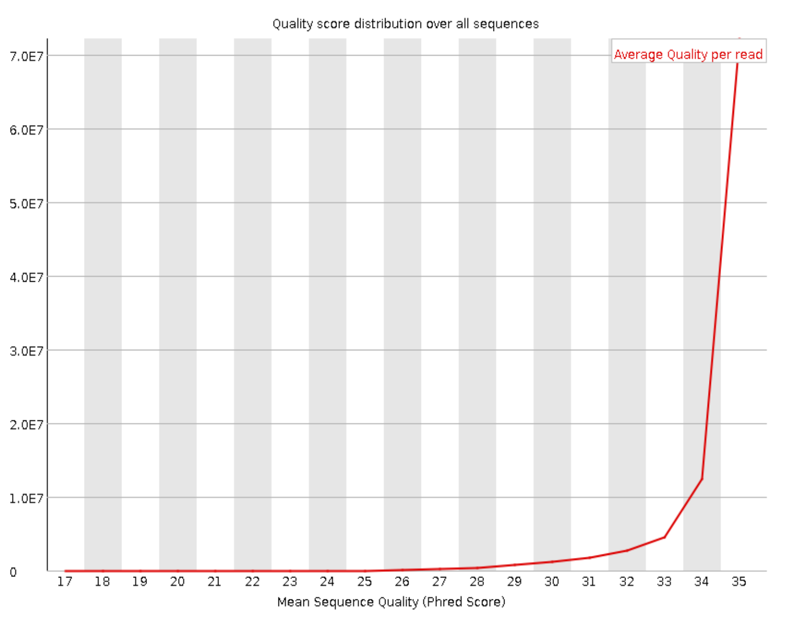

Per base sequence content

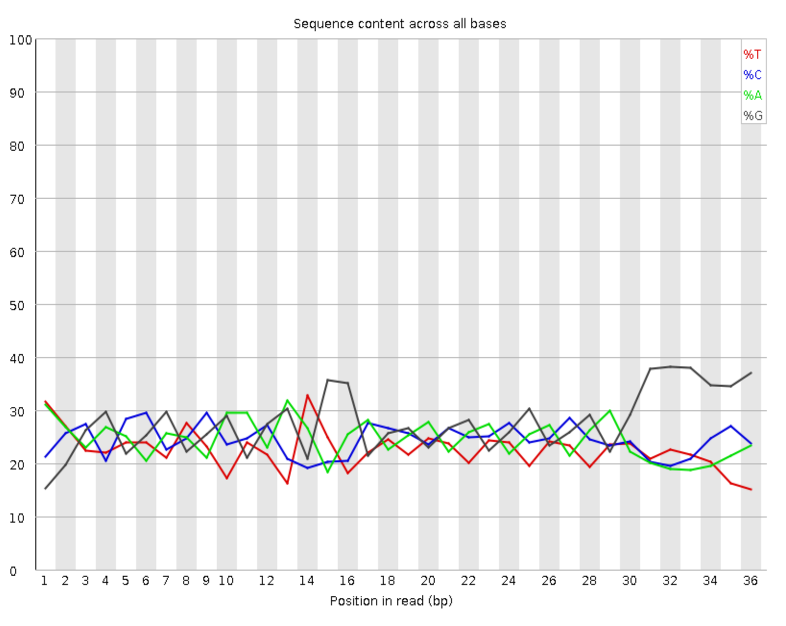

Per sequence GC content

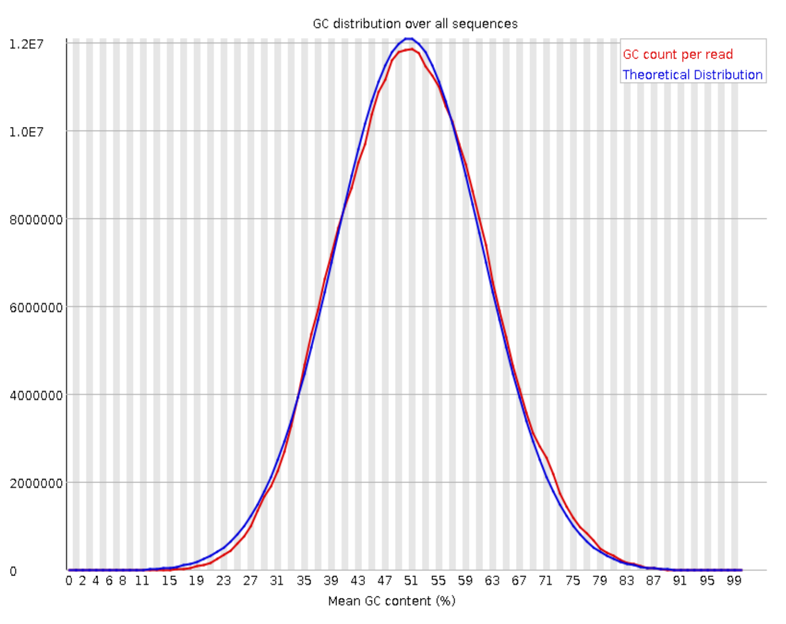

Per base N content

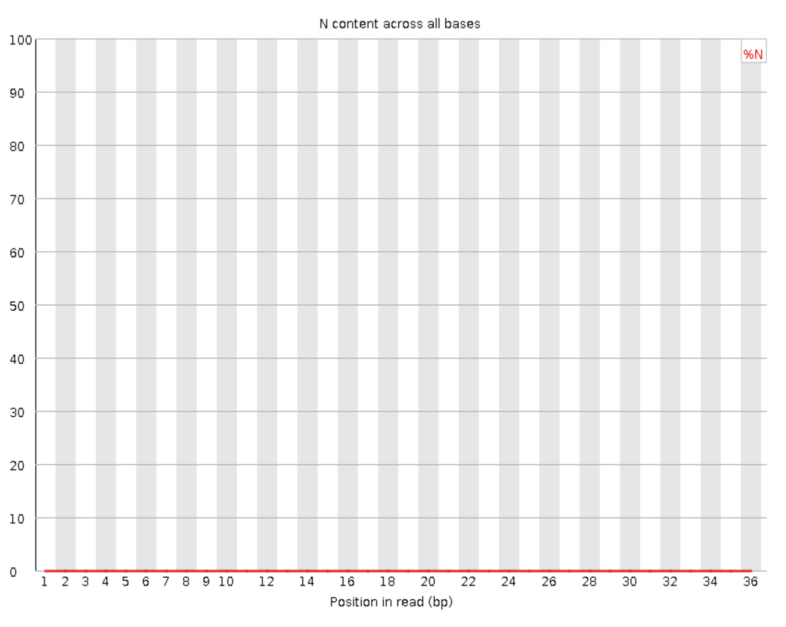

Sequence Length Distribution

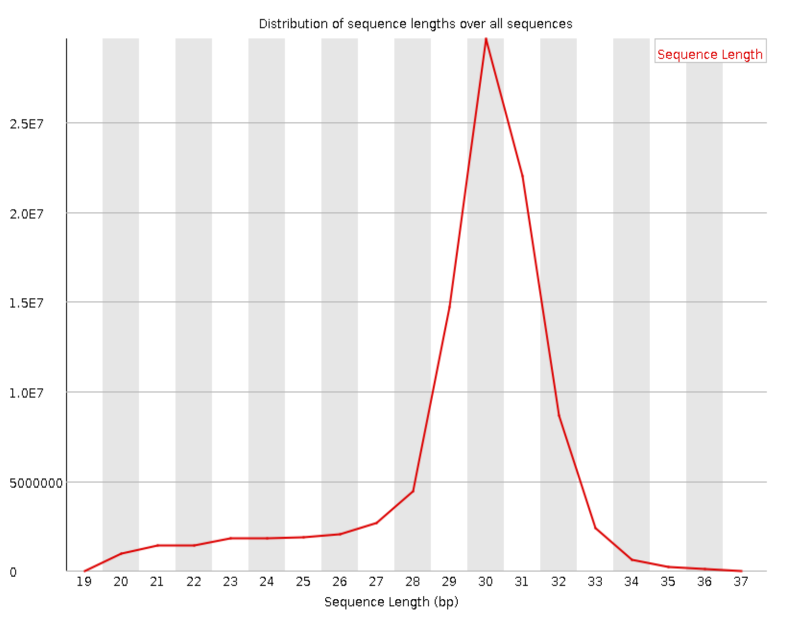

Sequence Duplication Levels

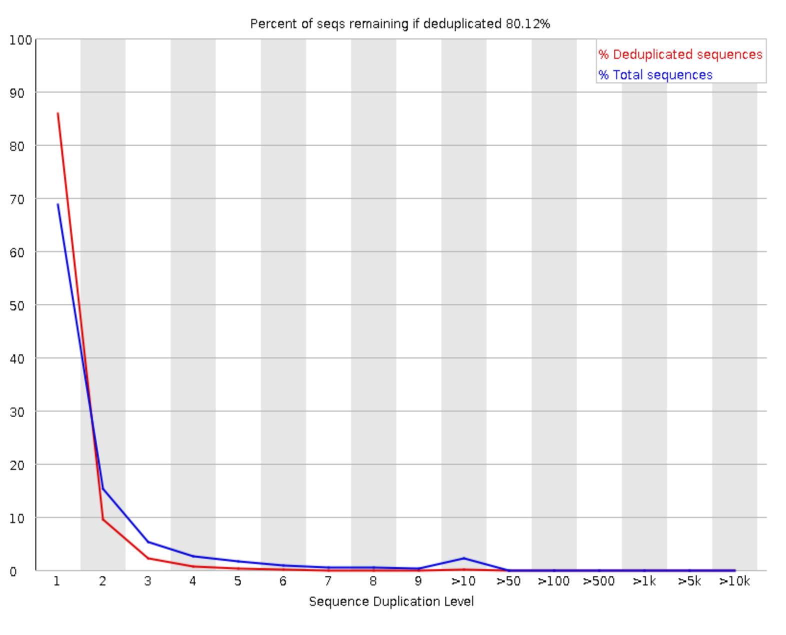

Overrepresented sequences

No overrepresented sequences

Adapter Content

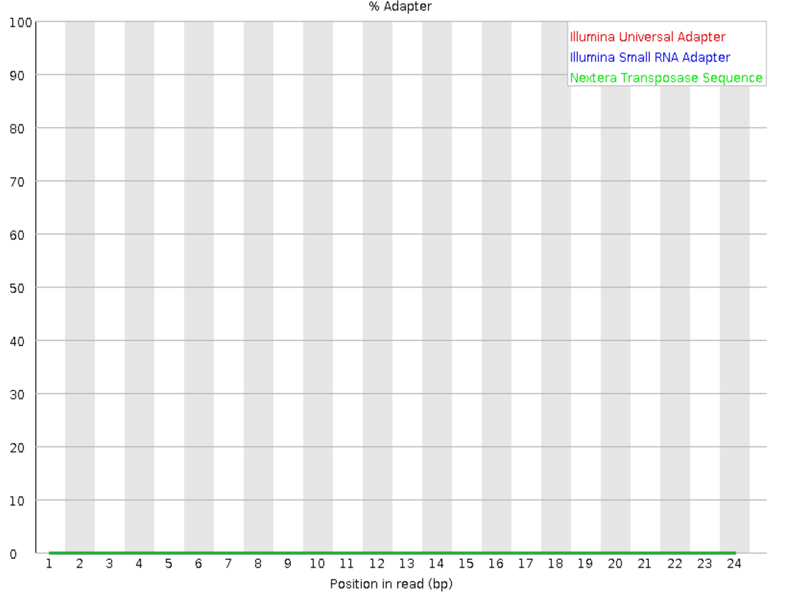

Kmer Content

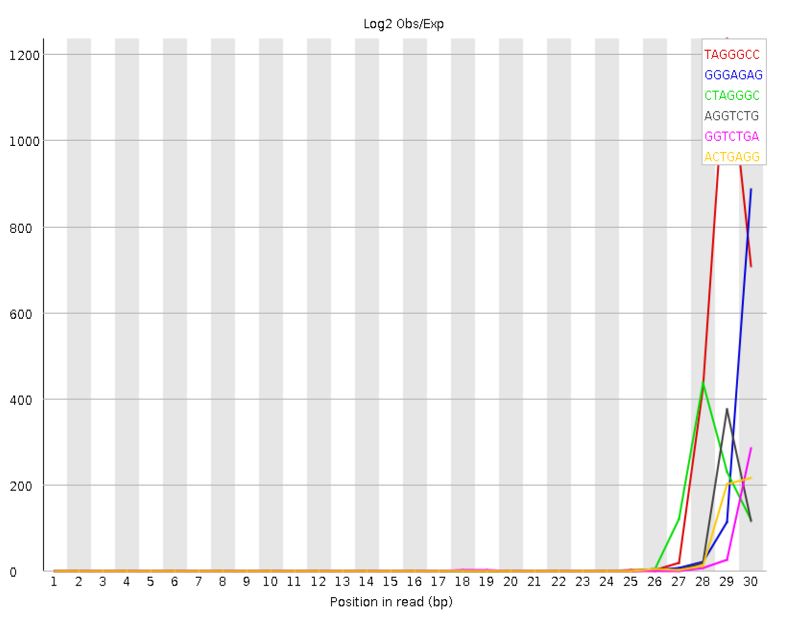

Sequence	Count	PValue	Obs/Exp Max	Max Obs/Exp Position
TAGGGCC	7755	0.0	1234.9674	29
GGGAGAG	24000	0.0	887.1212	30
CTAGGGC	8195	0.0	439.2806	28
AGGTCTG	23515	0.0	376.26135	29
GGTCTGA	18195	0.0	285.7974	30
ACTGAGG	21790	0.0	216.13171	30
AAGGTCT	22830	0.0	179.20808	28
ACTAGGG	6800	0.0	157.79787	27
TTAGACG	3240	0.0	156.3909	27
GGGGAGA	15020	0.0	152.01727	29
TCCCCCC	2815	2.5806494E-6	139.4171	30
AGGGAGA	23730	0.0	129.62965	29
AGTTACG	5290	4.0708983E-9	111.28331	30
CGGGAGA	12815	0.0	98.985535	29
CACTGAG	24300	0.0	95.470726	28
GGTAAGC	16620	0.0	94.45467	30
TTTAGAC	12325	0.0	85.34909	26
AGGGCCA	20510	0.0	76.54006	30
TCCCCGC	7890	6.531445E-8	74.612	30
CCTAGTT	3915	0.0	67.13926	28

Produced by FastQC (version 0.11.2)
