# Supplementary material for: PROTEOFORMER 2.0: Further Developments in the Ribosome Profiling-assisted Proteogenomic Hunt for New Proteoforms
Source: Mol Cell Proteomics. 2019 Apr 30;18(8 Suppl 1):S126–40. doi: 10.1074/mcp.RA118.001218 (PMC6692777; doi:10.1074/mcp.RA118.001218)

Analysis information

Feature	Value
Species	human
Input sam file	../STAR/fastq2/treat.sam
Ensembl version	92
Ensembl database	../ENS_hsa_92.db
Sample treatment	treated
Mapping unique?	N
Mapping first rank?	N
MappingQC unique?	N
Selected offset source	plastid
Mapped genomic sequences	37 095 737
Analysis date	Friday 28 Sep 2018
Analysis time	01:11:24

Plastid offset analysis

RPF length	Offset
22	6
23	12
24	12
25	12
26	12
27	12
28	12
29	12
30	12
31	12
32	12
33	13
34	12

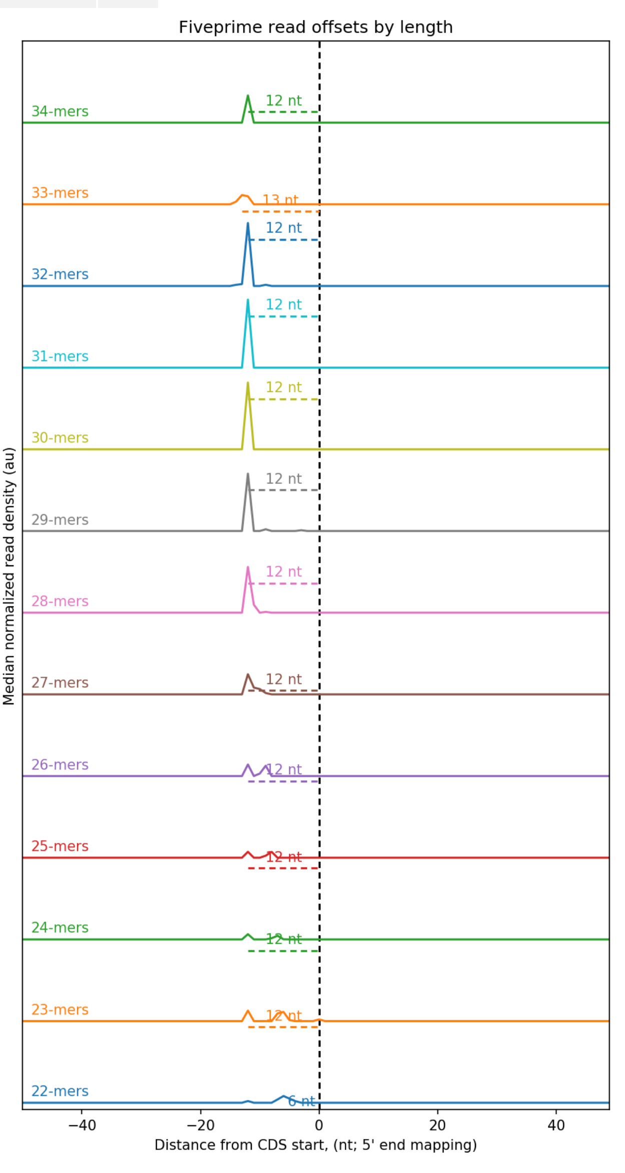

Gene distributions

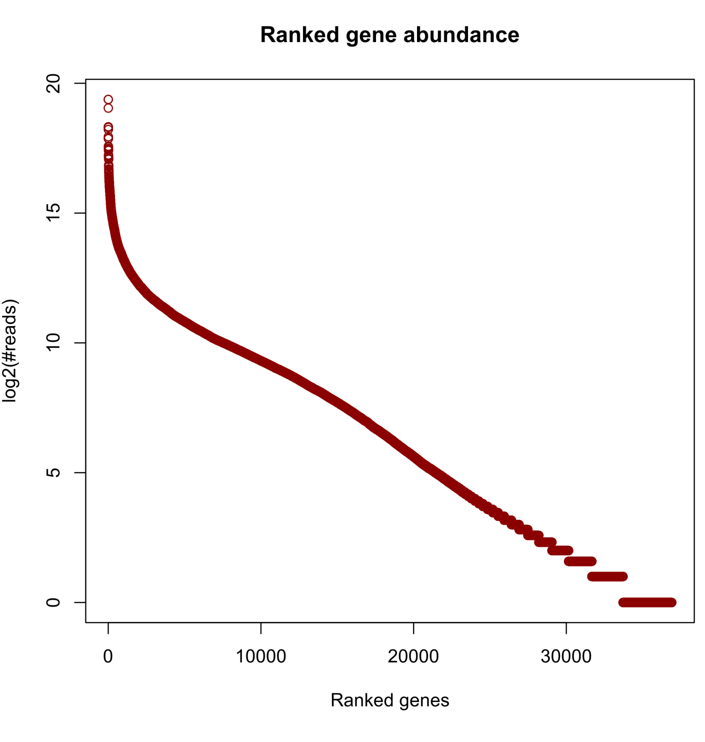

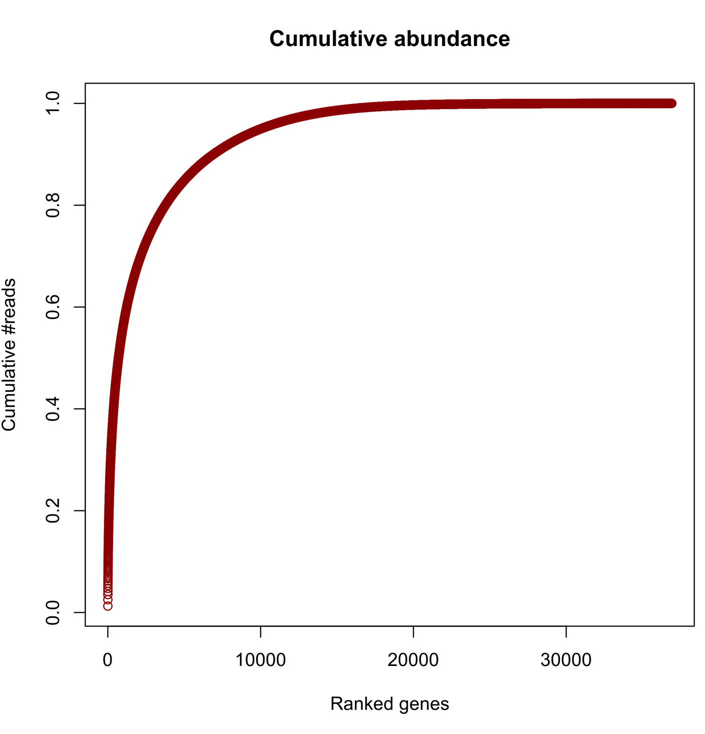

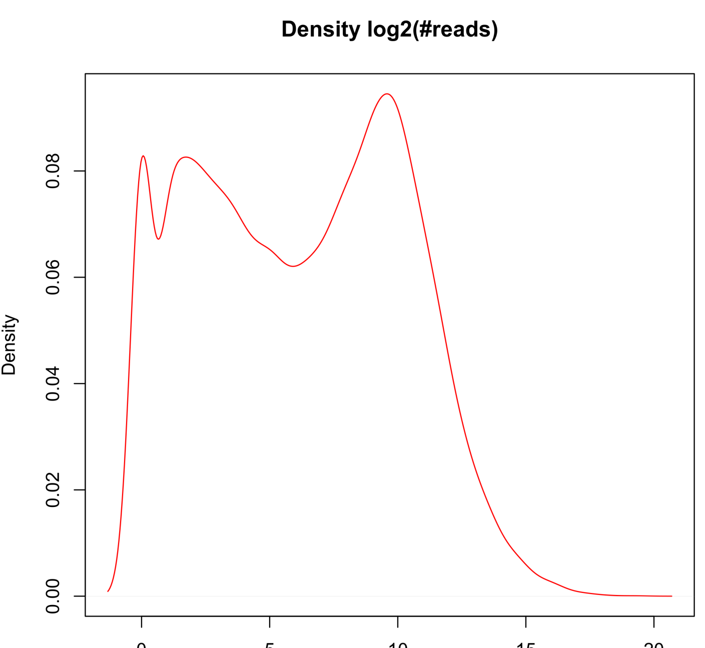

Metagenic classification

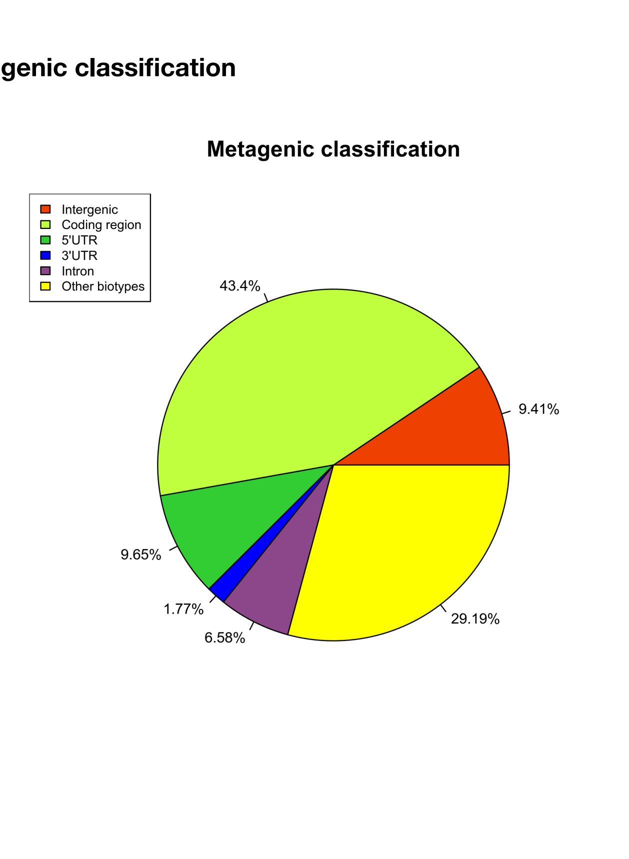

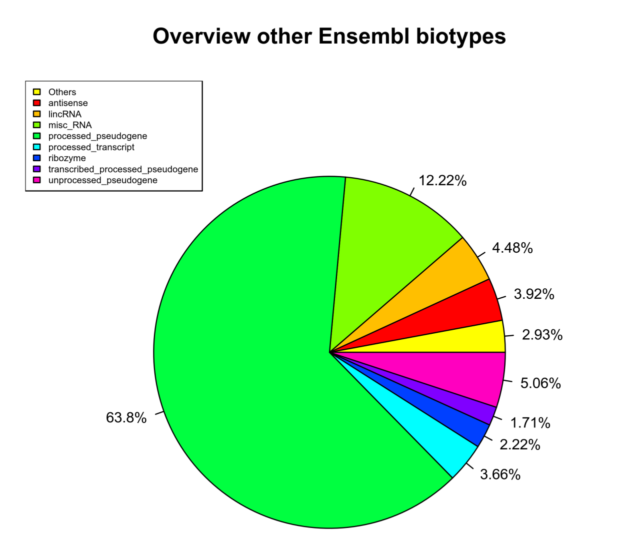

Total phase distribution

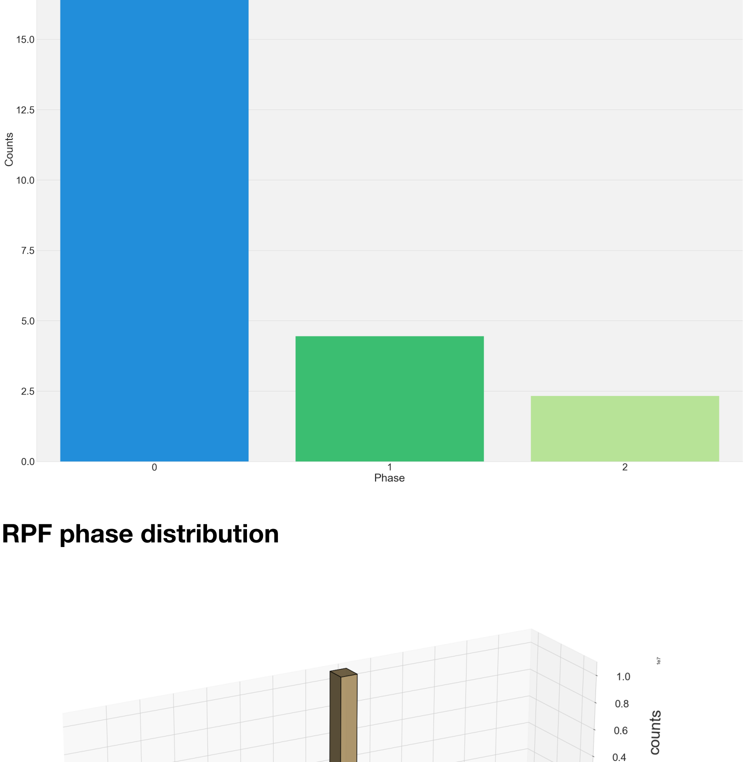

RPF phase distribution

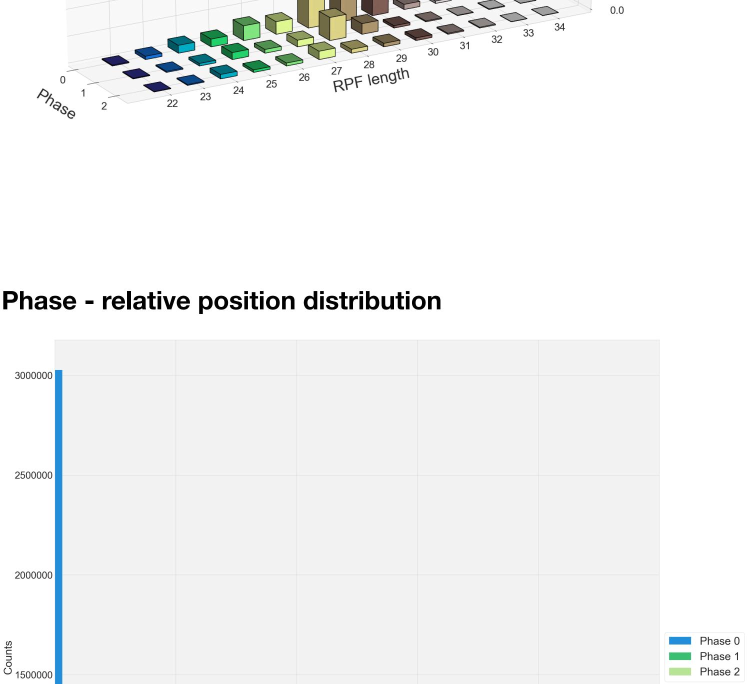

Phase - relative position distribution

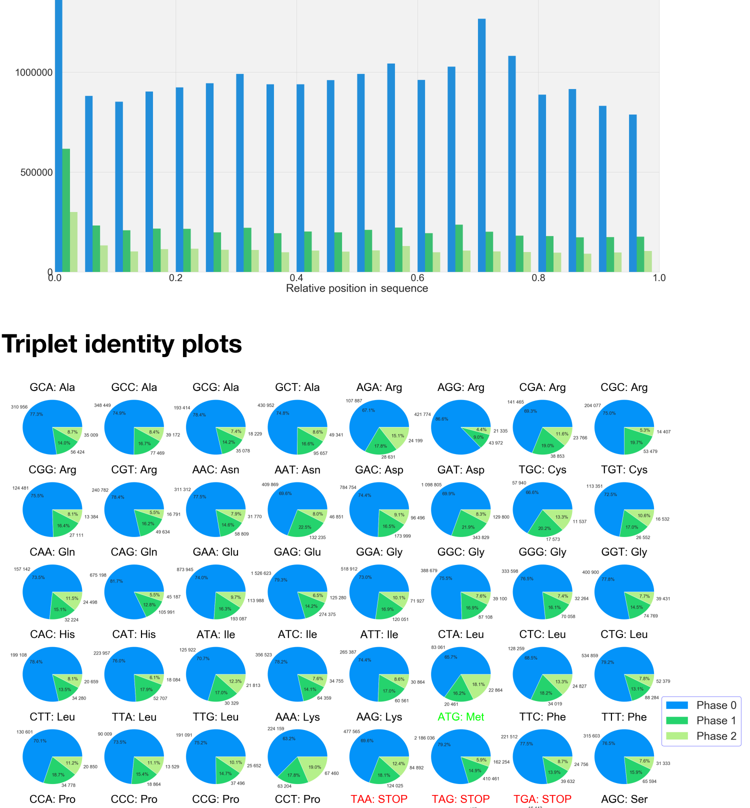

Triplet identity plots

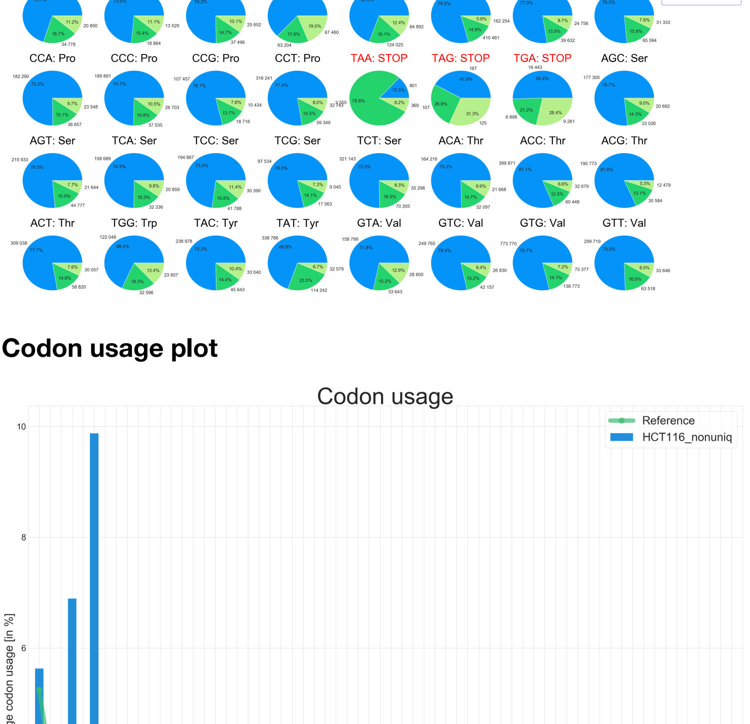

Codon usage plot

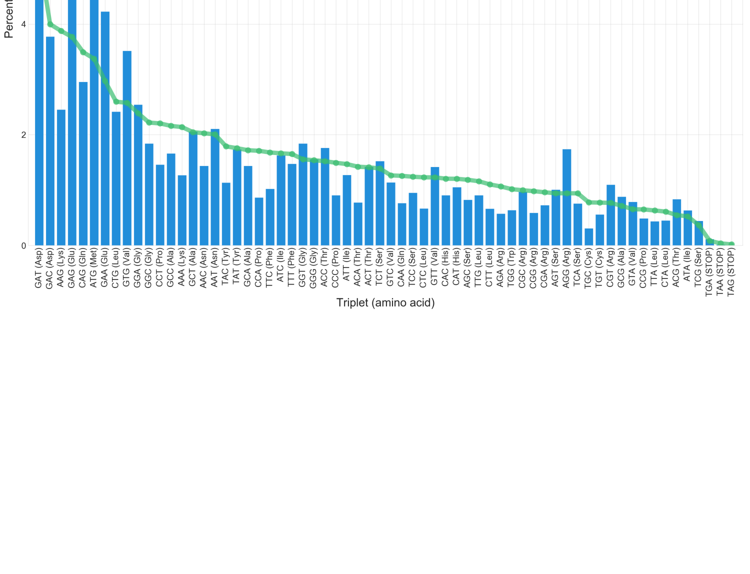

Supplement: Supplemental file S10 [file 142014_2_supp_322602_pqrw9l.pdf]
