# Supplementary material for: PROTEOFORMER 2.0: Further Developments in the Ribosome Profiling-assisted Proteogenomic Hunt for New Proteoforms
Source: Mol Cell Proteomics. 2019 Apr 30;18(8 Suppl 1):S126–40. doi: 10.1074/mcp.RA118.001218 (PMC6692777; doi:10.1074/mcp.RA118.001218)

Analysis information

Feature	Value
Species	human
Input sam file	../STAR/fastq1/untreat.sam
Ensembl version	92
Ensembl database	../ENS_hsa_92.db
Sample treatment	untreated
Mapping unique?	Y
Mapping first rank?	N
MappingQC unique?	Y
Selected offset source	plastid
Mapped genomic sequences	124 995 073
Analysis date	Thursday 27 Sep 2018
Analysis time	20:42:59

Plastid offset analysis

RPF length	Offset
22	3
23	5
24	3
25	3
26	12
27	12
28	12
29	12
30	12
31	12
32	12
33	13
34	13

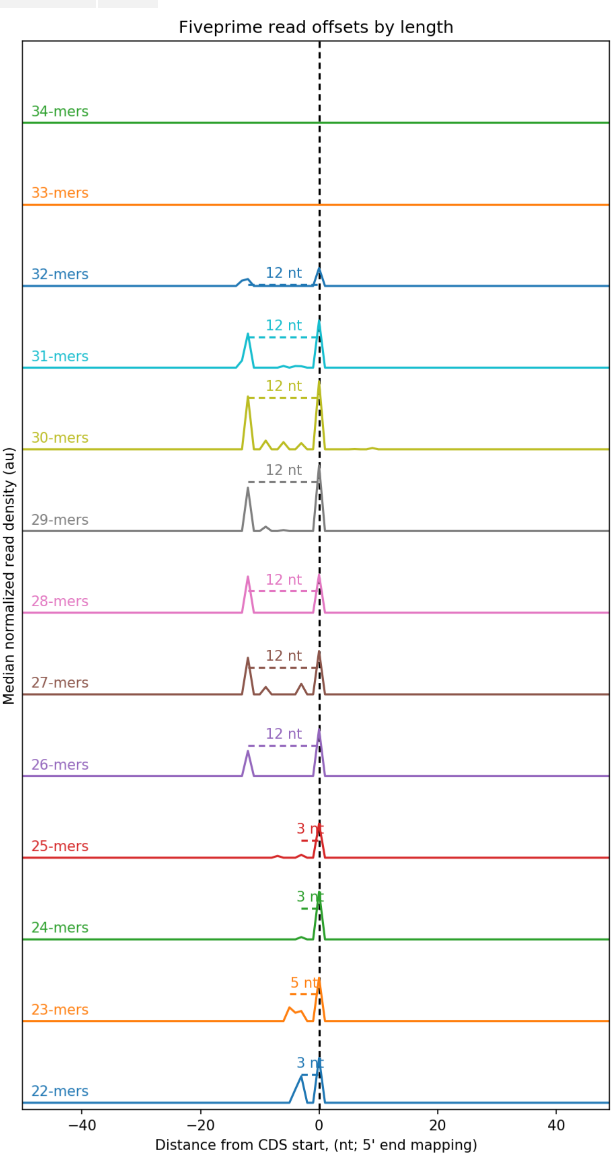

Gene distributions

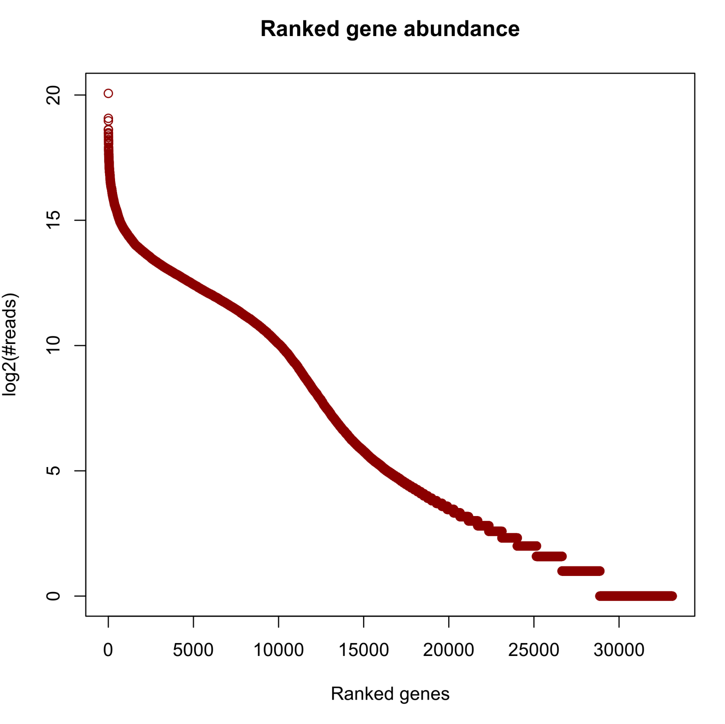

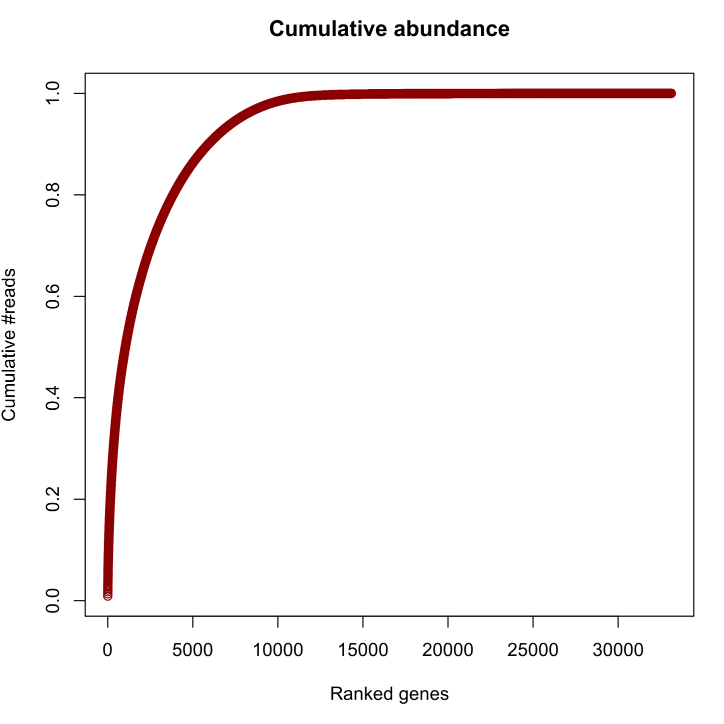

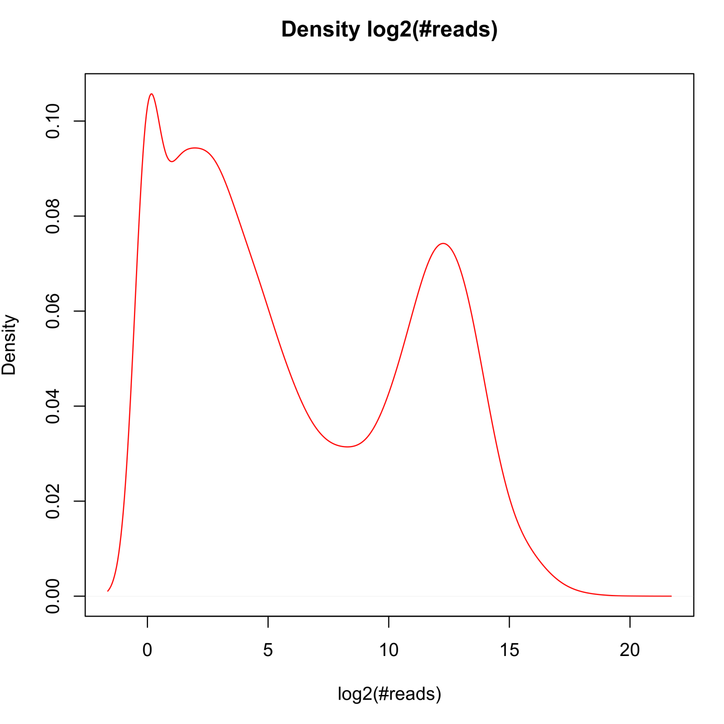

Metagenic classification

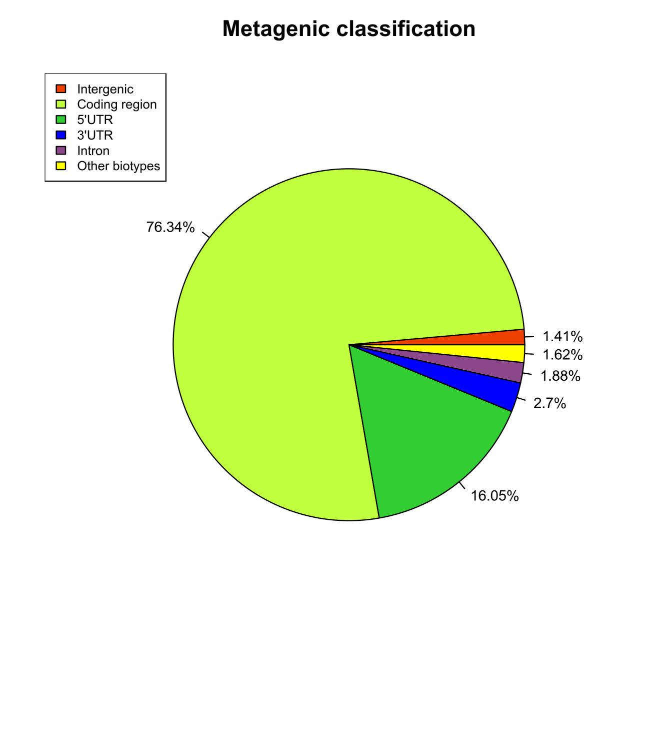

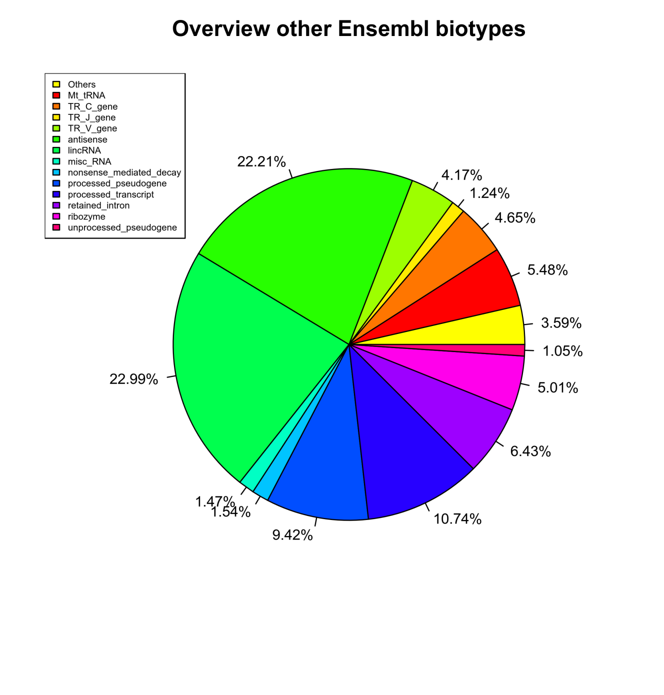

Total phase distribution

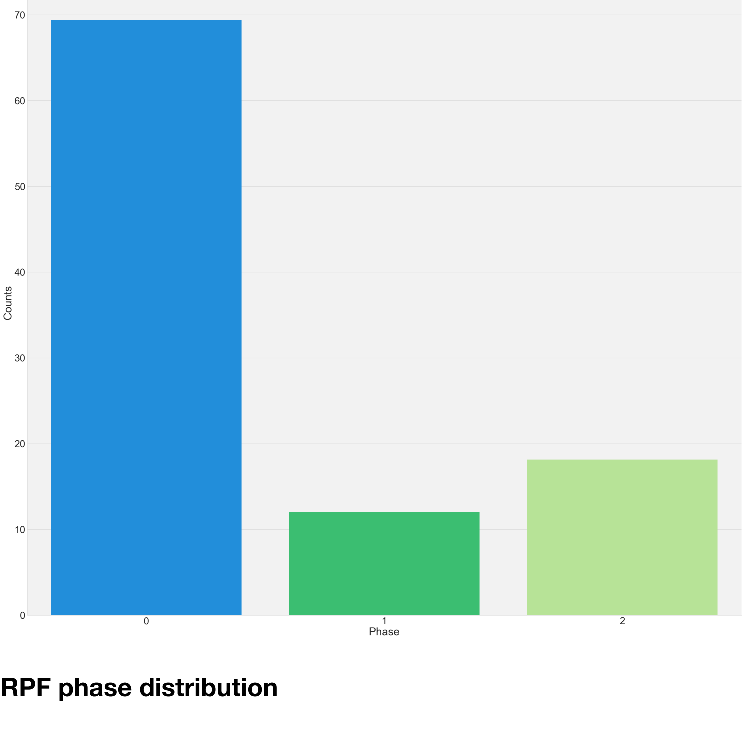

RPF phase distribution

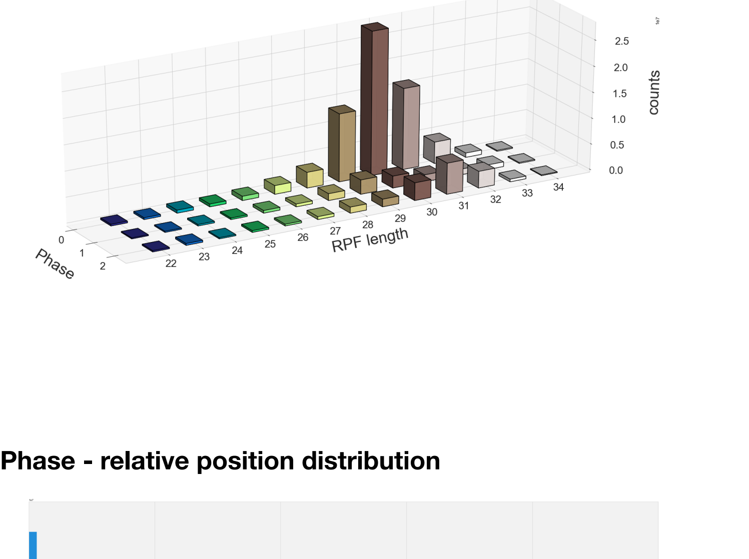

Phase - relative position distribution

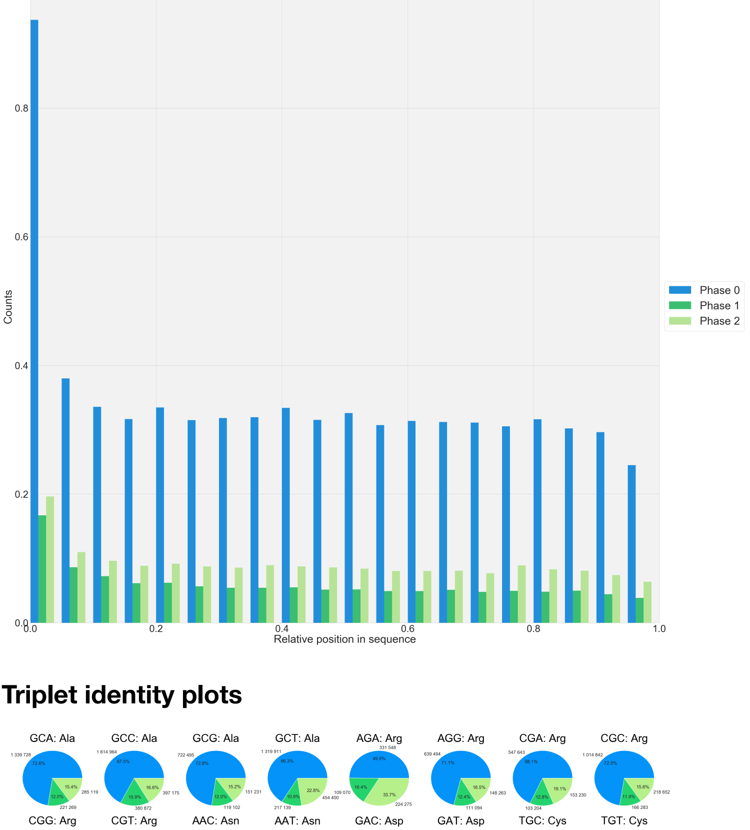

Triplet identity plots

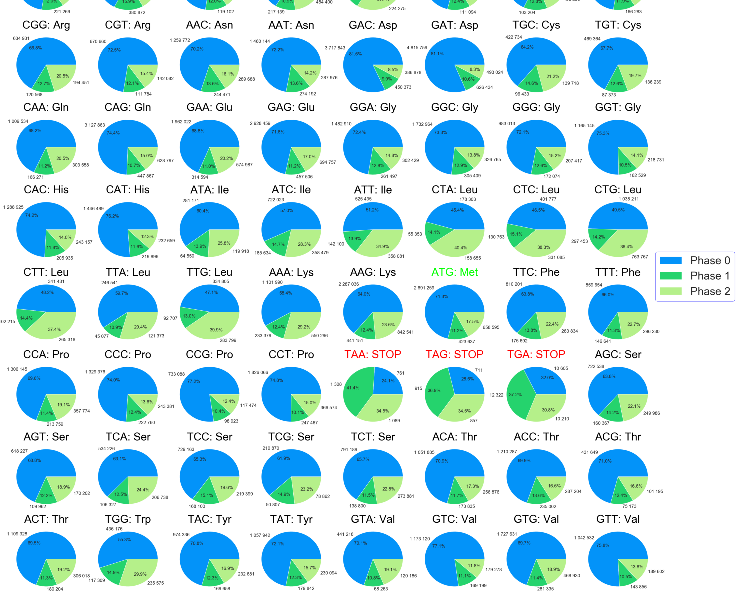

Codon usage plot

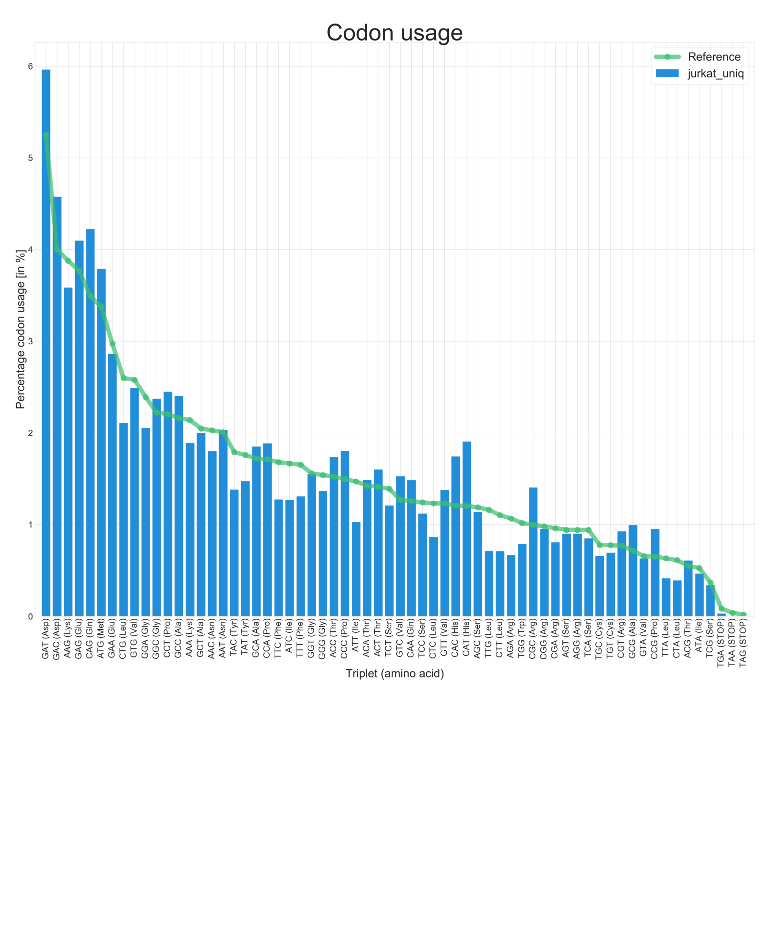

Supplement: Supplemental file S11 [file 142014_2_supp_322603_pqrw9l.pdf]
