# Supplementary material for: PROTEOFORMER 2.0: Further Developments in the Ribosome Profiling-assisted Proteogenomic Hunt for New Proteoforms
Source: Mol Cell Proteomics. 2019 Apr 30;18(8 Suppl 1):S126–40. doi: 10.1074/mcp.RA118.001218 (PMC6692777; doi:10.1074/mcp.RA118.001218)

Analysis information

Feature	Value
Species	human
Input sam file	../STAR/fastq2/treat.sam
Ensembl version	92
Ensembl database	../ENS_hsa_92.db
Sample treatment	treated
Mapping unique?	Y
Mapping first rank?	N
MappingQC unique?	Y
Selected offset source	plastid
Mapped genomic sequences	97 466 121
Analysis date	Friday 28 Sep 2018
Analysis time	01:18:24

Plastid offset analysis

RPF length	Offset
22	12
23	12
24	12
25	12
26	12
27	12
28	12
29	12
30	12
31	12
32	12
33	13
34	12

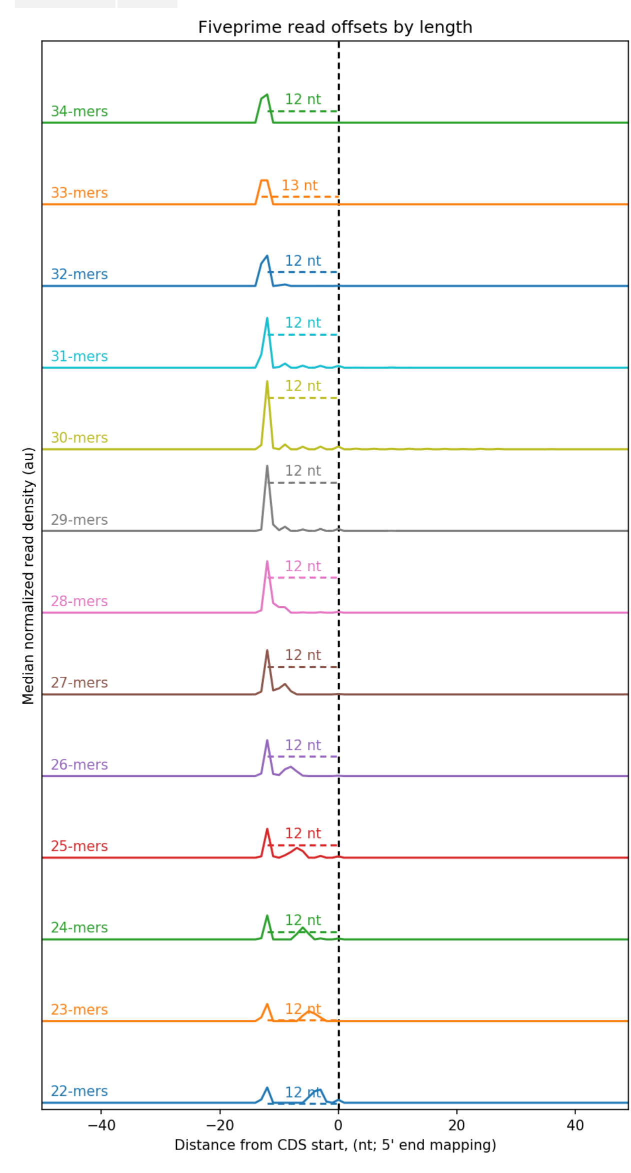

Gene distributions

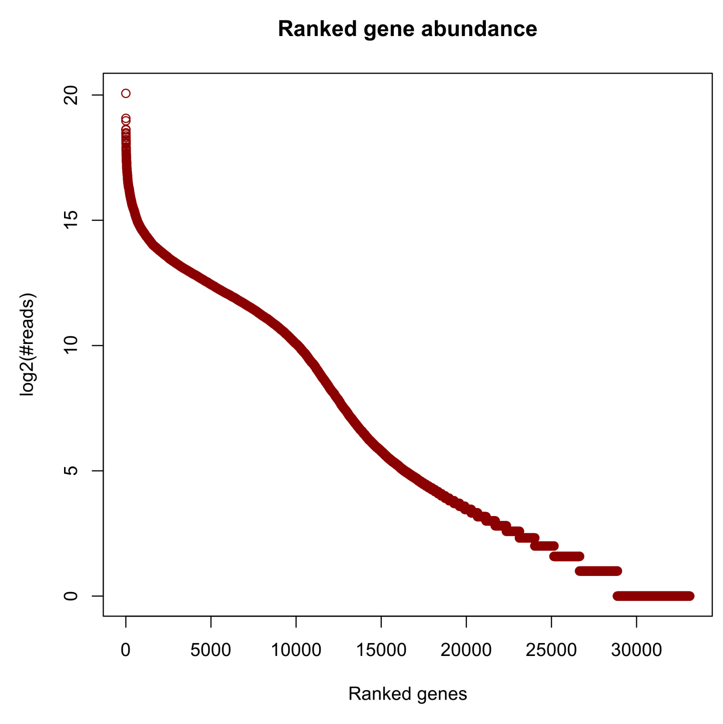

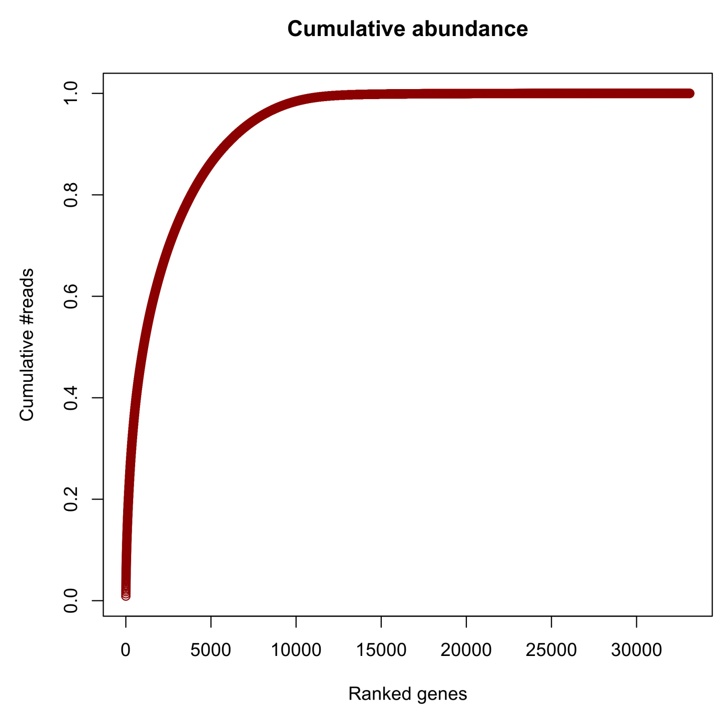

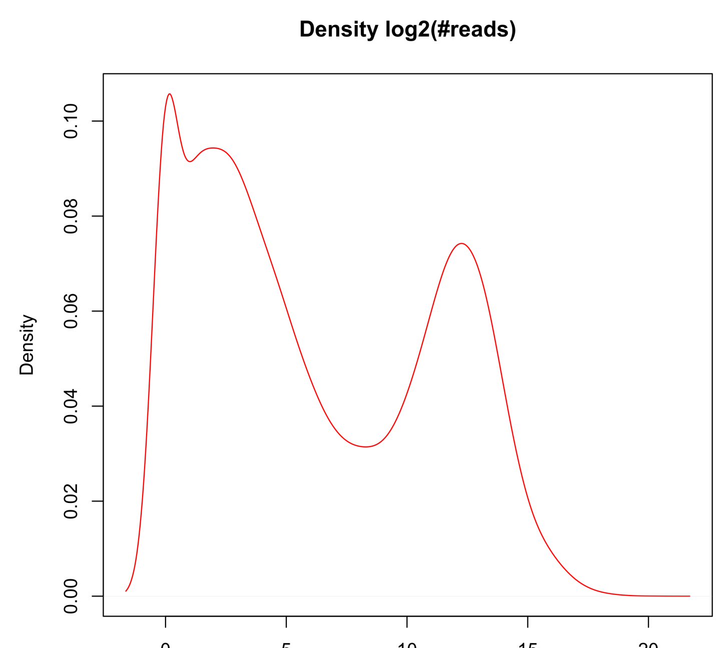

Metagenic classification

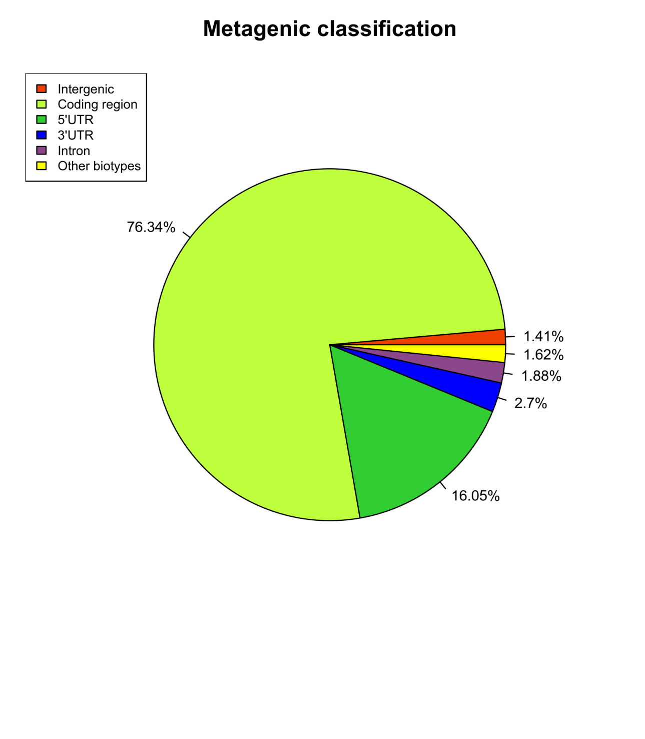

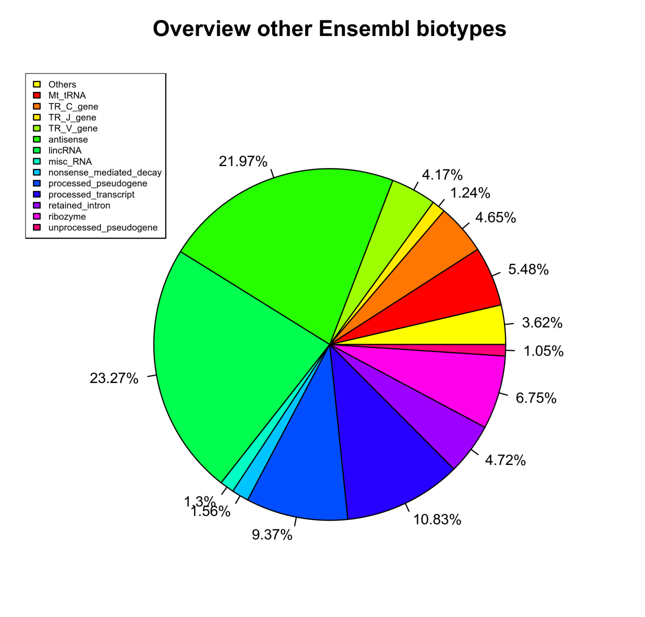

Total phase distribution

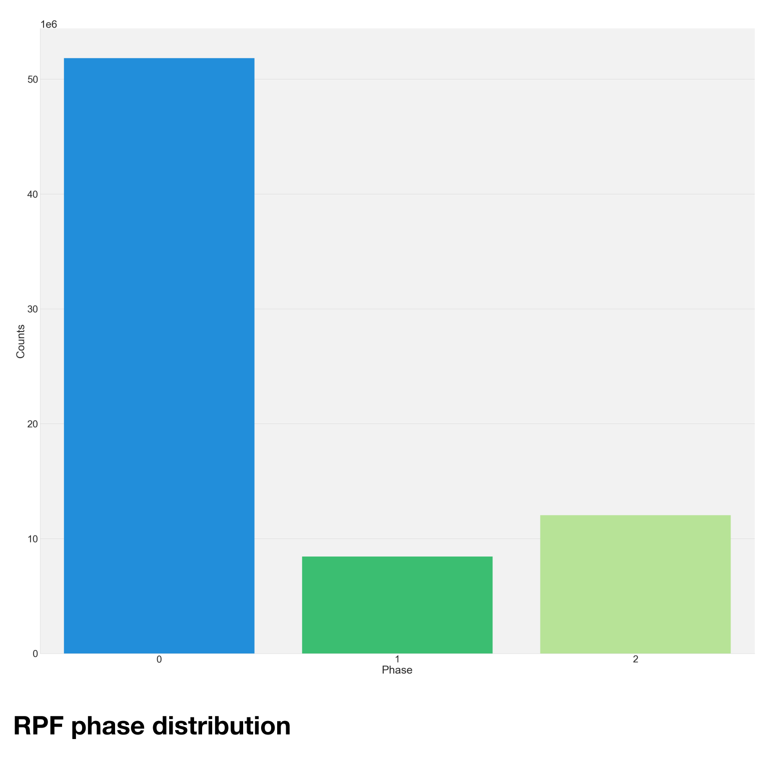

RPF phase distribution

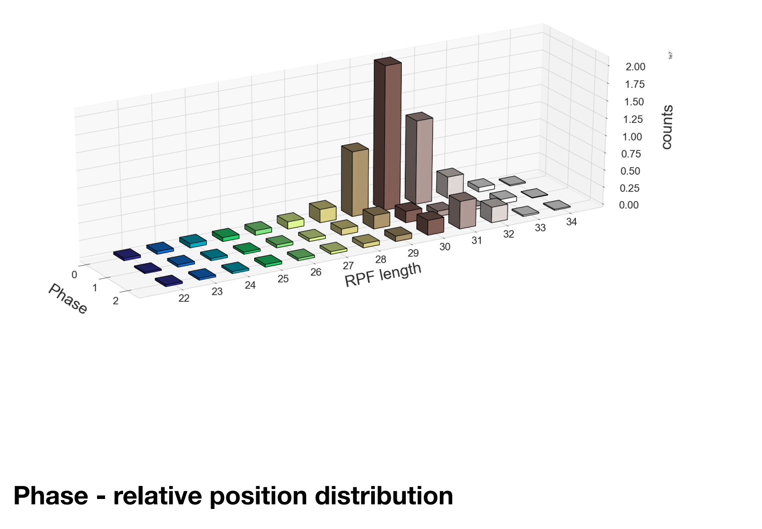

Phase - relative position distribution

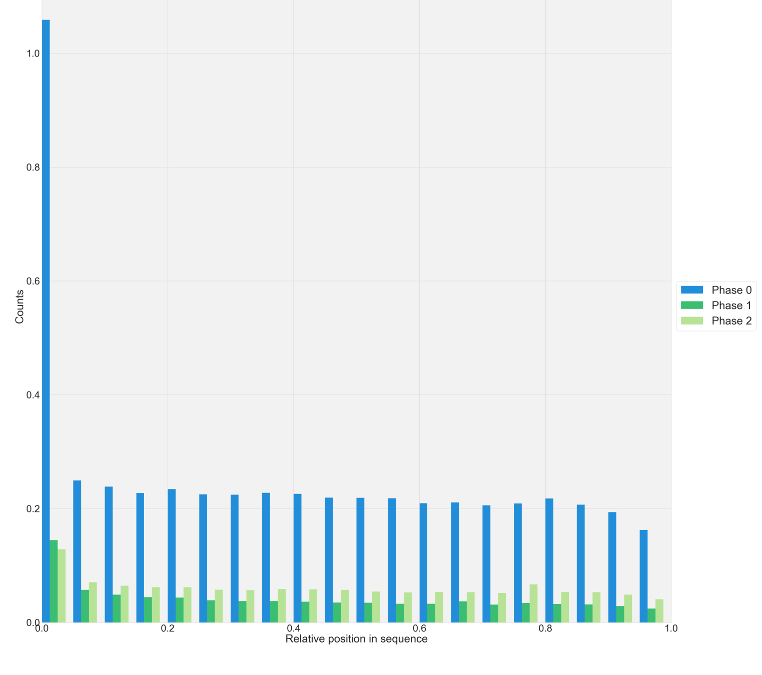

Triplet identity plots

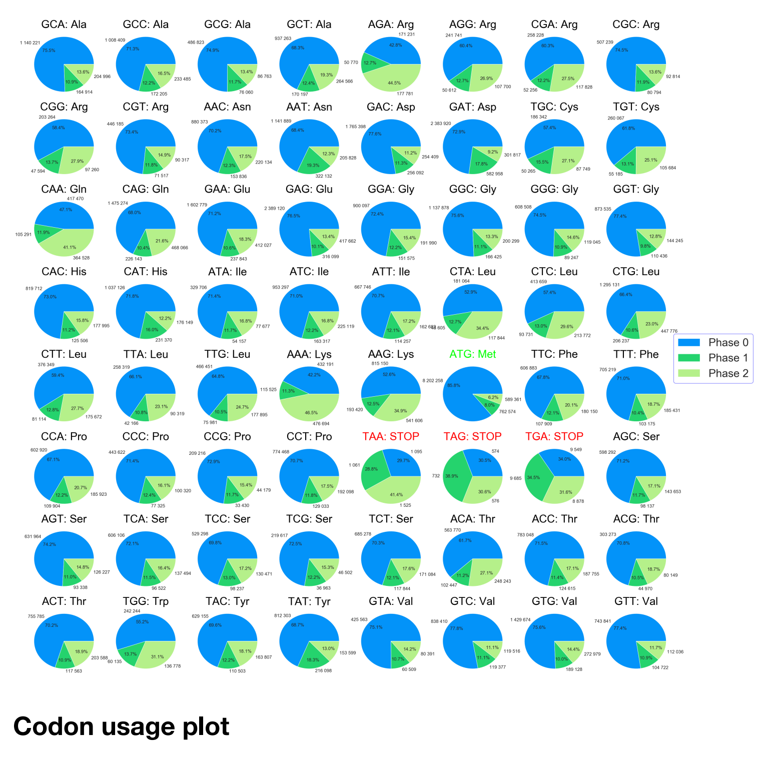

Codon usage plot

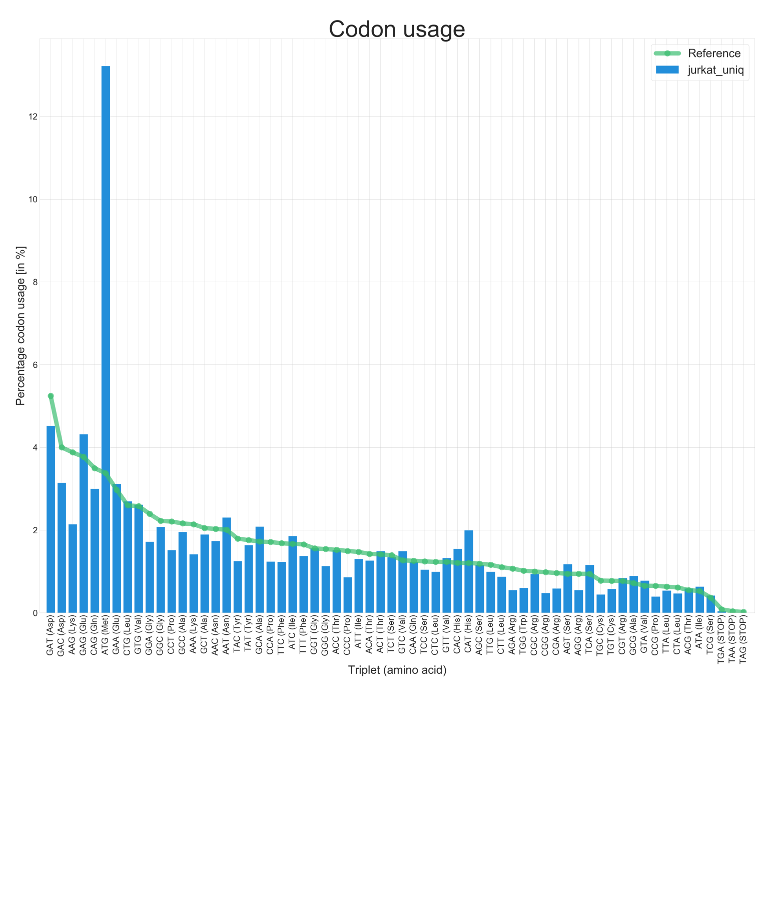

Supplement: Supplemental file S12 [file 142014_2_supp_322604_pqrw9l.pdf]
